# The Influence of Ionic Liquids on the Effectiveness of Analytical Methods Used in the Monitoring of Human and Veterinary Pharmaceuticals in Biological and Environmental Samples—Trends and Perspectives

**DOI:** 10.3390/molecules25020286

**Published:** 2020-01-10

**Authors:** Natalia Treder, Tomasz Bączek, Katarzyna Wychodnik, Justyna Rogowska, Lidia Wolska, Alina Plenis

**Affiliations:** 1 Department of Pharmaceutical Chemistry, Medical University of Gdańsk, Hallera 107, 80-416 Gdańsk, Poland; natalia.treder@gumed.edu.pl (N.T.); tbaczek@amg.gda.pl (T.B.); 2 Department of Environmental Toxicology, Faculty of Health Sciences with Institute of Maritime and Tropical Medicine, Medical University of Gdańsk, Dębowa 23 A, 80-204 Gdańsk, Poland; k.wychodnik@gumed.edu.pl (K.W.); justyna.rogowska@gumed.edu.pl (J.R.); lidia.wolska@gumed.edu.pl (L.W.)

**Keywords:** ionic liquids, green chemistry, environmental and biological samples, sample preparation, determination of pharmaceuticals, chromatographic methods, electromigration techniques

## Abstract

Recent years have seen the increased utilization of ionic liquids (ILs) in the development and optimization of analytical methods. Their unique and eco-friendly properties and the ability to modify their structure allows them to be useful both at the sample preparation stage and at the separation stage of the analytes. The use of ILs for the analysis of pharmaceuticals seems particularly interesting because of their systematic delivery to the environment. Nowadays, they are commonly detected in many countries at very low concentration levels. However, due to their specific physiological activity, pharmaceuticals are responsible for bioaccumulation and toxic effects in aquatic and terrestrial ecosystems as well as possibly upsetting the body’s equilibrium, leading to the dangerous phenomenon of drug resistance. This review will provide a comprehensive summary of the use of ILs in various sample preparation procedures and separation methods for the determination of pharmaceuticals in environmental and biological matrices based on liquid-based chromatography (LC, SFC, TLC), gas chromatography (GC) and electromigration techniques (e.g., capillary electrophoresis (CE)). Moreover, the advantages and disadvantages of ILs, which can appear during extraction and separation, will be presented and attention will be given to the criteria to be followed during the selection of ILs for specific applications.

## 1. Introduction

Analytical chemistry focused on the development of methods for the qualitative and quantitative determination of compounds with different chemical structures is a huge, dynamically developing field of science. The number of available methods and techniques is impressive. However, in addition to successes, there are many limitations regarding the use of such approaches. Problems may appear already at the sample preparation stage. Inadequate selectivity, and the use of large volumes of harmful organic solvents with a high vapor pressure in liquid-liquid extraction (LLE) or solid-phase extraction (SPE) are some of the many reasons for the search for alternatives [1]. The introduction of microextraction combined with the reduction of organic solvents used, and the inclusion of additional physical and chemical factors (sonication, temperature) have brought enormous progress, but also have several difficulties. Microextraction into both solid and liquid phases is a time-consuming process, and the final results require the indication of many other conditions [2]. For example, in solid-phase microextraction (SPME), commercially available fibers are not always suitable for the target compounds, while for single-drop microextraction (SDME), the stability of the drop in the sample may be a problem [3,4]. These limitations, as well as the need for even greater process control by affecting the retention time, and improving the extraction efficiency and resolution of analytes, are responsible for the attempt to include new structures in the extraction process, which can help to achieve these goals [5]. Modifications, such as the introduction of additional processes in liquid-based sample preparation procedures or changes on the surface of sorbents in SPE-based extraction and microextraction procedures are a good direction in analytics, but often insufficient to achieve the expected effects.

Equally as crucial as sample preparation is the process of the separation and detection of the compounds of interest. Among the many available techniques, chromatography or electrophoresis are most often used for the determination of pharmaceuticals in different matrices. Chromatographic techniques exist in a variety of types: the oldest thin-layer chromatography (TLC), the commonly used high performance liquid chromatography (HPLC) and gas chromatography (GC) as well as the less popular supercritical fluid chromatography (SFC) techniques. These methods can be coupled to various types of detectors, including ultraviolet (UV), fluorescence (FL) or mass spectrometry (MS). There are many important parameters during the development and optimization of methods but the most important include the choice of the stationary phase (the place of separation of the analytes) and the mobile phase composition. If the analytes show excessive column adsorption, tailing of the chromatographic peaks occurs and their width is incorrect [6]. In turn, when choosing a mobile phase, problems can occur with obtaining separate peaks for specific compounds, a too long analysis time and low efficiency [7]. However, other chromatographic conditions, such as the column temperature and the flow rate of the mobile phase as well as the parameters of detection should be carefully selected. This is a particular challenge for pharmaceutical determinations because their diverse structures and rich (despite extraction) matrices, and the necessity to detect many analytes at the same time, are just some of the reasons for difficulties in their separation. In addition, it should be highlighted that the mobile phases in LC often contain large volumes of organic solvents which are highly toxic. An interesting alternative seems to be electromigration techniques such as capillary electrophoresis (CE), micellar electrokinetic chromatography (MEKC) or non-aqueous capillary electrophoresis (NACE). These analytical approaches have been considered to be powerful separation methods due to low sample and reagent consumption, high efficiency, and simplicity. On the other hand, CE-based methods have relatively low sensitivity which makes their application difficult in real clinical and environmental studies. Thus, the above examples show that each stage in the development of an analytical method (both sample preparation and further analysis) can cause problems in performing experiments or in achieving reliable results.

**Figure 1:**
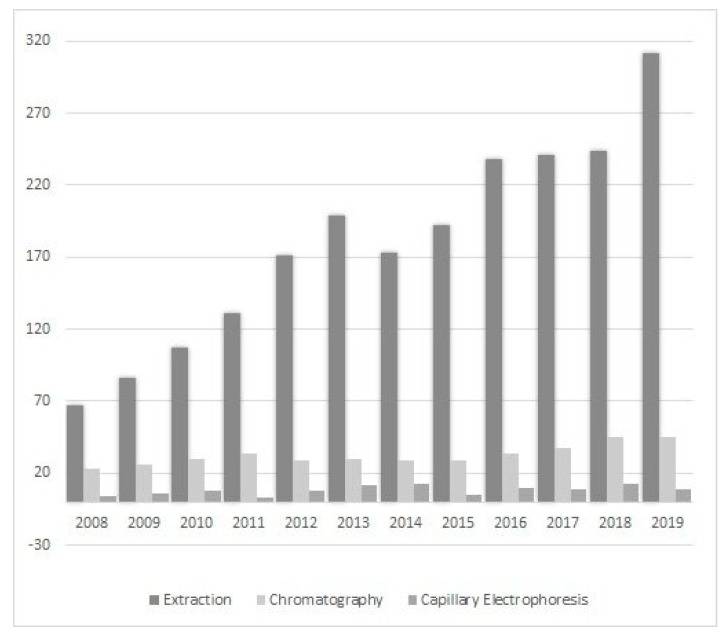
Number of publications on the use of ILs in sample preparation (extraction and microextraction) and chromatographic and electrophoretic techniques in 2008–2019 (the authors own elaboration according to ScienceDirect data).

Ionic liquids (ILs) are a relatively new class of compounds that became an object of special attention in the 21st century. Their simple cationic-anionic structure provides unusual and unparalleled properties. Therefore, it should not be surprising that their potential is exploited in many unrelated areas of science, for example, as a catalyst in chemical reactions [8], in drug delivery systems [9], in electroplating processes [10], in treating harmful compounds in wastewater [11], as matrices for analysis by matrix-assisted laser desorption/ionization mass spectrometry (MALDI-MS) [12] and many others. Scientists have also become interested in “designer solvents” in response to the constant demand for developing new and better methods, and improving the results obtained. The literature data show that their application is focused on sample preparation by extraction or microextraction as well as chromatography (adding ILs to the mobile phase or to prepare the stationary phase) and electrophoretic techniques (Figure 1).

In pharmaceutical sciences ILs can be used for a variety of purposes: as active pharmaceutical substances (API-IL) [13], to determine the solvent residues and impurities in drug quality testing [14] or as a source of information about the presence of pharmaceuticals in biological and environmental samples [15,16]. An important argument supporting their use was also the introduction by Anastas in 1999 of the 12 principles of green chemistry [17]. Attention was drawn to the excessive use of organic solvents and the need to eliminate or reduce environmentally harmful factors. The search for alternatives resulted in the inclusion of ILs in experiments. Negligible vapor pressure, non-flammability, thermal stability, and the possibility of reuse are just some of the properties that have allowed ILs to be described as more environmentally-friendly [18]. It should be highlighted that as newer compounds their literature data are incomplete. However, this does not preclude their use at various stages of analytical testing, from sample preparation to detection and the improvement of results, even for difficult to determine analytes, including the quantification of pharmaceuticals in biological and environmental samples. These substances, with different pharmacokinetic activity, can be delivered directly and indirectly (animal-derived foods) to the human body in very low concentrations. Moreover, pharmaceutical concentrations in urine or bile are different from those in blood or saliva [19]. For this reason, it is necessary to develop a method that will be adequate for the specific biological sample. In the treatment of patients, combination therapy is often used, which results in the presence in the matrix sample of many drugs with different physical and chemical properties making it difficult to choose the best extraction and separation conditions. It should also be remembered that these are not always stable compounds, and to obtain information on their concentrations, it may also be necessary to determine the degradation products and/or metabolites in the presence of many endogenous matrix compounds [20]. Similar considerations can be made in the field of drug determination in environmental samples. According to the data reported in the literature, the sources of pharmaceuticals in wastewater, river waters, lake waters and others are improper drug disposal, hospital wastewater or animal feces. If they occur in an unchanged form, they may cause the risk of typical side effects after they enter the body. One group of drugs often identified in environmental samples are antibiotics, which may be responsible for the development of antibiotic-resistant bacteria [21]. As in biological samples, pharmaceuticals are present in the environment in very low concentrations. Sample purification, the isolation of analytes or the possibility of enriching the sample are crucial and influence the final efficiency of a method. As already mentioned, pharmaceuticals are compounds with high biological activity, so it is also important to develop simple, reproducible, quick methods, without the need to introduce additional steps to improve the safety of analysts [22]. The inclusion of ILs in their analyses not only improves safety due to the reduction of the use of organic solvent, but also, as confirmed by research, helps to overcome the mentioned difficulties in the analysis of drugs and to improve the validation parameters and efficiency. Therefore, the monitoring of these substances in both the environment and animal and human samples using IL-based environmentally-friendly analytical methods, which also offer reliability, and the qualitative and quantitative sensitivity and selectivity of the compounds of interest is one of the main tasks of modern analytics and chemistry.

The growing number of research papers on ILs has also increased interest in this topic in review articles. Their wide spectrum of possibilities is also clearly visible in the huge variety of subjects of such works. Some of them focused on IL in the context of “green chemistry”, pointing to their great potential, but also disadvantages (the need to remove them from the environment, multi-stage synthesis) [23,24]. The reviews very often summarized their applications in sample preparation, especially solid phase microextraction. Most commonly, polymeric ionic liquids (PILs) were evaluated in such applications [25,26,27,28]. Some articles considered all the possibilities for using ILs, both at the extraction and detection stages [29,30,31,32]. However, the publication selection criteria in the review papers most often concerned analytical methods and techniques or the type of ILs and did not focus on the specific type of analytes or matrices. In addition, it should be noted that the dynamic development of analytical methods using IL requires continuous monitoring of current scientific reports and providing the latest information in current reviews papers. Thus, the purpose of this review was to summarize achievements in the use of ILs for the determination of drugs in biological and environmental samples. In order to properly understand the popularity of ILs in the modern laboratory, the section ‘‘Ionic Liquids’’ presents their history, with the inclusion of their most important features and properties. The basic criteria for choosing articles for the review was the use of ILs during the sample preparation procedure or in the chromatographic/electrophoretic separation of synthetic drugs quantified in biological and environmental samples. The review did not include endogenous compounds, substances responsible for addiction (e.g., nicotine and others) and herbal medicines, except for IL-applications in GC, TLC and SFC. This extension was made in order to fully present the capabilities of ILs and show current trends in the determination of different active biological substances.

## 2. Ionic Liquids

**Figure 2:**
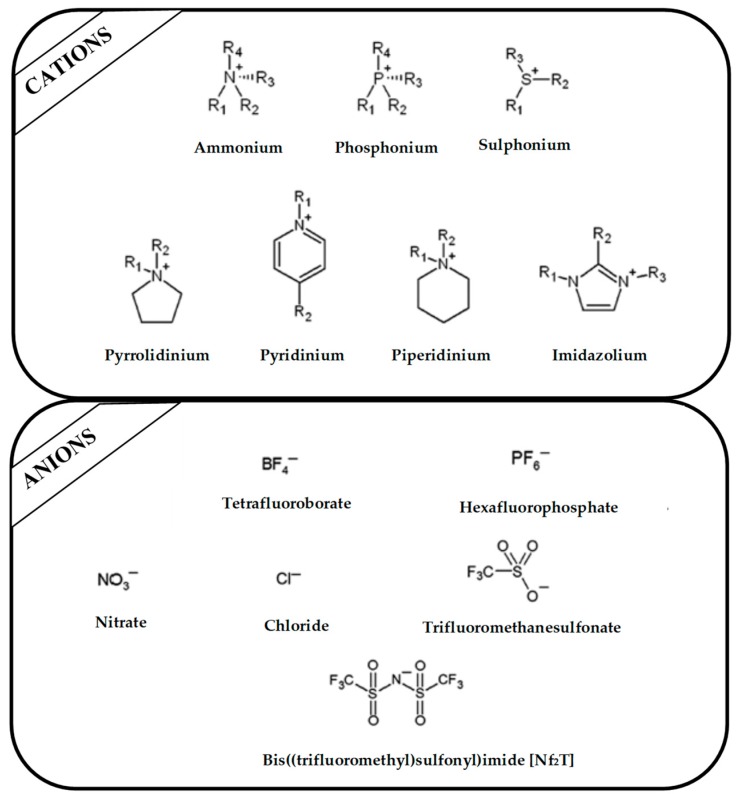
Examples of popular anions and cations of ILs used in analytical methods.

Regarding the history of ILs, and events that are responsible for their presence in many fields of science, it is first of all necessary to define the criteria used in the presentation of this subject. Considering the period of their greatest popularity, that is, the last two decades, we can accept the work of scientists who in their publications focused primarily on modifications of compounds in order to obtain the desired properties and identify their applications. However, to acquire information about the discovery of compounds that were ILs, although no one was aware of this and such a definition was not used, we should return to the mid 19th century. At that time, a by-product known as “red oil” was obtained in the Friedel-Crafts reaction. As shown later, this was the first recorded IL [33]. In the following years, Gabriel and Warner also made an important contribution to the development of ILs. In 1888, for the first time, they synthesized ethanol-ammonium nitrate [34]. Although all previous events were very important, the synthesis of ethylammonium nitrate by Walden in 1914 has most often appeared in publications in the context of the discovery of ILs [35]. Of course, it should be mentioned that in the case of ILs, as in all great discoveries, there are opinions that although Walden synthesized the compounds, he could not use them in practice and his success is over-emphasized [36]. Nevertheless, it was undoubtedly an important stage in the development of ILs. During the following years, there were further syntheses of the compounds and attempts to use them, among others by Yoke and his colleagues [37] and Koch and co-workers [38]. However, in more modern times, with the current compounds that are used in research, it is necessary to focus on analytical methods and extraction techniques. Considering the application of ILs, Pool’s research should be mentioned, in which, using current knowledge, ILs were used in GC as stationary phases [39]. The results of this study prompted the beginning of their further development in this field and became the inspiration for subsequent publications. The 1980s were also important due to the synthesis of ILs based on the imidazolium cation, which are currently widely used in laboratories [40]. This event was important because the existing compounds of ILs had significant limitations in their application, while the imidazolium group provided new opportunities for researchers. Following the trend, subsequent years of research into the use of ILs increased knowledge about them, and consequently led to the introduction of some standards in this area. At the turn of the 20^th^ century, ILs began to function under the name Task-specific Ionic Liquids (TSILs) [41] and companies marketing the first commercially available ILs appeared [42]. Increased access and the positive opinion of the scientific community prompted attempts to apply them to novel projects. In 1998, for the first time, ILs were used as extractants for LLE [43], and in 2005, they were used to coat SPME fibers [44]. Recent years have seen a period of their participation in advanced research, but this will be discussed in detail in subsequent sections. However, it should be highlighted that the most important factor responsible for the rich and long history of ILs is their specific structures, illustrated in Figure 2, which provide the enormous possibilities of these compounds. The cation-anion combinations, described in most definitions, create many possibilities for structure modification, and can thus change the properties of the designed compounds. The cations may have one or more nitrogen, sulfur, phosphorus or oxygen atom in the structure, described as ammonium, sulfonium, phosphonium or oxonium cations, respectively, but in most cases, they are large organic aromatic moieties: pyridinium, piperidinium and the most widely used imidazolium cations. In turn, anions are much smaller and can be both organic and inorganic. In research, tetrafluoroborate ([BF_4_]), hexafluorophosphate ([PF_6_]) and halogen anions, and many other compositions appear.

Besides the selection of the cation and anion, an important aspect that affects further results is the substituents on the cation, and especially the alkyl chain, the length, branching and position of which have a huge influence on applications of ILs [45]. To fully understand the unique properties of an IL, it is also necessary to pay attention to Coulombic interactions occurring in the molecules, dipole-dipole interactions, Van der Waals forces and hydrogen bonds [46]. It is estimated that the number of available combinations may allow up to 1018 different ILs to be obtained [47]. The differences in the size of the cation and anion, the asymmetry in the structure as well as the mentioned interactions mean that they have no regular, crystalline structure and the delocalization of the cation and anion composition is very possible. Thanks to this, their melting temperature does not exceed 100 °C, and in many cases it is close to room temperature (RTIL) [48]. This feature distinguishes ILs from typical inorganic salts, which, due to the much stronger Coulombic and hydrogen interactions, have a melting point of even above 400 °C. Equally as interesting as their melting point is the viscosity of ILs, which is at a higher level than that of organic solvent. Knowledge of these parameters is necessary when an IL is used in separation and detection techniques. The electrostatic interactions in alkyl chain cations have an enormous impact on viscosity. Coulomb forces, H-bonding and π-π dipole lead to increases in the flow resistance, and additionally, the presence of van der Waals interactions between the cation and anion, depending on the size of the molecule, also causes interactions in the same direction. This property can be modified by changing the temperature or adding an organic solvent [49,50,51,52]. Viscosity also influences another property, namely electrochemical conductivity. Thanks to their ionic structure, ILs can carry a charge, but this possibility is not the same for all compounds. When the flow resistance increases, conductivity becomes more difficult. However, increasing the temperature and mixing with organic solvents improves the results. Furthermore, the size of molecules can hinder access to the charge, so it is necessary to select the appropriate cations, which are large ions [53]. ILs are widely used in sample preparation techniques because they can be created as both hydrophilic and hydrophobic compounds that mix with water, and/or organic solvents [54]. It has been proven that the change in the position of the methyl group in the cation determines the change in the acid-base character, and therefore the C2 position is strongly acidic, which affects the interaction with other compounds [55]. The thermal stability of ILs is also important. As studies have shown, the majority of popular ILs are stable even above 300 °C, which is of great importance during GC analysis, where a high temperature is required. As with previous properties, the size and type of ions, pKa, chain length and electrostatic interactions determine the stability of individual ILs. Halogen anions, probably due to their nucleophilic character, have less stability than other inorganic anions, while the most stable is bis(trifluoromethanesulfonyl)imide ([Nf2T]). In turn, among the cations, the stability of pyrrolidinium and piperidinium is lower than that of imidazolium, regardless of the anion used [56,57,58]. An interesting property is also the insignificant vapor pressure which occurs at elevated temperatures. Zaitaus et al. confirmed the influence of the structure of ILs on vapor pressure. In their study, the absolute vapor pressures for a series of [C_n_MIM][BF_4_] ionic liquids with (n = 2, 4, 6, 8, and 10) were measured. The results of experiments confirmed that an increase in the number of carbon atoms in the alkyl chain in the imidazolium cation caused a decrease in absolute vapor pressures. However, this effect was different for the homologies of [C_n_MIM][BF_4_] and [C_n_MIM][Nf_2_T]. Moreover, it was observed that the volatility for [C_n_MIM][BF_4_] was significantly lower in comparison to [C_n_MIM][Nf_2_T]. In addition, ILs added to organic solvents also reduced their evaporation [59,60,61].

It should be noted that there are a huge variety of IL combinations, so it is difficult to establish a clear classification. The most popular approach concerns the structure of these compounds (Table 1). A more detailed description concerns three generations of ILs in view of the anion or cation used. The first includes molecules with specific physical properties, which are described in the previous paragraph. The second generation includes ILs for which it is possible to tune their chemical and physical properties and then to use them for a specific purpose, while the last group are compounds with biological activity [62]. From the point of view of analytical applications, it seems reasonable to focus attention on a large and diverse IL group referred to as Task-specific Ionic Liquids. The results of subsequent tests confirmed that apart from typical ILs, it is necessary to design more specific molecules to achieve a specific goal. This led to the use of ILs in polymerization processes. ILs as monomers can form combinations with other molecules, thus improving the results.

The currently applied analytical methods use the structure and properties of ILs to create polymeric connections with cyclodextrins (CDs) [63] or magnetic imprinted nanoparticles (MILs) [64]. In addition, polymeric ionic liquids (PILs) can also be synthesized by a co-polymerization process [65]. Their participation in the molecular imprinting technique used to develop sorbents of monolithic columns has also been noted. Another, large subclass of ILs are chiral ionic liquids (CILs). Recent scientific reports show that amino acids can be used for the synthesis of CILs. Their carboxyl or amine functional groups determine the chiral nature and function in the structure (cation or anion). The use of amino acids results from the trend of reducing toxicity and the use of natural compounds [66]. In addition, these “designer molecules” are also used as chiral selectors in aqueous two-phase systems (ATPS) [67].

## 3. Sample Preparation

As it was earlier mentioned, the sample preparation procedure is still one of the most important stages in the development of analytical methods. The variety of biological and environmental samples makes them very complicated with regard to gathering all information about the sample preparation stage. Both types of samples are complex analytical matrices, and the stage of their preparation for analysis is multifactorial. It usually requires the performance of various operations and activities both in situ and in the laboratory. Due to the very low concentrations in real samples, the extraction method should have the highest possible recovery. In addition, the sample handling method largely depends on the chosen final determination technique. Knowing the chemical properties of the drug (or drugs) sought in the analyzed matrix, makes it possible to properly select the organic solvents in order to carry out a successful extraction from the sample, followed by purification, sometimes by back extraction. Considering all these aspects, it is necessary to search for new directions in sample pretreatment procedures. One of these is the use of ILs at the preparation stage of biological and environmental samples to isolate the drugs potentially present in them, both with the use of liquid-liquid based extraction and solid-phase based extraction procedures [68]. ILs are used as liquid phases, extractors, intermediate solvents, mediators and desorption solvents [68,69,70,71,72,73,74,75,76,77,78,79,80,81,82,83,84,85,86,87,88,89,90,91,92,93,94,95,96,97,98,99,100,101,102,103,104,105,106,107,108,109,110,111]. Exemplary applications of individual types of drug extraction from biological and environmental samples with IL-modifications are presented in Table 2. These summarized data clearly indicate that despite the determination of low pharmaceutical concentrations in both types of samples, IL-based extraction procedures go in a different direction. If the matrix is biological fluid, the most common problem is the distribution of peaks, selectivity, shape and of course performance. In turn, environmental samples most often focus on the need to improve extraction efficiency [69,70]. Matrix influence, peak shape and distribution are not the main reasons for using ILs in extraction. A difference also occurs in the volume of the analyzed sample, being much larger for environmental samples [69]. This factor is especially important during the formation of two phases with the participation of ILs, in which the proper volume ratios (aqueous phase, organic phase, IL and others) are needed for the proper phase separation and the subsequent separation [67,112,113,114]. In the publications presented below, it can also be seen that for environmental samples, there was a much greater variety in the choice of extraction method, especially in the area of dispersive liquid–liquid microextraction (DLLME). In the case of biological samples, they were also extracted by DLLME, but the modifications were much smaller in number.

### 3.1. Liquid-Phase Based Extraction and Microextraction Procedures

#### 3.1.1. Liquid-Liquid Extraction

LLE is the oldest method of extracting analytes. Unfortunately, despite the simplicity of performance, the method has many disadvantages. It is a very time- and work-intensive process, and the results depend on many additional factors, e.g., the physicochemical character of analytes, the type of extraction solvents, the extraction time and the temperature, which in turn cause reproducibility and repeatability problems. Furthermore, according to the current trend of designing more environmentally-friendly analytical methods, the use of toxic organic solvents should be reduced. As is well known, LLE does not meet this condition. In all probability, this was the reason why in regard to drug quantification in biological and environmental samples, traditional LLE extraction supported by IL modification was rarely considered. To the best of our knowledge, no reports have been published for biological applications, while only one paper can be found in the field of environmental investigations (Table 2).

##### Environmental Samples

Kiszkiel et al. [68] were the only researchers who tested the ionic liquids [C_4_MIM][PF_6_] and [C_4_MIM][Nf_2_T]) for LLE, showing their ability to selectively isolate nizatidine and ranitidine from wastewater and river waters (Table 2). Based on preliminary studies, an IL with a different anion was selected for each analyte. In the case of nizatidine extraction, [C_4_MIM][PF_6_] was used, while for ranitidine—[C_4_MIM][Nf_2_T]. Their application allowed methanol consumption to be reduced (1.0 mL for nizatidine and 1.5 mL for ranitidine). During optimization, the appropriate volume of IL was selected and the impact of additional factors such as the effect and mixing time or pH was assessed. The ultimately optimized and validated method allowed over 100% recovery, a wide range of linearity and low LOD values to be obtained for both analytes. Thus, in this paper, the parameter values confirmed that LLE using ILs allows satisfactory results to be achieved.

#### 3.1.2. Dispersive Liquid-Liquid Microextraction and Modifications

ILs have more often been applied in new solutions based on liquid-phase microextraction (LPME), and particularly in the increasingly used dispersive liquid-liquid microextraction method (DLLME), introduced for the first time by Rezaee [69]. The most important elements of the most popular microextraction methods are two solvents: extractant and disperser. After their quick injection into the sample, the disperser solvent causes the dispersion of the extraction solvent in the form of fine droplets. The large surface contact with the analyte helps in its adsorption. Then the two formed immiscible layers can be easily separated from each other. The method has many advantages, above all, lower consumption of organic solvents, a faster process and greater sample enrichment. Thus, subsequently, positive properties introduced further modifications leading to even better results. As it was mentioned above, one of them was the use of ILs.

##### Biological Samples

**Figure 3:**
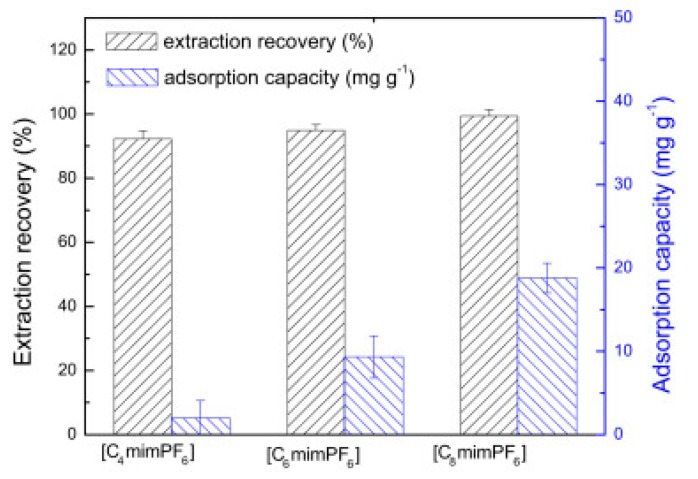
Effect of the kind of extraction solvents on ER of UPA and adsorption capacity. Extraction conditions: sample volume, 10.0 mL; sample amount, 10.0 µg; pH, 8.0; ultrasonic temperature, 313 K; ultrasonic time, 10 min; cooling temperature, 278 K; cooling time, 15 min; centrifugation time, 5 min. The error bars were standard deviation. Figure adopted from the reference [75] with copyright permission.

Cruz Vera and his colleagues [70] were among the first to use ILs in the DLLME of drugs from biological samples. In one-step in-syringe extraction of non-steroidal anti-inflammatory drugs (NSAIDs) from human urine, they used ILs as the extraction solvent and methanol as the disperser solvent. During optimization, they took into account not only the extraction efficiency, but also the enrichment factor and repeatability. Subsequent publications using IL-DLLME are modifications of the matrices and pharmaceuticals (Table 2). However, several repetitive elements of the study can be observed. First, the same group of molecules with the [PF_6_] anion and the imidazolium cation were most often used to select the ionic liquid with the best results. Differences were related to the length of the cation alkyl chain (1-butyl-3-methylimidazolium ([C_4_MIM]), 1-hexyl-3-methylimidazolium ([C_6_MIM]) and 1-octyl-3-methylimidazolium ([C_8_MIM])) [15,70,71,72,75,76,77,78,79,84,87,88,89]. Most often, ILs with a butyl or octyl substituent were qualified for further testing. Probably, the reason for choosing the C4 alkyl chain was the reduced viscosity and the resulting greater transfer of analytes to the IL (compared to C6 and C8) [15,70,71,76,78,80,81,82,87,88,89,90]. On the other hand, as the alkyl chain length increases, solubility in aqueous solutions decreases, and the analyte availability increases. This seems to be the reason for good results for [C_8_MIM][PF_6_] IL [72,73,74,75,79,83,84] (Figure 3).

However, it should be highlighted that despite the knowledge of the structure-properties, using only one criterion when choosing an IL is impossible. Thus, there is also the opinion that the structures of analytes should influence their choice. Moreover, the volume of ILs is an important factor. In many studies, it has been confirmed that as the volume increases, the efficiency and enrichment factors increase. However, the trend changes at some point and when the volume is too large, the results decrease. Probably, a large volume of ILs reduces the concentration of the analytes. On the other hand, if the volume is too small, the extraction and collection of the IL-analyte phase from the system is also problematic [71]. Therefore, this parameter should also be estimated in each study.

Besides those mentioned above, scientists also tried to include in the study ILs consisting of chloride ([Cl]), bromide ([Br]), [Nf_2_T] and methyl sulfate ([CH_3_(SO_4_)]) anions [78,80,81,82]. Only in one publication was there an attempt to replace the imidazolium cation with 1-butyl-1-methyl-pyrrolidinium ([C_4_MPyrr]) and 1-butyl-3-methylammonium ([C_4_M_3_Amm]) [78]. An important factor presented in the literature is the combination of ILs with organic solvents. Most often they are the dispenser solvent, but they can also be the solvent in back extraction [15,70,74,75,76,77,78,79,83,84,85,86,89]. It is known that ILs are highly viscous compounds. This property can hinder the chromatographic separation and detection of compounds, so the use of acetonitrile, methanol or ethanol is necessary. In some extractions, organic solvents are completely eliminated using instead sonication, controlled temperature, or intensive mixing. Their application helps to disperse ILs and gives as good results as organic solvents [15,74,76,77,78,79,83,84,85,86]. Gong et al. [75], during the determination of ulipristal acetate, completely eliminated the organic solvent as a dispersing agent. They used ultrasound energy without an organic solvent to disperse, and obtained an extraction recovery over of 95%. The addition of inorganic salts is also used in many works. The salting out process may affect the final results due to ionic strength and associated reactions with H_2_O molecules [71,89,91]. The choice of pH is also important, the goal being to have analytes in neutral form, because in ionic form there is less availability for ILs, and the final extraction efficiency decreases [85]. As mentioned before, in addition to several constant elements, there are also several variables, such as analytes and matrices. Studies usually extract drugs commonly used to treat humans and animals, including antibiotics [81,82,86,87,88], antidepressants [78,79,83], benzodiazepines [15,76,77] and NSAIDs [70,85]. The matrices are most often human urine, plasma and serum (Table 2). An extraction method for a unique kind of matrix was developed by De Boeck and co-workers [15,76,77,78]. As the authors of several articles related to the use of ILs in DLLME, they started from choosing the best extraction and detection conditions for the determination of benzodiazepines, benzodiazepine-like hypnotics and antidepressants in whole human blood by LC-MS/MS. Then they transferred the optimized conditions for the analysis of postmortem blood samples. Both the matrix type and LC-MS/MS were first used in an IL-based analytical method for determining pharmaceuticals. Drugs used in veterinary medicine were determined in milk, eggs and the meat of pigs, cows, chickens and fish [87,88].

##### Environmental Samples

Similar to biological samples, two methods of the DLLME procedure can be observed: traditional, using only an IL (extractant) and organic solvent (dispersant) [92,93] or modified, using additional steps, such as ultrasound and others [94,95,96,97,98,99,100,101,102,103]. The traditional method was used by Yao et al. [92], who, by performing analyses with different ILs, drew attention to the impact of the character of the analyte on the final results. An IL with the [Nf_2_T] anion and basic properties allows higher efficiency extraction for acidic compounds, whereas for compounds containing tertiary amines, the [TFP] anion was better. To further explain this phenomenon, the effect of surfactants on the results was also investigated. The use of the popular sodium dodecyl sulfate (SDS) without a primary amine did not improve the extraction efficiency, but after using a surfactant having such a moiety the result improved significantly. DLLME without modification also allowed the determination of triclosan and triclocarban by Zhoe et al. [93]. During optimization, [C_6_MIM][PF_6_] was chosen for the analysis because of the higher solubility in water and worse efficiency of the [C_4_MIM] cation. The researchers also noted that the addition of an inorganic salt (most often NaCl), which changes the ionic strength, is responsible for two opposite effects. On the one hand, the addition of NaCl causes an increase in the solubility of an IL in water, thus increasing the volume sedimentation phase and consequently, the efficiency decreases, but on the other, there is an increase in analyte enrichment. Thus, the choice of this additive is not obvious.

DLLME modifications in the extraction of environmental samples are much more common. One of them is the use of ultrasound. Parrilla Vázquez and co-workers [97,98] focused on the optimization of this stage. They highlighted that the sonification time (too long may cause degradation) and sample cooling after the process have an impact on improving the results. Mao et al. [95] used high energy ultra-sound instead of normal ultrasound. In all US-IL-DLLME methods, the ILs for further analysis were selected from among the group with imidazolium cations and anions [PF_6_] in their structure. The best results were always obtained for ILs with the highest hydrophobicity, therefore the longest alkyl chain. Another modification was the inclusion of SDS in addition to the IL. The surfactant aimed to improve performance by reducing the adhesion of the IL to the walls of the tube. In addition, the novelty was heating the sample to 30 °C after the addition of the IL to completely dissolve the IL and then cooling to form two phases [96]. Yu et al. [94] used MIL to extract various compounds, including pharmaceuticals. They chose the best IL according to several criteria, such as magnetic susceptibility, HPLC compatibility, hydrophobicity needed for phase separation, minimal IL absorbance and minimal anion hydrolysis in the aqueous phase. These conditions were met by [P_6,6,6,14_^+^]_2_[MnCl_4_]. In order to achieve high efficiency, microwave energy was also used. However, its use could both improve and worsen the results, depending on the volume. Too high a temperature increases the contact of the IL with the aqueous phase and reduces the volume of the sedimentation phase, in consequence reducing the efficiency. The paper also discussed the influence of the dispersant on the final results. The choice of its volume is crucial as too large a volume causes an increase in the solubility of the IL in water, while too small hinders the formation of two phases [100]. Aimed at achieving environmentally-friendly procedures with the best results, methods using two ILs have also been proposed. Toledo-Neira et al. [103] used both [C_4_MIM][BF_4_] and [C_4_MIM][PF_6_] to change the polarity of the sample, and as an extractant, respectively. However, this work also uses an organic solvent as a dispersant. In another article with two ILs, one hydrophilic IL was used to disperse the other hydrophobic IL. Finally, only 50 µL of MeOH was used in the method to dissolve the sample prior to HPLC injection, thus the organic solvents were almost completely eliminated [102]. The last method of modification in the context of environmental samples was to combine DLLME with SPE. However the IL, as previously, was only applied as the extractant in DLLME. Among the tested ILs, the best result was obtained with [C_6_MIM][TFP] regarding the highest hydrophobicity, which was a constant trend in similar papers [101].

#### 3.1.3. Other Liquid-Phase Extraction

##### Biological Samples

Good research results have encouraged researchers to further modify their extractions with ILs (Table 2). In 2015, doxepin and perphenazine were extracted according to a new procedure: ionic liquid-based surfactant emulsified microextraction accelerated by ultrasound radiation (IL-SE-UE-ME) [104]. Together with ultrasound applied to the surfactant, this led to the creation of an emulsion with the participation of ILs. The following year, Liu and co-workers [105] determined sulfonamides and used two ILs for extraction. They were the first to add [C_4_MIM][PF_6_] together with an inorganic salt, and after forming the precipitate, they added [C_6_MIM][PF_6_]. As a result, the analytes could be combined with the ionic liquid. Another equally effective extraction method is ionic liquid-based dynamic liquid-phase microextraction (dLPME). The method, for the extraction of phenothiazine and NSAID derivatives using the high density and viscosity of ILs, was developed by Cruz Vera [91,106]. The sample passes through the ionic liquid placed in a Pasteur pipette and the analytes are separated from the matrix. High viscosity is both an advantage and a limitation here as to perform the extraction it is necessary to reduce its value, therefore the addition of an organic solvent is also used.

##### Environmental Samples

In some publications, liquid-phase extraction procedures are very similar to DLLME procedures. One such procedure was proposed by Chatzimitakos et al. [64]. They used the potential of MIL [C_8_MAmm][FeCl] to determine many analytes (including ibuprofen and diclofenac). The authors defined their novel method as stirring-assisted drop breakup microextraction (SADBME). Thus, the IL dispersion element was defined as drop breakup. Although the authors focused on the method itself, they showed that the application of MIL allows for the simplicity of extraction. Due to magnetic property, the separation of the IL-phase was possible by applying an external magnetic field. A similarity to DLLME can also be seen in synergistic centrifugal assisted ionic liquid assisted microextraction (ILSVA-SME). Faster formation of microemulsion (dispersion) with the IL and the surfactant used is achieved by vortex-assisted extraction. The method, as in other cases, allows for better efficiency of results [107]. Song et al. [108] also proposed a similar method of extraction to the above-described DLLME. They used a solid IL to extract sulfonamides and then they dissolved them by microwave energy namely microwave-assisted liquid-liquid microextraction (MA-LLME-SIL), and then cooled them again and dissolved them in acetonitrile. However, as the authors highlighted, this is a different method to DLLME, because a solid IL was used and an organic solvent was not necessary. The dispersion step is present here by shaking the molten IL sample. Thus, they do not define it as DLLME. In search of the best results, only one type of liquid was used, and its appropriate volume, duration of use and microwave power were chosen. Inorganic salt was also added but, as opposed to other works, it was not NaCl, but Na_2_SO_4_.

Another approach to minimize the amount of organic solvents was the modification and adaptation of LPME methods to determine analytes. The first work described the use of ILs in three-phase hollow fiber supported liquid-phase microextraction (HF-LPME). The procedure was based on the transfer of analytes from the donor phase to the acceptor phase through a membrane with an IL placed in the pores. Due to the good solubility of sulfonamides in water, their transfer based on passive diffusion can be difficult. For this purpose, the combination of an IL with tri-n-octylphosphine oxide (TOPO) was used to create a semi-liquid membrane and facilitate the transfer of the analyte to the acceptor phase. During optimization, the IL was compared with n-undecane and dihexyl ether (DHE). The IL, as the most polar compound, allowed the highest efficiency [109]. The second work describing the modification of LPME was related to the addition of an IL to the acceptor phase (IL/n-octanol) in membrane bag-assisted-liquid-phase microextraction ((MBA)-LPME). The extraction set was prepared by the author (a detailed description can be found in the original publication) [110]. The effect of using an IL was an increase in efficiency. Among the tested ILs, the results improved only after using [C_6_MIM][TFP], which was explained by high hydrophobicity. Therefore, it should be noted that ILs, which are most relevant in DLLME, were not suitable for LPME-modification.

Extraction based on membranes was also proposed by Hanapi et al. [111] using an agarose membrane impregnated with an ionic liquid for electroconvulsive membrane extraction (IL-AF-µ-EME). An IL was used in both the membrane and the acceptor phase. During optimization, [C_6_MIM][PF_6_] and [C_8_MIM][PF_6_] were used. The cation with a hexyl substituent allowed for better performance. According to the authors, this is due to lower hydrophobicity, and therefore better solubility and conductivity. In addition to the type and the volume of the IL in the acceptor phase, other conditions (pH, ionic strength, mixing speed) were also optimized in experiments. The method was a fast process allowing for satisfactory validation parameters.

In one publication, ionic liquid-based immersed droplet microextraction (IL-IDME) was also used. Analytes, after transfer to IL droplets and in combination with a MeOH/ACN mixture, were analyzed by HPLC. Only one type of IL was used in the study, determining its optimal volume for analysis. During the optimization of other parameters, as in other papers, attention was paid to the effect of pH. Due to the determination of basic compounds, the samples were adjusted to an alkaline pH because analytes show greater affinity for ILs when in a non-ionized form [16].

#### 3.1.4. Aqueous Two-Phase System

##### Biological Samples

In addition to DLLME extraction, ILs are also used in aqueous two-phase systems (ATPs) [67,112]. These consist of two immiscible water phases enriched with two different substances which affect their physical and chemical properties. These could be polymers, inorganic salts or surfactants. Unfortunately, there are also disadvantages in this process, such as interaction with analytes. Therefore, in searching for new solutions, it was decided to use the potential of ILs here. As in DLLME, ILs retain analytes in one of the phases and help in their separation. The group of tested ILs also remains constant (an imidazolium cation with a different alkyl chain length and a hexafluorophosphate anion); however, the selection criteria change. What is most important is the ability to separate the two phases. Shao et al. [112] chose [C_4_MIM][PF_6_] for further experiments. They also tested an IL with a 1-ethyl-3-methylimidazolium ([C_2_MIM]) cation but then two phases were not formed. In longer alkyl chains, viscosity increased and analyte transfer decreased. In another publication with ATPs extraction, [C_6_MIM][PF_6_] was selected, the butyl substituent was too low in polarity and did not form separate phases with the SDS used, while an IL with C8 did not form a stable system. In addition, Yu et al. [67] checked how pH, IL volume, extraction time and the addition of inorganic salt affect ATPs. The results showed that all of the above factors are responsible for the total extraction effect. An increase in the volume of the IL caused an increase in the number of oil drops in the phase, the K_2_HPO_4_ used improved stability, while a change in pH and extraction time determined the final result of the efficiency. As we can also see, in this type of extraction the choice of IL is ambiguous and requires experimental testing. In addition, it is also important to choose a second substance that can determine the availability of analytes for the IL and the presence of two separate phases.

##### Environmental Samples

ATPs, which was described in the extraction of biological fluids, can also be used for environmental samples. Another form of ATPs, referred to as ionic liquid/salt aqueous two-phase flotation (IL-ATPF), was used to isolate chloramphenicol by Han et al. [113] (the solvent sublation apparatus was shown in the original work). The mechanism is based on the transfer of analytes into the IL droplets present in the upper surface phase of the system. As in other ATPs methods, the addition of inorganic salt was necessary and the best results were obtained through K_2_HPO_4_. The most appropriate IL was selected from three types: ([C_4_MIM][Cl], [C_8_MIM][Cl], [C_4_MIM][BF_4_]). [C_4_MIM][Cl] was used in further analyses, because of the lowest viscosity and surface tension, which is crucial when analytes must be absorbed by the IL droplets. The particular novelty was also the use of MIL in this type of extraction (1,1,3,3-tetramethylguanidine and 2,2,6,6-tetramethylpiperidine 1-oxyl free radical [TMG][TEMPO-OSO3]). The common effect of MIL is to obtain a rapid extraction by easily collecting the IL-analyte complex with the help of an external magnesium field. The formation of MILATP requires the addition of inorganic salt (as already mentioned in paragraph 3.1.1). In this experiment, after the optimization and interpretation of results, the best addition was K_3_PO_4_. The choice of temperature was also important, as too high could cause an increase in the solubility of the IL in water, so finally room temperature was chosen [114].

### 3.2. Sorbent-Based Extraction Procedures

#### 3.2.1. Solid-Phase Extraction

Solid-phase extraction (SPE) is a well-known sample pretreatment technique which ensures the simultaneous enrichment and purification of analytes [115]. In this technique, the compounds of interest and matrix interferences can be differentially desorbed from the SPE sorbent when water, an organic solvent or a mixture of organic solvent with water or salt solution are used as washing/eluting agents. It allows the analytes to be effectively extracted from the sample and the matrix interferences removed. In this extraction procedure, smaller amounts of organic solvent are required, and the risk of the formation of emulsions is decreased compared to LLE-based procedures. In effect, SPE is considered as a more environmentally-friendly method which is able to offer high analyte recoveries. Additionally, the SPE process is rapid and can be easily automated as an off-line SPE or on-line SPE system where direct coupling to chromatographic or electrophoretic separation systems is applied. In on-line SPE, a higher throughput and a more effective reduction of sample contamination or degradation can be obtained, while human exposure to potentially hazardous samples is decreased. On the other hand, the preconcentration and purification of the analytes in SPE may sometimes be ineffective because of the limited selectivity of conventional solid sorbents (e.g., modified silica-based sorbents). For this reason, new SPE materials are systematically developed and introduced to improve selectivity, including molecularly imprinted polymers (MIPs) as well as IL-based sorbents. In most investigations, ILs are immobilized by the covalent attachment of the imidazole group to the silica surface or polymeric support. These IL-based sorbents are considered to be interesting alternatives in SPE for different groups of pharmaceuticals from biological and environmental samples.

##### Biological Samples

**Figure 4:**
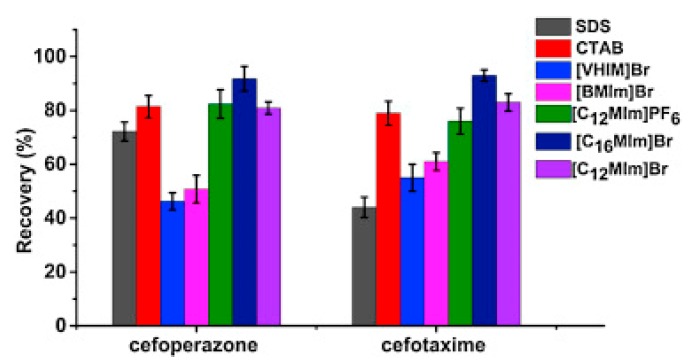
Comparison of the types of surfactants on the extraction efficiency of cefoperazone and cefotaxime. Figure adopted from the reference [121] with copyright permission.

Pang et al. [116] fabricated a polymer monolith column with 1-vinyl-3-hexylimidazolium bromide ([ViC_6_MIM][Br]) IL which was used for the on-line SPE isolation of betamethasone, norgestrel, halcinonide, beclomethasone dipropionate and testosterone propionate from human plasma. The developed SPE-HPLC-UV method offered the effective extraction of the analytes (93–105%), which allowed the target compounds to be quantified with LODs of 1–2 ng/mL. In a study by Liu et al. [117] a poly(ionic liquid-glycidylmethacrylate-coethyleneglycol dimethacrylate) (IL-GMA-co-EDMA) monolithic column with 1-vinyl-3-butylimidazolium chloride ([ViC_4_MIM][Cl]) was synthesized and applied as an SPE sorbent in the on-line SPE-HPLC-UV method for the determination of nifedipine, nitrendipine and felodipine in human plasma samples. The best extraction of the analytes and purification of the matrix sample was obtained when a methanol/water mixture was used as the eluting agent. It allowed the three antihypertensive drugs in human plasma samples to be determined with LODs of 2–3 ng/mL. Ferreira et al. [118] used 1-vinyl imidazole and 1,4-butane-sultane to create a silica-anchored IL-based material which was applied as a sorbent in an SPE system coupled online with HPLC-MS/MS for the quantification of the antibiotic ceftiofur in bovine milk samples. The extraction efficiency ranged from 70 to 130%, and the LOD was 0.1 µg/L. A sol–gel synthesis of three hybrid materials containing [C_4_MIM][PF_6_], [C_6_MIM][PF_6_] and [C_8_MIM][PF_6_], attached by covalent bonds, was published by da Silva and Mauro Lanças [119]. These IL-based hybrid materials were applied as the sorbents in off-line SPE for the isolation of five sulfonamides and trimethoprim from bovine milk samples. The results indicated that the extraction efficiency of the analytes systematically decreased when the alkyl chain of the IL increased from C4 to C8. This was probably caused by the reduction of the electron density and the steric hindrance from the methyl group on the three-substituent site of imidazole rings, which weakened the π–π interaction between the electron-rich benzene ring of the target compounds and the imidazole rings of the used ILs. The best efficiency was offered by an IL (C4)-based sorbent which was applied for the isolation and preconcentration of sulfonamide in bovine milk by the on-line SPE-HPLC-MS method. The LODs for the method developed were in the range of 1.5–2.25 μg/mL, with extraction recoveries from 74 to 93%. Yan et al. [120] developed modified dummy molecularly imprinted microspheres (DMIMs) based on [AC_2_MIM][Br] as the co-functional monomer and phenylephrine as the dummy template. These DMIMs were used as the SPE sorbent for the isolation of clenbuterol and clorprenaline from urine samples. The obtained results confirmed that they were able to more effectively extract the analytes and remove the matrix interferences than with other tested commercial sorbents such as HLB, PCX, C18 and SCX. For the DMIMs, the extraction efficiency ranged from 93.3 to 106%. The developed DMIMs-SPE-HPLC method allowed the analytes to be quantified with LODs of 0.19 and 0.070 µg/L for clorprenaline and clenbuterol, respectively. Ma and Row [1] synthesized a molecularly imprinted monolithic column using levofloxacin and ciprofloxacin as templates, 1-vinyl-3-ethylimidazolium bromide ([ViC_2_MIM][Br] as the functional monomer, and graphene oxide (GO) as the core material. When the efficiency of the IL-based imprinted monolithic column was tested as the SPE sorbent for the extraction of levofloxacin and ciprofloxacin from human urine, the best results were achieved using water as the washing agent, and a mixture of ethanol/acetic acid (7:3 *v/v*) for the elution of the analytes. The main advantages of the developed SPE protocol were the effective purification of the matrix sample, and the good extraction recovery of the analytes (89.5% and 92.5% for levofloxacin and ciprofloxacin, respectively). However, relatively low sensitivity of the developed SPE-HPLC-UV method was also observed (LODs from 0.06 to 0.27 μg/mL). Wu et al. [121] used an SPE procedure based on hemimicelles and admicelles (mixed hemimicelles) supported by an IL for the simultaneous extraction of five cephalosporins from biological samples. In this technique, the sorbent possesses adsorbed ionic surfactants on the surface of mineral oxides (e.g., SDS or IL) which enables two mechanisms to occur for the retention of the analytes—hydrophobic and electrostatic interactions. In effect, the extraction efficiency can be improved. The authors tested seven different surfactants, such as SDS, cetyltrimethylammonium bromide (CTAB), [ViC_6_MIM][Br], [C_4_MIM][Br], [C_12_MIM][PF_6_], [C_16_MIM][Br] and [C_12_MIM][Br]. The best recoveries were obtained for the long-chain IL [C_16_MIM][Br], which confirms data presented in (Figure 4).

The imidazolium-based IL with a longer alkyl side chain was probably able to strengthen the directionality of hydrogen bonds and van der Waals forces. In consequence, the interactions between the mixed hemimicelles and the hydrophobic regions of target compounds were more intensive and the efficiency increased. Taghvimi et al. [122] prepared mixed hemimicelle magnetic dispersive solid-phase extraction (MHMDSPE) based on carbon-coated magnetic nanoparticles and supported by the IL (IL-C/MNPs) for the extraction of tramadol from urine samples. In this study, MHMDSPE conditions were optimized, including both the selection of the adsorbent type and the solvent used as a desorbing agent. The results indicated that the IL-C/MNPs with [C_6_MIM][PF_6_] was more effective than that based on Fe_3_O_4_ NPs.

**Figure 5:**
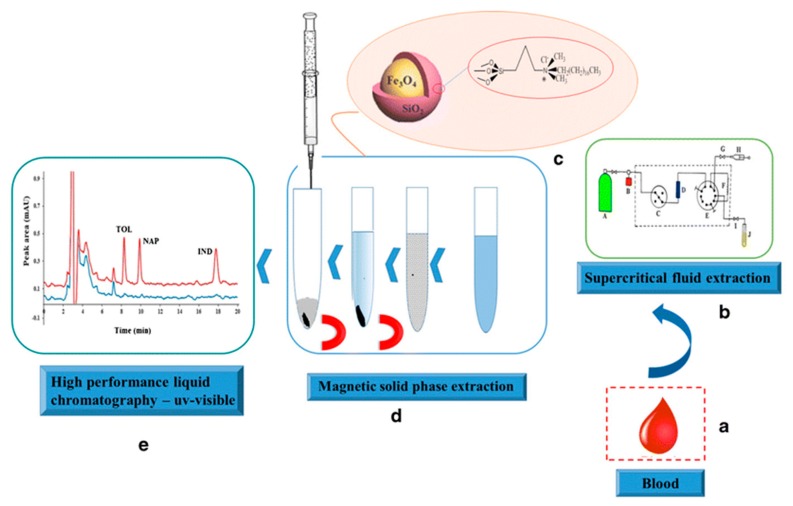
Schematic illustration of extraction procedure for tolmetin (TOL), indomethacin (IND) and naproxen (NAP) from blood samples. Figure adopted from the reference [126] with copyright permission.

This was probably related to the presence of carboxyl and hydroxyl groups on the surface of IL-C/MNPs, which improved the dispersion of the magnetic nano-adsorbent in the urine medium. In effect, stronger interactions between the analyte and the magnetic nano-adsorbent occurred, which improved the extraction efficiency. The best desorbing solvent was acetone, which allowed a recovery of 94% to be obtained. Yan et al. [123] prepared IL-modified magnetic polymer microspheres (ILMPM) based on Fe_3_O_4_ NPs and [C_4_MIM][PF_6_] used as a magnetic adsorbent of MDSPE for the determination of sulfamonomethoxine sodium and sulfachloropyrazine sodium in urine samples. The developed ILMPM-SPE sorbent provided a higher purification ability and extraction recovery of the tested analytes compared with magnetic polymers based on using 4-vinyl pyridine, methacrylic acid and acrylamide as monomers. A report was also published describing matrix solid-phase dispersion coupled with homogeneous ionic liquid microextraction (MSPE-HILME) applied for the extraction of sulfamerazine, sulfathiazole, sulfamethazine, sulfadoxine, sulfachloropyridazine, sulfaphenazole and sulfisoxazole from animal tissues [124]. In the study, three kinds of hydrophilic ILs, including [C_4_MIM][BF_4_], [C_6_MIM][BF_4_], and [C_8_MIM][BF_4_] were tested in MSPD and HILME simultaneously. The results confirmed that higher extraction recoveries of the analytes were obtained with the C_4_ IL than those observed with C_6_ and C_8_ ILs. This was related to the significant loss of C6 and C8 ILs in MSPD, which resulted in a small volume of the IL phase and low extraction yields of the target analytes. Compared to C_6_ and C_8_ ILs, the C_4_ IL possesses higher water miscibility and lower viscosity, which facilitates the transfer of target analytes from the sample matrix to the extraction solvent. In this study, this effect was predominant in respect to the extraction capacity of the IL, which often increases with the increase in the alkyl chain length of the IL [125]. Finally, water was selected as the elution solvent in MSPD because of the more effective extraction of sulfonamides, which are water-soluble polar compounds. In this procedure, the C4 IL was mixed with the dispersant and the sample before introduction to the MSPD column, and the IL phase was collected after HILM. When the MSPD-HILME method was coupled to HPLC-UV, the recoveries of the sulfonamides ranged from 85.4 to 118.0%. The LODs for the analytes were 4.3–13.4 g/kg. The application of magnetic core-shell nanoparticles (mag-NPs) of SiO_2_@Fe_3_O_4_ type, covalently modified with the IL (dimethyl octadecyl [3-(trimethoxysilyl propyl)]ammonium chloride) as the MSPE material for the extraction of tolmetin, indometacin and naproxen from blood samples was also described in the literature [126]. The synthesized mag-NPs were applied as the adsorbent in MSPE according to the protocol presented in Figure 5. 

The results of the study showed that the IL addition provided a more effective extraction of the NSAIDs probably due to an increase in both hydrophobic and π-π dipole or electrostatic interactions between the adsorbent surface and the analytes. On the other hand, the adsorption of the cationic molecules onto the sorbent was limited because of the repulsion interaction with the adsorbent surface. In consequence, a better purification of the sample was also achieved. The optimized MSPE was coupled to HPLC-UV and used alone or after supercritical fluid extraction (SFE) before HPLC separation. These protocols resulted in LODs between 0.1 and 0.3 μg/L for MSPE-HPLC and 0.2 to 0.3 mg/kg for SFE-MSPE-HPLC, respectively.

##### Environmental Samples

Fontanals et al. [127] synthesized and applied crosslinked polymer-supported imidazolium trifluoroacetate salt [MI^+^][CF3COO^−^] as the SPE sorbent for the extraction of salicylic acid, carbamazepine, nalidixic acid, flumequine, gemfibrozil and four NSAIDs from aqueous samples. In the study, the developed IL-sorbent was tested under weak anion exchange (WAX), strong anion exchange (SAX) and strong cation exchange (SCX) as well as reversed-phase (RP) SPE conditions. The best purification and extraction results of acidic pharmaceuticals from different water samples (ultrapure, tap, river water and effluent wastewater) were obtained when the IL-based SAX material was applied. In the next study, two new imidazolium supported IL phases possessing different anions such as [CF_3_(SO_3_)] and [BF_4_], were synthesized and applied as SPE-SAX sorbents for the isolation of acidic pharmaceuticals from water samples [128]. The obtained data indicated that [MI^+^][CF_3_(SO_3_)] and the previously developed [MI^+^][CF_3_COO^−^]-SAX sorbent gave comparable results, whereas [MI^+^][BF_4_] was not able to effectively extract and purify the acidic pharmaceuticals from environmental samples. On the other hand, the application of [MI^+^][CF_3_(SO_3_)] allowed only comparable efficiency to be obtained and calculated after using the commercially available Oasis MAX column, whereas [MI^+^][CF_3_COO^−^] was slightly more effective. Hydrophilic ciprofloxacin molecularly imprinted polymer material containing 1-allyl-3-vinylimidazole chloride ([AViMIM][Cl]) IL and 2-hydroxyethyl methacrylate as a bifunctional monomer was synthesized by Zhu and co-workers [129]. This MIP material was able to create strong hydrogen bonds, and electrostatic and π-π dipole interactions with ciprofloxacin in an aqueous solution. It offered excellent molecular recognition for common quinolone antibiotics (ciprofloxacin, levofloxacin and pefloxacin mesylate) in aqueous matrices as well as the selective isolation and separation of trace amounts of ciprofloxacin in real water, soil and pork samples, with recoveries of 87.3–102.5%.

#### 3.2.2. Solid-Phase Microextraction

Solid-phase microextraction (SPME), developed by Pawliszyn and his co-workers in the 1990s [130], is a fast, solvent less-extraction technique for the sampling, cleaning-up and pre-concentration of analytes, which also offers the introduction of the sample to chromatography in a single solvent-free step. The SPME sorbents can be applied in both the headspace mode and the immersion mode. The simplicity of the SPME technique and other advantages, such as high selectivity and effective purification, the relatively low cost of equipment and the possibility of automation, mean that SPME is a powerful tool for the extraction of a wide range of compounds from different matrices. Moreover, new sorbents for SPME based on ILs are also synthesized. Several publications have described the results of their application for improving the extraction efficiency of different groups of pharmaceuticals from biological and environmental samples.

##### Biological Samples

A paper can be found in the literature describing the use of SiO_2_@Fe_3_O_4_ functionalized with [C_4_MIM][PF_6_] IL for the microextraction of four β-blockers (propranolol, metoprolol, atenolol and alprenolol) from human plasma [131]. In the study, two types of hydrophobic ILs, ([C_4_MIM][PF_6_] and [C_8_MIM][PF_6_]), were tested. The results show that [C_4_MIM][PF_6_] offered an extraction efficiency of 75 to 91%, while for [C_8_MIM][PF_6_] these values were significantly lower (about 40%). This can be explained by the higher hydrophobicity of the long-chain IL, which leads to poor dispersion in the aqueous sample. Moreover, [C_8_MIM][PF_6_] cannot be completely recovered by MNP, which can additionally decrease the extraction efficiency [132]. In the developed sample preparation procedure, an effervescent powder composed of sodium dihydrogen phosphate and sodium bicarbonate was also applied for the enhancement of the interaction between the magnetic sorbent and the analytes. When this protocol was coupled with LC-MS/MS, the developed method for the analysis of β-blockers in human plasma was able to monitor the compounds of interest with LODs from 0.03 to 0.62 ng/mL.

##### Environmental Samples

Serrano et al. [133] published the synthesis of GO functionalized with covalently attached 1-butyl-3-aminopropyl imidazolium chloride IL to GO sheets, and its application as an adsorbent for the dispersive micro SPE of six β-blockers and four anabolic steroids from aqueous samples prior to HPLC separation. It was observed that hydrophobic attraction between the compounds and the GO-IL was the predominant adsorption mechanism of steroids, while for β-blockers, their interactions with the adsorbent were more complicated. For them, both hydrophobic and electrostatic interactions can occur as well as the existence of interactions of electron-donor-acceptor type, which are dependent on the pH used in the extraction process. These mechanisms were more intense on the GO-IL sorbent, which was confirmed by the recovery results for the analytes (87–98%), which were found to be significantly higher than those observed with GO alone and graphene. Yu et al. [65] prepared six neat crosslinked polymeric ionic liquid (PIL) sorbent coatings for the SPME of selected phenolics, insecticides and pharmaceuticals, including phenacetin, ketoprofen, fenoprofen calcium, diclofenac sodium and ibuprofen, from environmental water samples (tap water and lake water). These PIL sorbents were prepared using various IL monomers such as 1-vinylbenzyl-3-hexadecyl-imidazolium chloride ([ViBC_16_IM][Cl]), 1-vinylbenzyl-3-hexadecylimidazolium bis[(trifluoro-methyl)sulfonyl]imide ([ViBC_16_IM][Nf_2_T]), 1-vinyl-3-(2-hydroxyethyl)imidazolium bromide ([ViC_2_OHIM][Br]), 1-vinyl-3-(10-hydroxydecyl)imidazolium chloride ([ViC_10_OHIM][Cl]), 1-vinyl-3-(10-hydroxydecyl)imidazolium bis[(trifluoromethyl)sulfonyl]imide ([ViC_10_OHIM][Nf_2_T]), 1-vinyl-3-(9-carboxynonyl) imidazolium bromide ([ViC_9_COOHIM][Br]), and crosslinkers like 1,12-di(3-vinyl-benzylimidazolium) dodecane dichloride [(ViBIM)_2_C_12_]2[Cl]), and 1,12-di(3-vinylbenzyl imidazolium)dodecane dibis[(trifluoromethyl)sulfonyl]imide ([(ViBIM)_2_C_12_]2[Nf_2_T]). Next, they were tested in different experimental SPME conditions. The results indicated that all the developed PIL sorbent coatings were stable when the extraction was carried out under an acidic pH using various organic desorption solvents (e.g., methanol, acetonitrile, acetone). However, the best extraction results were obtained using the PIL-based sorbent coating polymerized from the IL monomer [VC_10_OHIM][Cl] and the IL crosslinker [(VBIM)_2_C_12_]2[Cl]. The extraction efficiencies of pharmaceutical drugs and phenolics were higher when the film thickness of the PIL-based sorbent coating increased from 23 µm to 89 µm, whereas these values were largely unaffected for insecticides. This analysis allowed LODs to be obtained ranging from 0.2 to 2 g/L for the target compounds. A report presenting the synthesis of four different crosslinked PIL-based sorbent coatings by UV polymerization onto nitinol wires was also published in the literature [134]. These PIL coatings possessed either vinylbenzyl or vinyl alkyl imidazolium-based (ViBCnIM- or ViCnIM-) IL monomers with different types of anions, and various dicationic IL crosslinkers. They were used in a direct-immersion solid-phase microextraction (DI-SPME) method for the extraction of a group of polar analytes and non-polar analytes (10 different compounds), including gemfibrozil and carbamazepine. Two studied fibers, such as the polymers PIL–1a from the IL monomer [ViBC_16_IM–Nf_2_T] and IL crosslinker [(ViBIM)_2_C_12_–2Nf_2_T], and PIL–2 based on the IL monomer [ViC_16_IM–Nf_2_T] and IL crosslinker [(ViIM)_2_C_12_–2Nf_2_T] were used for the extraction of the analytes from real tap and river water samples. The results confirmed that these PIL-based fibers offered reproducible and effective extraction of most of the tested analytes from real samples. The extraction can be carried out many times (up to 100 extraction-desorption steps), and at low pH values.

### 3.3. Stir Bar Sorptive Extraction

In recent years, a sample preparation procedure based on stir bar sorptive extraction (SBSE) has been developed for the extraction of compounds occurring in matrices at trace levels. It should be noted that the extraction mechanism and the benefits of SBSE are identical to SPME. However, the enrichment factor obtained in SBSE can be significantly higher compared to SPME (∼100 times). In SBSE, a glass tube with a magnetic core, coated with a layer of special polydimethylsiloxane (PDMS) tubing is applied to stir aqueous samples. After a certain time, the molecules captured on the bars can be desorbed either thermally for GC or into a solvent for LC. One drawback of SBSE is the low availability of different types of coatings. It should be noted that PDMS, mainly in SBSE, possesses a high affinity to extract non-polar compounds, while polar ones are poorly isolated. To overcome this limitation, new polymeric coatings are introduced, including poly (methyl methacrylate/ ethyleneglycol dimethacrylate) (PA-EG), and IL-based sorbents in order to improve the extraction efficiency of more polar compounds. Another problem of SBSE is the presence of the memory effect (carryover) during the desorption step using an organic solvent. According to the literature data, Talebpour et al. [135], in a comparative study, reported the application of a PA-EG polymeric phase and PDMS-coated stir bar supported by an IL for the extraction of carvedilol in human serum samples. In this investigation, [C_8_MIM][BF_4_] IL was tested as a modifier in the desorption solvent (methanol) for checking whether better extraction efficiency and the elimination of carryover can be obtained. The results confirmed that carvedilol has a better affinity for the PA-EG phase than for PDMS. Moreover, the addition of [C_8_MIM][BF_4_] at a concentration of 0.1 M to methanol significantly increased the recovery of carvedilol. Additionally, no carryover effect was observed, whereas it was detected when methanol was used without the IL (about 11% of the initial desorption step). Unfortunately, to the best of our knowledge, no report describing the use of IL-based sorbents for the SBSE extraction of pharmaceuticals from environmental samples has been published.

### 3.4. PASsive Sampling with Ionic Liquids

Extractions described so far can be classified as extractions with active sampling, because additional mechanisms, such as pressure and so on, are used for the flow of samples through the sorbent. However, the isolation of analytes is also possible in another way. Extraction using passive samplers can be used for the long-term monitoring of pharmaceuticals [136]. A significant difference in this method compared to procedures traditionally used in laboratories is the ability to estimate the time-weighted average concentration (TWAC) of analytes in ecosystems. Currently, the most popular Polar Organic Chemical Integrative Sampler (POCIS) techniques have been enriched with the new PASsive Sampling with Ionic Liquids (PASSIL) technique, developed by a team of scientists from the University of Gdansk. To carry this out, a dosimeter consisting of two disks and a membrane covered with the acceptor phase is necessary. Various ILs or their combinations with other sorbents are used as the acceptor, here.

**Figure 6:**
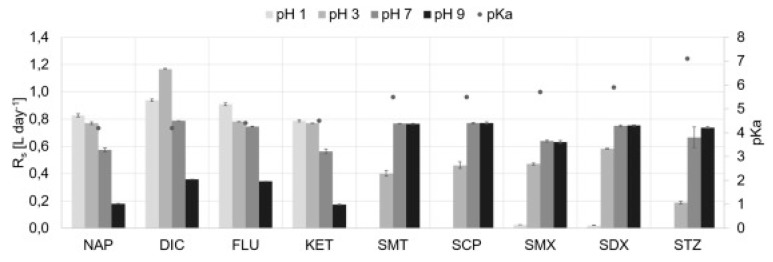
Dependency between sampling rate (Rs) values and the salinity of the donor solution for selected sulfonamides and NSAIDs (the pKa values of target compounds are specified by the black dots). Figure adopted from the reference [138] with copyright permission.

Caban et al. [137] compared the results of the isolation of analytes (diclofenac, carbamazepine and two sulfonamide antibiotics) using dosimeters in which the membrane was covered only with an IL or a combination of IL and colloidal silica obtained from C18 SPE extraction columns. In the experiment, they tested four ILs using not only the popular imidazolium cation but also the phosphonium cation ([C_6_MIM][Tf_2_N], [P_6,6,6,14_^+^][N(CN)_2_], [P_4,4,4,14_^+^][DDBS], and [P_2,4,4,4_^+^][(2O)_2_PO_2_]). The most important and desirable property of such sorbents was water insolubility. The content of the IL transferred into the donor phase was determined by testing the pH, conductivity and recovery of the phase. In order to select the best extraction conditions, the extraction efficiencies were calculated for all experiments. The results confirmed that when the IL alone was applied (independent of the type of IL), it did not improve the efficiency, and sometimes lower extraction parameters were calculated than those using traditional C18 sorbents (carbamazepine). In contrast, by using the combination of the IL and C18, the efficiency increased, the acceptor phase stability was improved and less IL consumption was possible. The developed method was used to extract analytes from saline water. The use of the matrix, which caused changes in the properties of the IL and analytes due to pH modifications, proved that the final result is a consequence of many components, not only choosing the right sorbent at the stage of method optimization. The same effects were observed in the study using a similar procedure to assess the effect of pH and salinity on the extraction efficiency of β-blockers, NSAIDs and sulfonamides using the PASSIL technique. It was interesting that the results for samples taken from the donor phase by a dosimeter with the same IL were different depending on the analyte. The extraction of β-blockers was impossible when an IL was used as the sorbent, even after changing the pH. In contrast, for NSAIDs and sulfonamides, the extraction efficiency improved after the appropriate pH modification (Figure 6).

The authors suggest that this situation results from the presence of β-blockers in a neutral or cationic form in the solution which cannot be adsorbed on the membrane surface to large [P_6,6,6,14_^+^]IL cations. In turn, the increase in salinity caused a decrease in the efficiency of analyte extraction due to their competition with the ions of salts present in saline water [138]. Męczykowska et al. [139] also assessed the effect of humic acids, temperature and mixing on the final extraction results of various pharmaceuticals using the PASSIL technique. The results indicated that each of these parameters can decide on the final results. Moreover, the importance was emphasized of polarity or hydrophobic properties as factors affecting these parameters.

## 4. Chromatographic Techniques

### 4.1. High Performance Liquid Chromatography

#### 4.1.1. IL Additives to the Mobile Phase

Liquid chromatography is the most commonly used technique for determining pharmaceuticals. Most of them are basic and their separation takes place in a reversed-phase using a silica-based column [140,141,142]. Unfortunately, this involves several serious problems during the analysis. The literature data indicate the main reason to be the presence of free silanol groups, which are negatively charged and can interact with positively charged basic analytes in an ion exchange reaction. Based on experimental research, it can be observed that this is often associated with problems with the resolution and shape of chromatographic peaks or a high retention factor. To prevent or minimize these deleterious effects, a mobile phase is used with additives for blocking free silanols [140]. The most popular additives are various types of amines, such as triethylamine (TEA), dimethyl-octylamine (DMOA) or buffers. The first researchers who noticed that ILs may also have suppressing properties against silanol groups were Kaliszan et al. [143]. In 2005 they published a paper in which they used an additive IL to the mobile phase in drug detection by thin layer chromatography (TLC) and reversed-phase liquid chromatography (RPLC) techniques. Since then, new publications have appeared systematically on similar topics (Table 3). However, considering the topic of ILs in drug determination, it should be highlighted that these works mainly focus on explaining the function of ILs in the suppression process and the drugs are less important as analytes. In addition, only a few works use biological [140,141,144,145,146] or environmental [142] samples as the matrices; in one, tablets were analyzed [147], but most often they were aqueous solutions [6,143,147,148,149,150,151,152,153,154].

**Figure 7:**
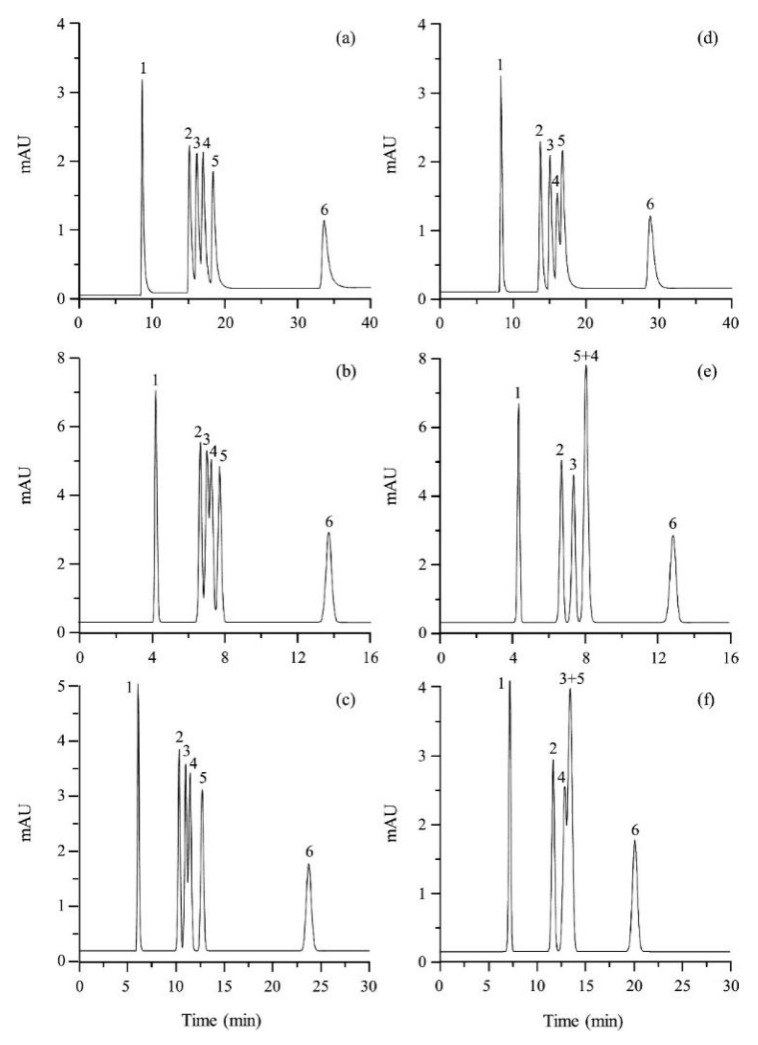
Simulated chromatograms for mixtures of the six TCAs using the C18 (**a**–**c**) and C8 (**d**–**f**) columns. Mobile phase composition: (**a**,**d**) 30% acetonitrile, (**b**,**e**) 30% acetonitrile/10 mM HMIM·Cl, and (**c** and **f**) 30% acetonitrile/10 mM HMIM·BF_4_. Peak identity: (1) doxepin, (2) imipramine, (3) nortryptiline, (4) maprotiline, (5) amitryptiline, and (6) clomipramine. Figure adopted from reference [147] with permission of the copyright holder.

Focusing on the addition of an IL to the mobile phase in LC, it should be noted that the interpretation of the results requires consideration of the influence of both the IL anion and cation. Although the use of the term IL suggests that one large molecule is responsible for the effect, it should be remembered that in the mobile phase the IL dissociates into both the cation and anion, so their combined effect determines the final results. It should also be highlighted that despite the involvement of other physical and chemical factors in the separation process, the largest changes in the chromatogram can be seen when using different kinds of ILs [142]. Their basic mechanism during pharmaceutical analysis is the reaction of IL cations with free silanol groups, the repulsion of IL cations with cations of basic analytes, as well as the reaction of IL anions with cations of analytes [149]. Depending on their type, the mechanism may be a little different than described. The choice of cation, as in the case of extraction (see the Section 3.2) focuses on the selection of an appropriate imidazolium cation with a different alkyl chain length. In one work, the analysis of the imidazolium cation with two methyl substituents was also carried out [143]. The effect of the cation was studied by Herrera et al. [142]. They performed analyses for ILs with the same anion [BF_4_] and different cations (Table 3). The results showed that an IL with a longer alkyl chain causes a decrease in the retention factor and an increase in efficiency. The effect of changing the retention time is similar in all analyses of basic analytes. The explanation for this effect may be an increase in hydrophobicity along with an increase in the length of the alkyl chain [144]. In turn, in a publication concerning the analysis of β-lactam antibiotics, an increase in the length of the alkyl chain caused an increase in retention. Han et al. [152] highlighted that a different effect may be the result of weak acidic properties and large analyte structures (the decrease in retention in other publications concerned basic analytes). The ester moiety of the antibiotic competed more strongly with the IL used for adsorption, and therefore despite the use of the long alkyl chain of the cation, retention increased. Ubeda-Torres et al. [149] also suggested that the size of the cation is more important than its nature. To study the effects of the IL anion, in other experiments, the same cation but a different anion was used during optimizing the IL selection. The number of anions tested is much greater than cations. The most commonly used are [PF_6_], [Cl] and [BF_4_] anions, but the less popular [CH_3_(SO_4_)], octylsulfate ([C_8_H_17_(SO_4_)]) and [Nf_2_T] have also been tested (Table 3). The analysis provided several important facts. First, the [PF_6_] anion showed very strong adsorption on the column and had a stronger effect on the parameters than the present cation. This is probably the result of its strongly chaotropic character [148]. The [BF_4_] anion is also a chaotropic ion, but with less adsorption than [PF_6_]. For this reason [BF_4_] more often qualified for further parts of the experiment. The next popular anion [Cl] belongs to strongly hydrated ions, and does not react with the analyte and the stationary phase; in its presence, the cation is mainly responsible for the mechanism [151]. Although the literature data provide information on the effects of the use of individual anions and cations, their choice is not obvious, not only because of the often antagonistic effect of ions. The use of an ionic liquid, which significantly reduces the retention time, is often associated with a poorer peak shape or resolution. In addition, too short a retention time for biological samples is not recommended because of the interference of analytes and background signals. In turn, improving the shape of the peak is possible at the expense of a higher retention factor. Figure 7 shows the change in retention after the use of two different ILs. Despite the shortening of the retention time by [C_6_MIM][BF_4_], an IL with [Cl] was chosen for the study due to better resolution. Therefore, the choice of IL is a kind of compromise, and the choice depends on many factors, including the type and number of analytes, and the type of matrices [147]. The results also show the influence of other factors on the final results. One of the most important modifications is the change in IL concentration. It was observed that a higher concentration leads to an improvement in the shape of the peak and reduces the retention time. However, this effect is more complex. First of all, the crucial factor here is whether the anion or cation has a stronger impact. For example, if the [PF_6_] anion is used, which has strong adsorption on the stationary phase, the retention time increases, but if the low affinity [Cl] anion and the long alkyl chain cation are used, the retention time decreases. However, it was noted that both with increasing and decreasing retention the effect occurs already at a very low IL concentration, and occurs until the column is completely filled with the IL. When column saturation occurs, a further increase in the IL concentration in the mobile phase has less of an effect on the results. The retention time is constant or the effect is the opposite to the current one. The mechanism of action of the aforementioned [PF_6_] in such a situation is explained by the reaction in the stationary phase until the column is saturated, and the reaction of this ion in the mobile phase after its saturation, and consequently, to a decrease in retention time [148,150]. The effect of pH on ILs was also analyzed, and it was found that at a lower pH the retention time is high because a larger number of [H^+^] ions react with the IL anions and the elution power decreases. This can be both a disadvantage and an advantage, because on the one hand, the separation improves, but on the other hand, the analysis time is too long [152]. In another study, the purpose of which was to assess the effect of buffers on separation parameters in the presence and absence of ILs, it was observed that the IL is mainly responsible for the retention time, while the buffers more strongly affect the final effect without the addition of the IL. However, it should be mentioned that the IL [C_6_MIM] with a strongly adsorbing cation was used in the experiment [7]. Another publication also suggests that retention is affected by the ratio of unprotonated to protonated silanols [6]. As mentioned above, ILs are an alternative to other mobile phase additives. For this reason, the results of studies with the addition of these compounds and with the addition of ILs are compared. ILs are better than other additives in all tests, but it must be highlighted that TEA the most popular compound, also gives good separation results [151]. In addition, the competitive advantage of ILs over other additives is the lack of effect on pH and, as shown in the literature, the involvement of both cations and anions in suppressing the silanol interaction and improving the results. In addition, the use of ILs is also possible in hydrophilic interaction liquid chromatography (HILIC). The increase in the stationary phase surface polarity obtained after the addition of an IL is responsible for improving the retention and efficiency parameters [146]. The verification of the positive effect of ILs was also presented in studies focusing more on the kind of columns used.

The analyses were performed on monolithic columns [144], popular C_8_ and C_18_ columns (Figure 7) [147] and six commercially available stationary phases [148]. In each analysis, the addition of ILs improved the results. It was also noted that the results depend on the production process of columns, which decide about the number of free silanol groups. Thus, the best results during the application of ILs in LC are obtained for columns for which the result was worse when using a traditional mobile phase without the addition of an IL [148]. As already mentioned, the application of ILs in LC focuses on the reaction mechanism, and drug determination is not essential here. Apart from a small number of analyses for real samples, the quality of the developed methods is not confirmed by determining the validation parameters. Therefore, the aspects of linearity, repeatability or reproducibility are ignored. To our knowledge, only one work has performed validation [147]. Moreover, only a few anions and cations have been tested in the analyses. There is no information on the effects of less common ILs. In addition, not all works compare the results obtained for ILs with other popular additives. The application of ILs also has several limitations. Although they extend the life of the column by protecting the surface of the stationary phase, conditioning is necessary for several hours to remove adsorbed IL ions and return the column to the starting position [143]. Due to the involvement of both anions and cations in the separation mechanism, other unknown interactions with their participation may occur. In addition, the choice of detector is an important issue during the application of ILs to the mobile phase. The following detectors can be used: FL, UV or *diode array detector* (DAD), but it should be remembered that ILs have a natural ability for ultraviolet absorption, which may affect the final results or prevent the selection of the optimal wavelength for analytes [148,155]. Moreover, the use of mass spectrometry is very problematic, here. However, despite the inconveniences described above, the popularity of ILs is constantly increasing and they are being used in subsequent experiments

#### 4.1.2. Ionic Liquid Stationary Phases

The application of ILs as an addition to mobile phases is not the only way to use them in liquid chromatography. In 2004, the first stationary phase appeared with ILs immobilized on the silica surface [156]. However, despite progress in this area, the use of IL stationary phases is much less popular than IL additives to mobile phases. Several-stage binding reactions are the first step of column preparation, producing as a final product modified IL-silica adsorbents, which finally coat the stationary phase (detailed reaction descriptions can be found in the original papers) [157,158,159]. Based on previous experience, several similarities can be observed to the previous section. First, the use of an IL stationary phase is the result of the incorrect peak shape, separation, efficiency and retention time obtained on traditional columns. As already mentioned, the same reasons concerned the use of ILs in the mobile phase. Secondly, research shows that both the cation and anion can be involved in the separation process. The imidazolium cation (single or multiple) is most commonly used to modify the stationary phase surface [160]. Furthermore, analytes were also separated on a column prepared using polymeric or chiral ILs [161,162]. There are many ways in the literature for obtaining IL-modified stationary phases based on various chemical reactions and substrates. However, their application is still not common. As mentioned, the number of publications is much smaller than the number of publications describing the suppression of free silanol groups by ILs present in the mobile phase.

**Figure 8:**
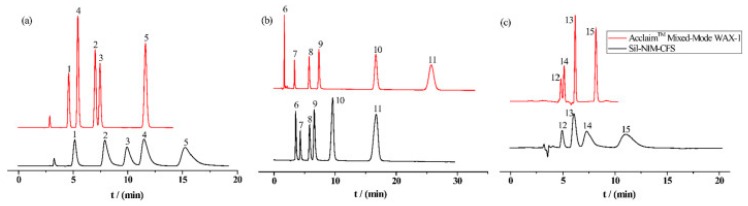
Separation of a mixture of nucleosides and nucleic bases, sulfonamides and inorganic anions on Sil-NIM-CFS and Acclaim™ Mixed-Mode WAX-1 columns. (1). uracil; (2). uridine; (3). cytosine; (4). adenine; (5). cytidine; (6). sulfanilamide; (7). sulfamethoxypyridazine; (8). sulfadiazine; (9). sulfathiazole; (10) sulfamethoxazole.; (11). sulfacetamide; (12). potassium bromate; (13). potassium bromide; (14). potassium iodate; (15). sodium iodide; (**a**) Mobile phase for Sil-NIM-CFS: ACN/10 mM ammonium formate (92:8, *v/v*); Mobile phase for Acclaim™ Mixed-Mode WAX-1 columns: ACN/10 mM ammonium formate (80:20, *v/v*); Ph = 5.6, flow rate: 0.6 mL/min, detection wavelength: 254 nm. (**b**) Mobile phase for Sil-NIM-CFS: ACN/H_2_O (50:50, *v/v*), flow rate: 0.8 mL/min; Mobile phase for Acclaim™ Mixed-Mode WAX-1 columns: ACN/H_2_O (60:40, *v/v*), flow rate: 1.0 mL/min; detection wavelength: 254 nm. (**c**) Mobile phase for Sil-NIM-CFS: ACN/5 mM Na_2_SO_4_ (5:95, *v/v*), pH = 4.28; Mobile phase for Acclaim™ Mixed-Mode WAX-1 columns: ACN/50 mM Na_3_PO_4_ (50:50, *v/v*), Ph = 6.0; flow rate: 0.6 mL/min; detection wavelength: 210 nm; Injection volume: 40 μL, column temperature: 25 °C. Figure adopted with permission from [165].

This review focuses primarily on the determination of pharmaceuticals in biological and environmental samples, so it should be strongly highlighted here that the number of publications related to the determination of such analytes on IL columns by LC is negligible. Two such articles were published by Rahim et al. [163,164]. They prepared a stationary phase based on β-cyclodextrin and 3-benzylimidazolium tosylate as ILs for the enantioseparation of β-blockers and NSAIDs. The results confirmed an enhanced enantioseparation and better enantioresolution on the novelty stationary phase. Another publication in accordance with the criteria adopted in the review was published in 2019 by Xian et al. [165]. The stationary phase was prepared with photo-initiated thiol-ene click chemistry using the imidazolium cation and anion [Nf_2_T]. Then, on the prepared column, the sulfonamides were separated by mixed-mode HPLC (MHPLC). The results confirmed good performance and separation selectivity, and additional research on commercial columns proved that the IL is responsible for a shorter separation time (Figure 8). Although the determination of drugs using IL column modifiers is very rare, their application in the determination of vitamins [166], flavonoids [167], amino acids [168] and many other compounds shows that perhaps in subsequent years these methods will be extended also for such analytes.

### 4.2. Other Chromatographic Techniques

#### 4.2.1. Gas Chromatography

In gas chromatography (GC), ILs have found use as stationary phases. This is due to the fact that they have unique properties, such as a wide liquid phase range, low volatility (negligible vapour pressure), high viscosity, good thermal stability and variable polarities, which make them suitable for that purpose [169]. Research into using molten salts in GC started in the 1950s and now IL-based columns are used in the analysis of complex samples [29,170]. A characteristic property of ILs is that they display unusual dual nature retention behavior, separating both non-polar and polar compounds. On the one hand, ILs exhibit a similar behavior to polar stationary phases such as polyethylene glycol or cyanopropyl-substituted polysiloxanes due to their ability to display a high dipolar interaction and hydrogen bonding. On the other hand, they are able to retain non-polar solutes (i.e., alkanes and alkenes), similarly to the low polarity stationary phases such as phenyl substituted dimethyl polysiloxanes [171]. Another important feature of ILs is that varying the cation or anion might significantly affect their physical and chemical properties [169]. For example, imidazolium IL columns using the [Nf_2_T] anion were the most efficient [29]. ILs formed by less coordinating or nucleophilic anions, such as [Nf_2_T], tend to be more stable compared to those containing halide salts; in fact, the latter are characterized by a nucleophilic nature, and hence it is possible for them to undergo SN1 or SN2 reactions with the alkyl substituents of the cation. Phosphonium-based ILs, synthesized with a large alkyl chain substituent, have shown outstanding thermal stability; in particular, a dicationic phosphonium IL, namely the dicationic 1,12-di(tripropyl-phosphonium)dodecane bis(trifluoromethylsulfonyl)imide, was synthesized possessing a thermal stability of 425 °C [172]. In addition, it should be noted that dicationic and tricationic ILs exhibit significantly higher thermal stability compared to monocationic-based ILs [173].

At present, IL-coated capillary GC columns are commercially available under the trade name SLB-IL (Supelco–Sigma-Aldrich, Darmstadt, Germany) (Table 4). These columns are characterized by a different polarity and can work at temperatures passing 200 °C and approaching 300 °C [174]. Studies on the synthesis and properties of new IL-stationary phases are still continuing. Yu et al. [175] analyzed the application of a new triptycene-based amphiphilic material (TP-2IL) as the stationary phase for GC separations. In research, they showed that this column exhibited good performance for analytes from an apolar to a polar nature. Particularly, it has an outstanding capability for resolving critical pairs of anilines and phenols with good peak shapes, and shows distinct advantages over the typical conventional stationary phases. IL columns find many uses in the analysis of flavors and fragrances [176,177], fatty acids [178,179,180,181,182] and petrochemicals [183,184]. González Peredes et al. [185] evaluated different IL columns for the separation of chlorobenzenes and developed an analytical methodology based on the use of the IL stationary phase SLB-IL82 in GC with a microelectron capture detector for the determination of chlorobenzenes in soil samples.

Do et al. [186] showed that the profiling of all 136 PCDD/Fs is greatly facilitated by using IL columns or combinations including such columns. Boczkaj et al. [187] tested three capillary columns (HP-5Ms, DB-624, SLB-IL 111) in the analysis of oxygenated volatile organic compounds (O-VOCs) in postoxidative effluents from the production of petroleum asphalt. Among the capillary columns investigated, a very polar column, SLB-IL 111, with an ionic liquid as the stationary phase was found to be superior for the separation of O-VOCs, as it has a high selectivity towards n-alkanes and oxygenated volatile organic compounds.

So far, IL stationary phases have not been widely applied in the field of bioanalysis [188]. Destaillats et al. [189] applied an IL-coated SLB-IL 111 column to identify the occurrence of petroselinic acid in human skin, hair and nails. They confirmed that this column can be used to obtain a baseline resolution between petroselinic acid and cis-8 18:1 acid methyl esters.

The use of IL-based stationary phases has extended to multidimensional gas chromatography (GCxGC), ensuring future applications. For example, Zapadlo et al. [190] investigated the use of GCxGC–TOFMS with highly polar IL-based columns for the analysis of polychlorobiphenyls (PCBs). They used a non-polar/ionic liquid column series consisting of poly (50%-n-octyl-50%-methyl) siloxane (SPB-Octyl) and the ionic liquid SLB-IL59 in the first and second dimension, respectively. As a result, a total of 196 out of 209 PCBs congeners were resolved and all dioxin-like congeners were separated with no interferences from any PCB congener.

ILs have found another significant application as a solvent in headspace gas chromatography (HS-GC), ILs are ideal solvents for HS-GC, a more sensitive method of analysis compared to direct injection. HS-GC avoids direct liquid or solid probing and greatly decreases matrix interference [29]. ILs are excellent solvents and are now used for the analysis of residual solvents in a variety of pharmaceutical products. The detection and quantitation of residual solvents/impurities in drug substances or drug products is an important measure for pharmaceutical quality assurance/quality control, because the residual solvents/impurities that were not totally removed by practical manufacturing techniques always have a potential risk to human health from toxicity. Fink et al. [191] developed a rapid, accurate, IL-based HS-GC method for the determination of water in active pharmaceutical ingredients. The HS-GC method used an IL-based capillary GC column to increase the sensitivity and ruggedness of this method. ILs are also utilized as a headspace solvent. Studies have shown that the sensitivity of the HSGC method is 100 times greater than that of volumetric Karl Fischer titration (KFT) (which is the commonly used technique to determine water content), allowing very small sample sizes (e.g., 4 mg) to be accurately and reproducibly analyzed. In comparison, a typical sample size of 500–1000 mg is used in KFT. Liu and Jiang [192] applied [C_4_MIM][BF_4_] as the matrix medium in the analysis of six solvents utilized in the synthesis of Adefovir Dipivoxil: acetonitrile, dichloromethane, N-methyl-2-pyrrolidone ([NMPyrr]), toluene, dimethylformamide (DMF), n-butyl ether. The developed method proved accurate and linear (with *R* ≥ 0.9993). All the RSDs were lower than 10%. Moreover, the comparison of [C_4_MIM][BF_4_] with DMSO as a matrix medium of headspace GC was also carried out in this study. In this research, it was indicated that DMSO, with its boiling point at 189 °C, has a higher vapor pressure, and the chromatographic peak of the DMSO matrix, with a much higher intensity, always occupies a wider baseline or interferes in the detection of analytes, especially at a higher equilibrium temperature. The impurities and the decomposed products of DMSO at a high equilibration temperature also became interfering substances for the detection of residual solvents.

A great analytical challenge for the pharmaceutical industry is the trace-level analysis of genotoxic impurities (GTIs) in drug substances. Ho et al. [193] used ILs (six compounds: ([C_4_MPyrr][B(CN)4]), [C_4_MIM][BF_4_], [C_4_MMIM][BF_4_], [C_4_MIM][Nf_2_T], [C_4_MMIM][Nf_2_T] and ([P_6,6,6,14_^+^][Nf_2_T])) as a new class of diluents for the analysis of two classes of genotoxic impurities (GTIs), namely, alkyl/aryl halides and nitro-aromatics, in small molecule drug substances by headspace gas chromatography coupled with electron capture detection (ECD) without the need for analyte derivatization. The low volatility and high thermal stability of ILs enables these compounds to be used at high headspace oven temperatures with the minimum chromatographic background. Studies have shown that increasing the headspace oven temperatures resulted in varying responses for alkyl/aryl halides and significant enhancements in the responses for all nitroaromatic GTIs. Furthermore, ILs with a conventional high-boiling organic diluent—DMSO, were compared. The chromatographic backgrounds from ILs are significantly lower than the backgrounds from DMSO. The LODs of all analytes obtained using the IL diluents were superior (5 to 500 ppb) to those obtained from pure DMSO. Research on organic solvent residues in drugs was also conducted by Ni et al. [194]. The main focus of this study was to investigate the relationship between analytes (organic solvents) and the matrix medium (ILs) by HS-GC in order to provide guidance in choosing a suitable matrix medium and next to determine the organic residual solvents in ketoconanzole to choose a suitable IL during the process of HS-GC. In research, [C_4_MIM][PF_6_] was chosen as the best headspace solvent, because an excellent separation of ethanol, dichloromethane, ethyl acetate, butyl alcohol, pyridine, DMF and DMSO was achieved. The evaluation of ILs for the analysis of residual solvents in pharmaceutical matrices was the subject of research by Laus et al. [195]. The authors chose ([C_4_MIM][DMP]) as the most suitable ionic liquid as solvent for the HS-GC analysis of solvents with very low vapor pressure, such as dimethylsulfoxide, *N*-methylpyrrolidone, sulfolane, tetralin, and ethylene glycol which can be found in pharmaceutical products. The limit of quantification (LOQ) of this method was from 59 (tetralin) to 113 µg/g (ethylene glycol), the accuracy was in the range of 96.6–103.7, and it showed high linearity in the tested range of 0.9890–0.9984. The developed method was applied for the detection of traces of sulfolane in a real sample of tablets containing the drug cefpodoxime proxetil. A trace of sulfolane was detected (estimated at 2 µg/g with respect to the tablet mass), which is safely below the regulatory limit of 160 µg/g. To the best of our knowledge, there are only a few reports on the use of ILs as stationary phases for GC separations in the analysis of environmental samples. One of them is the investigation performed by Reyes-Contreras et al. [171], who examined the suitability of ILs as stationary phases for GC–MS, and their application for the determination of nitrosamines and caffeine metabolites in wastewater samples. Studies have shown that the SLB-IL111 column enabled the baseline separation and quantification of 7 nitrosamines in a shorter analysis time compared with the commonly used cyanopropylphenyl polysiloxane. Furthermore, the SLB-IL59 column provided the elution of all the caffeine metabolites analyzed with the highest peak symmetry and an appropriate analysis time. Contaminants in wastewaters were also the subject of research by Domínguez et al. [169]. In this work, the application of IL stationary phases for the determination of benzothiazoles and benzotriazoles was examined. Among the IL columns evaluated, SLB-IL59 provided the total elution of all the target analytes with the highest peak symmetry and the lowest analysis time. Moreover, the lower stationary phase bleeding enabled positive identification and quantification.

In view of the properties of ILs, such as high thermal stability, high viscosity, and tunable selectivity through the modification of their chemical structure, their use, in particular as stationary phases in GC, will increase. New IL stationary phase chemistries that provide unique selectivity towards target analytes are needed to improve the separation performance and versatility of multidimensional GC [170].

#### 4.2.2. Thin-Layer Chromatography

ILs are used in Thin-layer Chromatography (TLC) as stationary phase modifiers, especially in the separation of basic drug compounds. The separation of drug compounds is carried out normally with the use of silica-based stationary phases; however, it is often impossible because of the effect of free silanols on their chromatographic retention [196]. In order to remove this undesirable phenomenon, methods are used such as protonation, the addition of traditional amino quenchers, and changes in the mobile phase composition in order to increase ionic strength. The solution to this problem may be the application of ILs as silanol suppressing agents. The first study on improving separation in TLC by using ILs as mobile phase modifiers was presented by Kaliszan et al. [197]. The efficacy of using ILs as stationary phase modifiers in TLC has also been confirmed in research by Marszałł et al. [198]. The aim of this study was the application of imidazolium-based ILs to reduce the deleterious effects of free silanols on the LC separation of naphazoline nitrate. The authors used [C_2_MIM][BF_4_] and [C_6_MIM][BF_4_] as modifiers of the mobile phase. The results showed that ILs with short alkyl-chain lengths are efficient suppressors of free silanols, which are considered to be responsible for the troublesome and irreproducible chromatographic determinations of basic compounds. In the next study, Kaliszan et al. [199] also reported that ILs of the imidazolium tetrafluoroborate class when added to mobile phases blocked silanols and provided excellent TLC separations of strongly basic drugs which were otherwise not eluted, even with neat acetonitrile as the mobile phase. The ILs used by Marszałł et al. [198] as mobile phase modifiers were also tested in the studies reported by Mieszkowski et al. [196,200]. In the first study, 1-alkyl-3-methylimidazolium-based ILs (tetrafluoroborate [C_2_MIM][BF_4_], L-(+)-lactate [C_2_MIM][LAC] and ethyl sulfate [C_2_MIM][ETOSO_3_]) were used as the mobile phase [196]. The subject of the research was the development of a new HPTLC method for the determination of perazine in oral tablets, and a comparative study between these three different ILs with the same cation but different counterions as additives to the mobile phase. In effect, among the selected ILs, the optimum distribution parameters, such as shape and quality of spots, high precision, and accuracy in qualitative and quantitative determination, characterize the system, with [C_2_MIM][BF_4_] as the mobile phase modifier. Summarizing this study, it can be concluded that [C_2_MIM][BF_4_] is a valuable and efficient suppressor of free silanols, which are responsible for unwanted interactions of chromatographic stationary phases in the determination of the above compounds. In the second study, the authors compared two TLC methods for the determination of haloperidol in oral drops—the pharmacopeia method (European Pharmacopeia 7.0) and an alternative with IL modifiers of the mobile phase. The addition of [C_2_MIM][BF_4_] to the mobile phase gave similar separation and quantitative results with no peak tailing compared to the mobile phase suggested by the European Pharmacopeia 7.0 [200]. Besides the silanol-suppressing potency of [C_2_MIM][BF_4_], a lack of interaction and interference with UV densitometric detection was observed. Research on the use of ILs in TLC was also conducted by Lu et al. [201], who used ILs as mobile and stationary phases of TLC to analyze berberine hydrochloride, tetrahydropalmatine and related Chinese patent medicine. In this study, the shape and value of target spots together with the developing duration were compared regarding four mobile phases which were a combination of the ILs ([C_4_MIM][OH]), [C_4_MIM][BF_4_], [C_4_MIM][Br], [C_4_MIM][PF_6_]) and methanol. Moreover, these IL mobile phases were compared with two traditional developing reagents, *n*-hexane-chloroform-methanol and *n*-butanol-acetic acid-water. As a result, it was found that [C_4_MIM][OH]-methanol has a simpler composition and is more suitable for the simultaneous analysis of two target constituents in a plate. Besides any extra pH additives, the shape of spots was ideal and no tailing occured. [C_4_MIM][OH] was also used as the stationary phase, which was synthesized based on silica gel. The quantitative method for this kind of IL stationary phase showed a good correlation coefficient (*R^2^* = 0.9971–0.9976), good repeatability (%RSDs of berberine hydrochloride and tetrahydropalmatine were 0.88% and 0.79%, respectively) and method accuracy in terms of 95.91–104.85% (berberine hydrochloride) and 96.02–102.18% (tetrahydropalmatine). Research into the application of ILs as mobile phases in TLC was published by Tuzimski and Petruczynik [202]. The aim of the study was the separation of ten components of a mixture of isoquinoline alkaloids: allocryptopine, berberine, boldine, chelidonine, papaverine, emetine, columbamine, magnoflorine, palmatine and coptisine, using a 2D-TLC (two-dimensional TLC) method. The first dimension used an aqueous mobile phase (RP) (80% methanol–water–0.05 M/L−diethylamine), and in the second dimension a normal phase (NP) (75% methanol, 24.75% ethyl methyl ketone–0.25% IL [C_4_MIM][BF_4_]). The addition of ILs to conventional mobile phases caused a decrease in zone broadening and improved the chromatographic resolution. As shown in the results of the experiments, very symmetrical spots and peaks and high system efficiency were obtained. In conclusion, the authors proposed that mobile phase systems containing ionic liquids can be applied to the separation of isoquinoline alkaloids in other natural samples. The use of ILs as stationary phase modifiers can be an effective and more “green” alternative to classical mobile phases such as amines.

#### 4.2.3. Supercritical Fluid Chromatography

Among the numerous applications of ILs, they can also be used in solvent systems composed of ILs and supercritical fluids with an emphasis on supercritical carbon dioxide (scCO_2_). The specificity of IL–supercritical fluid biphasic systems follows from the availability of several mechanisms for tuning the solvent properties of such systems—apart from the wide selection of IL cations and IL anions to tailor the IL properties, the operating temperature and pressure are also available as variables to adjust the density and the solvent power of the supercritical fluid phase [203]. In an ILs-scCO_2_ system the product recovery process is based on the principle that scCO2 is soluble in ILs, but ILs are not soluble in scCO_2_. Since most organic compounds are soluble in scCO_2_, with the high solubility of scCO_2_ in ILs, these products are transferred from the IL to the supercritical phase [204]. Ji et al. [99] applied the IL [C_8_MIM][PF_6_] and methanol as the extraction and dispersion solvents in a method for the determination of four NSAIDs—nabumetone, ibuprofen, naproxen and diclofenac—in tap water and drinks. The method was based on ultrasound-assisted ionic liquid dispersive liquid–liquid microextraction (US-ILDLLME) followed by ultra-high performance supercritical fluid chromatography (UHPSFC) coupled to a photo-diode array detector (PDA). The developed method showed rapid separation (2.1 min), good recoveries (81.37–107.47%) and enrichment factors (126–132). The LODs for the analytes were from 0.62 (naproxen) to 7.69 (ibuprofen) ng/mL. This developed procedure was applied to real water samples, tap water, soda, lemon juice and green tea drink. In soda drink, ibuprofen was detected with detection levels of 16.43 ng/mL.

**Figure 9:**
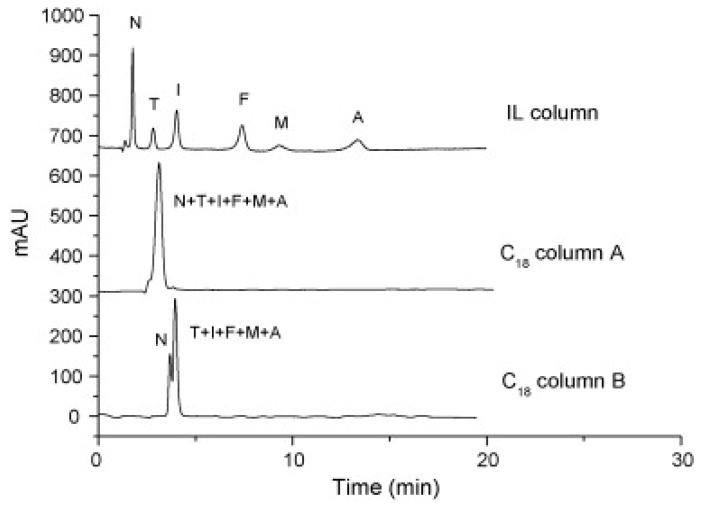
Separation of acidic, basic, and neutral compounds via SFC using the IL-modified column and a commercial C_18_ column. Figure adopted with copyright permission from [207].

Because SFC can be performed with both polar and nonpolar stationary phases, columns that are marketed for HPLC can be used in SFC [205]. The application of immobilized ionic liquids (IILs) as a class of stationary phases for packed column SFC was studied by Smuts et al. [206]. The authors studied the cation and anion effect. The research was conducted on different IILs: tripropylphosphonium, tributylphosphonium, methylimidazolium, benzylimidazolium, triphenyl-phosphonium and 4,4′-bipyridyl while keeping the counteranion constant, and an immobilized tributylphosphonium with five different anions: acetate, trifluoroacetate (TFA), [Cl], perchlorate and [Nf_2_T]. The best stationary phase in terms of low retention and good separation efficiency was the IIL tributylphosphonium with the TFA counter anion. Furthermore, the acetate anion exhibited the worst retention time and repeatability, and took the longest to reach baseline stability. [Nf_2_T]^-^ displayed poor efficiency in separations for tributylphosphonium-based stationary phases. Chou et al. [207] used covalently bonded 1-octyl-3-propylimidazolium chloride on a silica gel column for the simultaneous separation of acidic, basic and neutral compounds (fenoprofen, ibuprofen, acetaminophen, metoprolol, naphthalene and testosterone) using carbon dioxide subcritical/supercritical fluid chromatography. The data indicated that the IL-modified column, in terms of resolution, was clearly superior to commercial C18 columns. Also, the simultaneous separation of acidic, basic and neutral compounds via SFC was successful with a co-solvent content of 20% MeOH, a pressure of 110 bar, and a column temperature of 35 °C (Figure 9).

In conclusion, it should be stated that ILs seem to be good replacements for volatile organic solvents, and the development of new applications utilizing ILs will increase. However, the high cost of ILs and lack of complete data on e.g., toxicity should be noted.

## 5. Electromigration Techniques

### 5.1. Capillary Electrophoresis

CE, which belongs to electromigration separation techniques, possesses many advantages, such as low sample and reagent consumption, high efficiency, simplicity, short analysis time, automation and inexpensive cost of capillaries in comparison to HPLC columns. CE separations are also extremely effective and allow substances with similar structures to be separated. These advantages mean that this technique has become an interesting alternative analytical tool to other chromatographic methods. Generally, CE analysis is carried out on fused-silica capillaries with silanol groups on the inner surface which are normally negatively charged. This results in the formation of an electroosmotic flow (EOF) that moves compounds toward the cathode when a voltage is applied across a tube filled with an electrolyte solution. Contrary to this effect, electrophoretic mobility exists, which moves a molecule to its opposite electrode. Each ion possesses a specific electrophoretic mobility resulting in a charge-to-mass ratio. However, the effect of EOF is generally predominant in respect to electrophoretic mobility, causing all the molecules to be moved at different speeds toward the cathode. A higher speed can be observed for cations, and neutral analytes take slightly longer to migrate, while negatively charged compounds take the longest to move because of their conflicting electrophoretic mobility. The fact that, simultaneously, both EOF and electrophoretic mobility occur, working on anions in opposite directions during electrophoretic separation, allows greater resolution to be obtained. The main parameters which can affect EOF mobility are the dielectric constant, the zeta potential value and the viscosity of the buffer. The values of these parameters can be regulated by the modification of the background electrolyte (BGE) and/or using different buffer additives, as well as when the physicochemical properties of the wall of the capillary are changed. ILs are considered as good EOF modifiers because of their good electrical conductivity and they are slightly more viscous than organic solvents. In effect, low IL concentrations can be enough for a significant improvement in the electrophoretic separation. According to the literature data, ILs have been applied as the BGE, as additives to the BGE and/or as covalent coating reagents of the capillary. However, taking into account the costs of these modifications, ILs were mainly used as electrolytes or additives to electrolytes to modify the capillary wall. It should be highlighted that both cations and anions of ILs may change the migration behavior of analytes, although the activity of IL cations have a major impact on the resolution in CE. The IL cations, by the modification of the ionic strength of the BGE, can change the EOF, which influences the migration times of the analytes and may improve separation efficiency. Other activity is related to the adsorption of IL cations on the capillary inner surface, which can reduce or even reverse the EOF as well as possibly correcting the peak tailing of some basic enantiomers. Both mechanisms mentioned above allow a significantly better resolution of analytes to be obtained [199,208].

For example, Qin et al. [209] used a 1-methylimidazolium-based IL for covalent bonding of the fused-silica capillary surface wall for reversing the EOF during the development of a CE-MS method for the determination of sildenafil (SL) and its metabolite UK-103,320 (UK) in human serum samples. The most effective separation was obtained with a BGE containing 10 mM of acetic acid (pH 4.5) and with a voltage of 25 kV. The sensitivity and resolution were significantly improved because this approach allowed the elimination of the adsorption of the compounds on the IL-coated capillary wall, which occurred on the bare fused-silica capillary wall. In effect, the analytes passed through the IL-coated capillary with a recovery of 98% and 100% for SL and UK, respectively. Moreover, the resolution between SL and UK was enhanced because of the modification of the EOF. The analytes were separated within 14 min with LODs of 14 and 17 ng/mL for SL and UK, respectively. El-Hady et al. [210] proposed a CE-UV method for the simultaneous determination of four anticancer drugs in human plasma and urine based on [C_4_MIM][Br] as a component of the BGE. During the study, the parameters of CE separation were optimized. The best results were obtained when the analysis was carried out on a BGE containing a 12.5 mmol/L phosphate buffer at pH 7.4 and 0.1 μmol/L of [C_4_MIM][Br] (IL), and 20 kV applied voltage. This approach allowed sensitivity to be increased 600 times over that observed in CE performed without the IL. The developed CE-UV method for the quantification of methotrexate, vinblastine, chlorambucil and dacarbazine in human plasma and urine allowed the analytes to be monitored with the LODs in the range of 0.01 to 0.05 μg/mL.

It should also be noted that excellent separation is particularly required for the analysis of racemic mixtures, including various groups of pharmaceuticals the enantiomers of which can possess significant different pharmacokinetic and pharmacodynamic properties and side effect profiles. The qualitative and quantitative analysis of the compounds in biological and environmental samples is necessary for better understanding the mechanism of their activity in live organisms and their influence on the environment. This issue was a predominant topic of many papers published in recent years in world scientific literature. In those studies, both achiral ILs and chiral ILs (CILs) were applied in combination with various types of chiral selectors (CS) like cyclodextrins (CDs) or their derivatives, antibiotics, polysaccharides or surfactants for the chiral separation of different pharmaceuticals. Typical achiral ILs applied in CE enantioseparation were tetraalkylammonium ILs, alkylimidazolium ILs and alkylpyridinium ILs with inorganic anions such as [OH], [Cl], [Br], [BF_4_] and [PF_6_]. Among them, tetraalkylammonium-based ILs are considered as more effective because of their relatively more hydrophilic character, which decreases the likelihood of entering the hydrophobic cavity of the CS. Moreover, their relatively lower conductivity and UV transparency in the wavelength ranges applied for enantiomer detection allow them to be used in higher concentration levels. These data are in accordance with the study reported by Huang et al. [211] who tested alkylpyridinium, tetraalkylammonium and alkylimidazolium-based ILs along with β-CDs for the chiral separation of five β-agonists. The results confirmed that tetraalkylammonium-based ILs were more effective because they could be used at much higher levels than the other tested ILs. Poor resolution was achieved when the long-chain IL, [C_8_MPyrr][PF_6_], was used as the BGE modifier. Moreover, the presence of ILs was required for the full enantioseparation of salbutamol, cimaterol and formoterol, which were not resolved using the BGE containing only β-CD as the CS.

Jiang et al. [212] used [C_2_MIM][BF_4_] for the coating of a silica capillary during the enantioseparation of ibuprofen, fenoprofen, naproxen and ketoprofen. It enabled the EOF to be modified, which provided the effective resolution of the enantiomers. The tested IL not only affected the EOF but also acted as a discriminator. Moreover, the interaction between hydrogen at the C–2 carbon of the IL and the acid drugs played an important role in the separation. The same type of IL was selected for the enantiorecognition of nine tricyclic antidepressants in the study reported by Tsai et al. [213]. The optimal simultaneous separation of all the tested pairs of enantiomers was achieved with 50 mM of [C_2_MIM][BF_4_] as the sole BGE at pH 3. Zhao et al. [214] used three ILs and hydroxypropyl-β-cyclodextrin (HP-β-CD) as the components of the BGE for the enantioseparation of itraconazole, ketoconazole, econazole and miconazole. Compared with [C_2_MIM][L-lactate] or [C_2_MPyrr][BF_4_], [C_12_MAmm][Cl] was the most effective. When this reagent was used along with HP-β-CD it allowed the resolutions of 3.8, 3.5, 2.8 and 2.5 for miconazole, econazole, ketoconazole and itraconazole, respectively, to be obtained.

**Figure 10:**
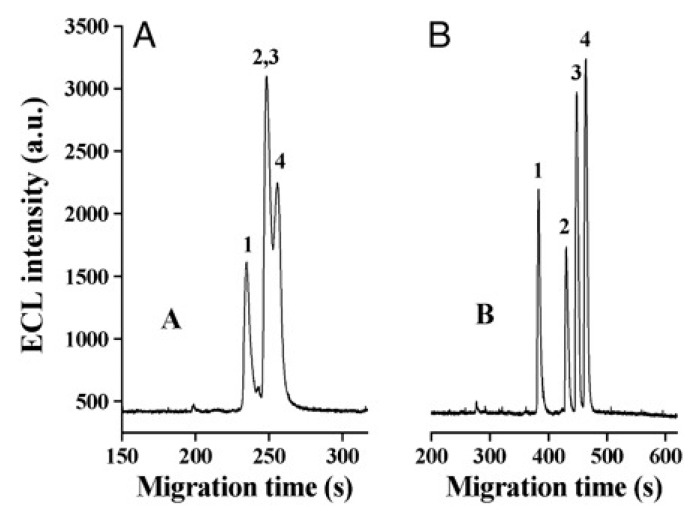
Electropherograms of four standard samples: (**A**) without IL in electrophoretic buffer; (**B**) with the use of 0.6% BIMPF_4_ in the electrophoretic buffer. Peak: 1, 10 µmol/L methylephedrine hydrochloride; 2, 40 µmol/L of thebaine; 3, 25 µmol/L codeine phosphate; 4, 15 µmol/L acetylcodeine. Conditions: electrophoretic buffer, 14 mmol/L phosphate–borax at pH 7.4; electrokinetic injection, 10 s × 10 kV; separation voltage, 15 kV; detection potential, 1.2 V; ECL solution, 5 mmol/L Ru(bpy)_3_^2+^ with 50 mmol/L PBS at pH 8.2. Figure adopted from [215] with permission.

In the paper published by Liu et al. [215], the effective chiral separation of racemic methyl-ephedrine hydrochloride, thebaine, codeine phosphate and acetylcodeine by capillary electrophoresis with electrochemical detection (CE-ECL) was observed when 0.6% [C_4_MIM][BF_4_] as the component of the BGE was applied (Figure 10). 

The developed method offered the quantification of four drug alkaloids in human urine samples with LODs from 1.4 × 10^−7^ to 6.3 × 10^−8^ mol/L. Jin et al. [216] reported the effective enantioseparation of propranolol, oxprenolol and pindolol by CE when a BGE containing the achiral IL—glycidyltrimethylammonium chloride ([GTMAmm][Cl]) as the modifier along with a dual CDs system based on 2,6-di-O-methyl-β-cyclodextrin (DM-β-CD) and 2,3,6-tri-O-methyl-β-cyclodextrin (TM-β-CD) was applied. The authors also used an on-line sample enrichment technique based on field-enhanced sample injection (FESI) for the improvement of sensitivity. The application of both approaches allowed the LODs of the enantiomers to be decreased from 0.10 to 0.65 nM. Finally, the developed CE method was successfully used for the analysis of spiked urine samples, with good recoveries.

**Figure 11:**
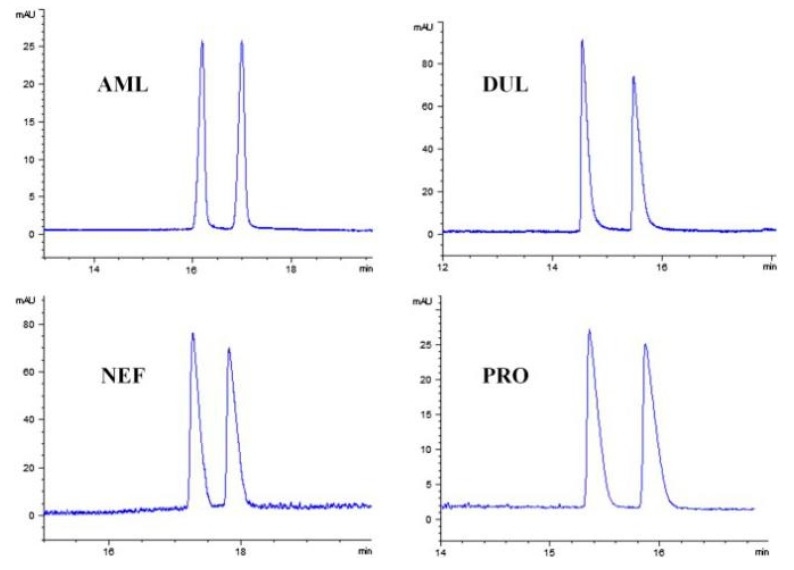
Chiral separation of all drug enantiomers in the optimized HP-β-CD/TMA-L-Arg synergic system. Conditions: focused-silica capillary, 50 cm (41.5 cm effective length) × 50 µm i.d; applied voltage, 20 kV; capillary temperature, 15 °C; BGE, 40 mM Tris/H_3_PO_4_ buffer solution (Ph 2.6) containing 20 mM HP-β-CD/TMA-L-Arg. Figure adopted from [219] with copyright permission.

Unfortunately, in many cases, the application of achiral ILs with a single chiral selector was not enough for the effective enantioseparation of the compounds of interest. An interesting alternative approach reported in the literature was using CILs which can possess either a chiral cation or achiral anion, or both. The application of these CILs in combination with traditional chiral selectors allows an extra “enantiorecognition” capability to be obtained while the capability of system modification is retained. In effect, a “synergistic system” occurs during electrophoretic separation, which can significantly improve the resolution of the analytes. The first paper reporting the use of this approach for the enantioseparation of pharmaceuticals was published by François et al. [217]. The authors developed and used two chiral choline-based ILs—ethylcholine bis(trifluoromethylsulfonyl)imide ([EtChol][Nf_2_T]) and phenylcholine bis(trifluoromethylsulfonyl)imide ([PhChol][Nf_2_T]) alone or in combination with DM-β-CD or TM-β-CD for the analysis of the anti-inflammatory drugs, 2-arylpropionic acids, as model compounds. The developed CILs were applied as BGE additives, chiral ligands and CSs. Moreover, the enantioseparation efficiency in respect to the type and concentrations of tested CILs and CDs, as well as the methanol addition to the BGE, were evaluated. The results indicated that the effective separation of the analytes was achieved only upon adding one of the CILs containing DM-β-CD or TM-β-CD and methanol to the BGE. Thus, the synergistic effect between the tested chiral choline-based ILs and CDs in the dual separation system was confirmed. In another study, two chiral synergistic systems based on tetramethylammonium-L-arginine (TMA-L-Arg)/glycogen and tetramethylammonium-L-aspartic acid (TMA-L-Asp)/glycogen were compared with the system containing achiral tetramethylammonium hydroxide (TMA-OH)/glycogen for the chiral separation of nefopam, citalopram and duloxetine [218]. Each tested IL/glycogen synergistic system gave better resolutions of the tested enantiomers compared to those observed for the separation using glycogen alone. However, the addition of TMA-L-Arg to the BGE composition was more effective than TMA-L-Asp, while the TMA-OH/glycogen separation system gave poorer resolution. Zhang et al. [219] tested tetramethylammonium-L-arginine (TMA-L-Arg), tetramethyl-ammonium-L-hydroxyproline (TMA-L-Hyp) and tetramethylammonium-L-isoleucine (TMA-L-Ile) as BGE additives in combination with HP-β-CD for the enantioseparation of amlodipine, nefopam, duloxetine and propranolol. The highest signals of the tested analytes and the best resolution was achieved using a 40 mM Tris/H_3_PO_4_ buffer solution (pH 2.6) containing 20 mM of HP-β-CD and 30 mM of TMA-L-Arg (Figure 11). Zuo et al. [220] reported the enantioseparation of twelve pharmaceuticals using 1-ethyl-3-methylimidazolium-L-lactate ([C_2_MIM][L-lactate]) and 1-butyl-3-methylimidazolium-L-lactate ([C_4_MIM][L-lactate]) in combination with β-CD in a BGE. The resolution was better in a dual system based on one of the tested CILs and β-CD compared to the β-CD alone, although the addition of [C_2_MIM][L-lactate] was more effective. Finally, the BGE composed of 20 mM of [C_2_MIM][L-lactate] and 10 mM of β-CD at pH 2.5 was selected as optimal for the separation of most analytes. 

Only the analysis of homatropine methylbromide was carried out on 30 mM of Tris-H_3_PO_4_ at pH 2.0 (more effective separation), while the enantiomers of venlafaxine and sibutramine were not baseline resolved. Kolobova et al. [221] confirmed that 1-butyl-3-methylimidazolium L-prolinate [C_4_MIM][L-Pro] as a CS in combination with 2-hydroxypropyl-β-cyclodextrin (2HP-β-CD) allowed a significant improvement in the chiral separation of carvedilol and propranolol.

Zhang et al. [222] designed a lactobionic acid LA-based IL, namely tetramethylammonium-lactobionate (TMA-LA), which was applied for the chiral separation of atenolol, metoprolol, propranolol, nefopam and duloxetine. In the study, three combinations, namely a single LA system, β-TMA chloride (TMA-Cl) system and TMA-LA IL system, were tested. The best results were achieved when the IL TMA-LA as the CS was applied. Finally, the BGE containing 40 mM of borax buffer, pH 7.6, 40% *v/v* methanol, 200 mM of TMA-LA and 20 kV applied voltage was selected as the most effective. Zhang et al. [223] tested L-alanine tert-butyl ester bis (trifluoromethane) sulfonamide (L-AlaC_4_Nf_2_T) and L-valine *tert*-butyl ester bis (trifluoromethane) sulfonamide (L-ValC_4_Nf_2_T) as additives to the BGE in combination with M-β-CD, HP-β-CD and glucose-β-CDs (Glu-β-CD) for the enantioseparation of naproxen, pranoprofen and warfarin. Compared to CDs alone, significantly better chiral recognitions of all analytes were obtained, although the resolutions of these dual systems were different. Moreover, the addition of organic modifiers to the BGE additionally improved selectivity. This was probably related to decreasing the EOF, which allowed interactions to be increased between AAILs, M-β-CD and the racemates. The best separations of the analytes were observed when 15 mM of CILs was introduced into the 30 mM sodium citrate/citric acid buffer solution at pH 5.0 containing 20 mM of M-β-CD and 20% ethanol as the organic modifier with a 20 kV applied voltage. The potential synergistic effects of L-AlaC_4_Nf_2_T and L-ValC_4_Nf_2_T were also checked in combination with vancomycin during the enantioseparation of naproxen, carprofen, ibuprofen, ketoprofen and pranoprofen [224]. Both dual synergic separation systems were also able more effectively to separate the enantiomers compared to the vancomycin-alone case. Xu et al. [225] applied tetramethylammonium-L-hydroxyproline (TMA-L-Hyp) with clindamycin phosphate (CP) for the separation of a racemic mixture of propranolol, nefopam, citalopram and chlorphenamine. The authors optimized the electrophoretic conditions in terms of the BGE composition, pH, voltage, temperature and UV parameters. The best results were obtained when the CE separation was carried out on an uncoated fused-silica capillary (50 cm total and 41.5 cm effective length × 50 μm i.d.) with a 40 mM borax buffer (pH 7.6) containing 80 mM of CP and 30 mM of TMA-L-Hyp and methanol (20% *v/v*). A voltage of 20 kV and a temperature of 20 °C were used. Nefopam, citalopram, chlorphenamine and propranolol were monitored at 289, 230, 265 and 237 nm, respectively. AAILs based on a tetramethylammonium cation were also tested with maltodextrin for the enantio-separation of pharmaceuticals belonging to different classes. For example, Yang et al. [226] used tetramethylammonium-D-pantothenate (TMA-D-PAN) and tetramethylammonium-D-quinate (TMA-D-QUI) as additives to the maltodextrin-based synergistic systems in a CE method developed for the analysis of racemic mixtures of nefopam, ketoconazole, econazole and voriconazole. For both of the CIL/maltodextrin systems, significantly improved R_s_ were observed for all the tested enantiomers, although TMA-D-PAN offered better separation results. This synergistic effect was probably related to a decrease in the density of the negative charge as an effect of the adsorption of the CIL cations on the surface of the capillary. This caused increasing complexation between the racemates and the CIL, which improved the resolution for all analytes.

Chen et al. [227] used tetramethylammonium-L-arginine (TMA-L-Arg) and tetramethyl-ammonium-L-aspartic acid (TMA-L-Asp) in combination with maltodextrin for the enantioseparation of nefopam, citalopram, cetirizine, duloxetine and ketoconazole. The most effective chiral separation was observed when a BGE composed of 60 mM of TMA-L-Arg, 7.0% maltodextrin in 50 mM of Tris-H_3_PO_4_ (pH 3.0) and with a voltage of 18.0 kV was applied. Zhang et al. compared the separation systems based on 1-butyl-3-methylimidazolium(T-4)-bis[(2S)-2-(hydroxy-κO)-3-methylbutanoato-κO]borate ([C_4_MIM][BLHvB]) and 1-butyl-3-methylimidazolium (T-4)-bis[(αS)-α-(hydroxy-κO)-4-methylbenzeneacetato-κO]borate ([C_4_MIM][BSMB]) along with HP-β-CD [228] as well as dextrin [229] as the CS in CE enantioseparations. In both studies, the addition of the CIL enabled the synergistic effect to occur between them and the used CS, which allowed better resolutions to be obtained and higher peak efficiencies compared to those calculated for the HP-β-CD or the dextrin alone. On the other hand, [C_4_MIM][BLHvB] was more effective than [C_4_MIM][BSMB]. This was probably related to the structure of the [C_4_MIM][BSMB] anion whose aromatic ring substituent could disturb chiral recognition.

An interesting approach was presented by Zhang et al. [230] who employed IL-dispersed NPs as buffer modifiers for the chiral separation of laudanosine, propranolol, amlodipine, citalopram and nefopam in CE. In the study, [C_4_MIM]BF_4_], ([C_4_MIM][PF_6_]), 1-dodecyl-3-methylimidazolium chloride ([C_12_MIM][Cl]) and 1-aminoethyl-3-methylimidazolium bromide ([C_2_NH_2_MIM][Br]) ILs were dispersed in multi-walled carbon nanotubes (ILs-MWNTs) and applied as the BGE modifier in combination with chondroitin sulfate E (CSE), as the CS. The obtained results indicated that significantly better separation, selectivity and peak shapes were achieved in the ILs-MWNTs modified system compared to that observed in CSE alone. The parameters affecting the electrophoretic separation were also investigated and optimized. The best results were obtained when CE was carried out on a 20 mM Tris/H_3_PO_4_ buffer solution containing 2.5% CSE and 2.4 µg/mL of ILs-MWNTs at pH 2.8–3.4 and with 15 kV applied voltage.

**Figure 12:**
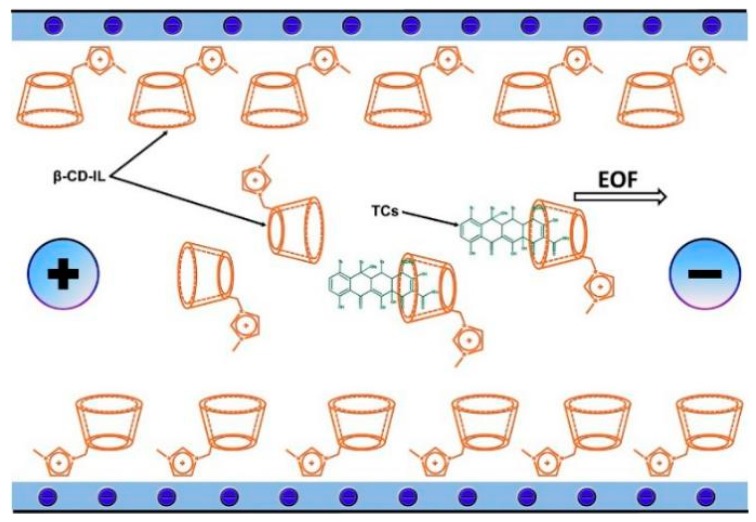
Mechanism of separation of four TCs using β-CD–IL as dynamic coating material. Figure adopted with permission from [63].

It should be highlighted that most of the studies described above indicated that the application of CILs alone as BGE modifiers was not able to effectively to separate the enantiomers. However, in the literature there are also a few reports describing the synthesis of novel CIL structures the activity of which was enough to achieve full resolution of drug enantiomers. For example, Yu et al. [231] synthesized a β-CDs-based CIL, 6-O-2-hydroxypropyltrimethylammonium-β-cyclodextrin tetra-fluoroborate ([HPTMA-β-CD][BF_4_]), and used it as a CS for the enantioseparation of eight pairs of drug enantiomers. The novel CIL offered higher solubility of the analytes in the BGE and gave better stabilization of reversed EOF in CE compared to the parent β-CDs, which allowed a higher intensity of the signals and a more effective resolution to be obtained. The results confirmed that the enantiomers of chlorpheniramine, brompheniramine, promethazine, liarozole, tropicamide, warfarin, pheniramine and bifonazole were more effectively separated with [HPTMA-β-CD][BF_4_] as the CS than with β-CDs. Recently, a report describing the synthesis of mono-6-deoxy-6-(3-methylimidazolium)-β-cyclodextrin tosylate (β-CDMIMOTs) CIL was also published by Zhou et al. [63]. The authors applied this new CIL as a coating material to modify the EOF in the CE method for the enantioseparation of oxytetracycline, tetracycline, chlortetracycline and doxycycline in environmental samples. The researchers achieved good separation of the analytes due to the multiple functions of β-CD-IL, which enabled the tetracyclines to be entrapped to form an inclusion complex (Figure 12).

Compared to β-CD alone, β-CD-IL offered better solubility in an aqueous buffer. A stable suppressed EOF in the capillary was also generated as the effect of the occurrence of hydrogen bonding and the electrostatic interaction with the capillary inner wall. The authors selected the best CE conditions for tetracycline separation, which were achieved when a BGE composed of 10 mmol/L, a pH 7.2 phosphate buffer and 20 mmol/L of β-CD-IL and electrochemical detection at 1 V was used. The developed CE method allowed the compounds of interest to be monitored in environmental water samples with LODs from 0.33 to 0.67 µmol/L.

### 5.2. Micellar Electrokinetic Chromatography

Considered as a mode of CE, micellar electrokinetic chromatography (MEKC) allows both neutral and charged analytes to be separated. In MEKC, the surfactant monomers are added to the run separation buffer above the critical micelle concentration (CMC), which allows aggregates called micelles to form as a pseudostationary phase. The separation process is based on differences between the analytes partitioning in a micellar stationary phase, and is related to the electrophoretic mobility of the compounds. Therefore, the neutral and hydrophobic analytes incorporated into the micelles gain an apparent electrophoretic mobility and will move at the same velocity as the micelle under electrophoretic conditions. This allows the neutral and charged compounds with the same charge-to-mass ratio to be separated because the migration time in MEKC is dependent on the electrophoretic velocity of the micelle, the distribution ratio and the EOF velocity. The use of additional BGE modifiers can increase efficiency and selectivity. ILs as BGE additions have become interesting alternatives because the long-chain part of the AAILs can act as a surfactant to form a micelle in the BGE when the level of ILs exceeds the CMC. Moreover, the electrostatic interaction between the acidic analyte and the cationic micelle (AAILs) offered a more effective enantiorecognition of the analytes. Higher concentrations of ILs may also be used compared to organic solvent surfactants because of higher conductivity, hydrophobicity and solvation, which decreases the risk of destroying the micellar system in MEKC. In the literature, there are a few papers reporting the use of ILs in MECK. For example, Wang et al. published two consecutive papers [232,233] demonstrating the combination of TM-β-CD with N-undecenoxycarbonyl-L-leucinol bromide (L-UCLB) CIL as a dual chiral selector for the enantiodiscrimination of fenoprofen, indoprofen, ketoprofen, suprofen and ibuprofen. In the study, different levels of CILs and TM-β-CD were tested. The results indicated that TM-β-CD alone could not resolve the enantioseparation of the racemates, whereas the addition of L-UCLB at a concentration of 1.5 to 2.0 mM to the BGE with TM-β-CD provided an excellent resolution. This was related to the competitive inhibition of the interaction between the CIL and the capillary wall in the presence of TM-β-CD. Cui et al. [234] used L-ethyl-3-methylimidazolium-L-lactate, [C_2_MIM][L-lactate] and 1-ethyl-3-methylimidazolium-L-(β)-lactate [C_2_MIM][DL-lactate] alone or in combination with HP-β-CD for the chiral resolution of ten analytes belonging to different classes of pharmaceuticals. The results confirmed that the best enantiorecognition was obtained when a BGE composed of 40 mM of HP-β-CD, 50 mM of NaH_2_PO_4_-H_3_PO_4_, pH 2.75, and 30 mM of [C_2_MIM][L-lactate] was used during the enantiomeric separation. Moreover, this effect was mainly correlated with the cationic activity of the IL, which played an important role in the increased resolution, whereas the anionic part of the CIL possessed a low influence on the chirality and nature of the enantioseparation. Su et al. [235] tested the addition of [C_4_MIM][Cl], [C_4_MIM][PF_6_], [C_4_MIM][Nf_2_T] and SDS as modifiers in the BGE during the optimization of MEKC conditions for the separation of seven benzodiazepines. The results confirmed that the BGE containing 170 mM of [C_4_MIM][Nf_2_T] and 10 mM of SDS offered the most effective selectivity and resolution of the compounds of interest. This was related to different degrees of association of the tested analytes, which gave a more satisfactory separation compared to the results observed using the IL or SDS alone. The anionic moiety of [C_4_MIM][Nf_2_T] probably played a dominant function during the separation process as a heteroassociation site for the benzodiazepines, while the SDS improved the resolution. The developed MEKC method allowed the analytes to be detected in human urine samples with LODs in the range of 2.74 to 4.42 µg/mL.

### 5.3. Non-Aqueous Capillary Electrophoresis

In recent years, non-aqueous capillary electrophoresis (NACE) has become an interesting separation technique because it allows the detection of water-insoluble analytes which cannot be measured in traditional aqueous CE. Additionally, the analysis time in NACE can be shortened because of the lower viscosity of the buffer solution and the higher EOF as well as the reduction of the electrophoretic current. Moreover, the application of organic solvents allows the analytes to be detected online by MS. As it was earlier mentioned, ILs possess some advantages over conventional organic solvent modifiers, such as good conductivity. Hence, using ILs in NACE can give a better separation effect. These possibilities were confirmed by Ma et al. [236] who applied an ephedrine-based CIL as the CS for the enantiomeric resolution of omeprazole and rabeprazole by NACE. A reversed EOF (anodic flow), probably caused by the adsorption of the cations onto the capillary wall, was observed when (+)-N,N-dimethylephedrinium-bis(trifluoromethanesulfon)imidate ([DMP]^+^[Nf_2_T]^−^) was added to the BGE. The best resolution was achieved with the BGE containing an acetonitrile-methanol mixture (60:40, *v/v*) and 60 mM of [DMP]^+^[Nf_2_T]^−^. The authors found that the enantioseparation was related to ion-pair interactions dependent on equilibrium constants between the negatively charged enantiomers and DMP cations. Moreover, hydrogen-bonding between the hydroxyl group of DMP^+^ and the sulfoxide group of the analytes as well as π–π and dipole–dipole interactions were responsible for the separation mechanism.

Summarizing, the application of ILs in electromigration techniques offers new opportunities to solve many analytical problems in the separation field. One of them is the chiral recognition of racemic mixtures of pharmaceuticals having different chemical structures and biological activity. The results of numerous studies based on drug standards confirmed the great potential of ILs in CE applications. On the other hand, there are relatively few reports describing the separations of drugs in real biological and environmental samples. This seems to be caused by the relatively low sensitivity of CE-based methods compared to LC and GC techniques, which may be not enough for many pharmaceutical, clinical and environmental applications. On the other hand, lower LOD values can be obtained in electromigration techniques supported by ILs, which allows a partial resolution for this analytical problem. Moreover, intensive progress is continuing systematically in developing new approaches for improving sensitivity in electromigration techniques based on techniques such as field-enhanced sample injection (FESI), field-amplified sample injection (FASI), field-amplified sample stacking (FASS) or a combination of simultaneous electrokinetic and hydrodynamic injection (SEHI) and field-enhanced sample injection in conjunction with a sweeping technique known as sequential stacking featuring sweeping (SSFS) [237,238]. Probably, when scientists apply both ILs and new technical resolutions in CE, it will allow the required sensitivity to be obtained for clinical and environmental studies. These studies are very important because both CE-based techniques and ILs are environmentally-friendly, so connecting them in one analytical tool could be an important factor supporting the protection of nature.

## 6. Current Trends and Future Perspectives

Pharmaceuticals possess high biological activity and they can take part in various types of interactions, which means that these substances have a huge influence on the functioning of both live organisms as well as whole ecosystems. Therefore, as it was mentioned in Section 1, it is very important to develop sensitive, selective, accurate and precise methods for reliable drug determination in biological and environmental samples. An interesting approach is the application of ILs during method development. According to the data presented in this review, there are several interesting trends in the application of ILs for the determination of pharmaceuticals. First of all, ILs are most often applied at the stage of sample preparation (Table 2). The vast majority of studies concerned the extraction (or actually microextraction) of biological and environmental samples. Moreover, the most common type of analyte extraction from both these matrices was DLLME. Researchers pay a lot of attention to improving these methods by introducing modifications using physical and chemical factors. As a result, they promote the development of environmentally-friendly solutions in the field of analytical chemistry and the improvement of validation parameters. Unfortunately, it should be noted that despite the development of various IL-based methods, the majority of procedures are still supported by organic solvents. In DLLME, their basic function is the dispersion of ionic liquids. In turn, because of the high viscosity of ILs, sample detection is only possible after dissolving the sample in MeOH, ACN and others. Thus, the application of ILs leads to improved validation parameters, but the developed methods are not completely eco-friendly. The results prove that despite moving in the right direction, this area requires further development. Improving the results is possible not only by proper sample preparation, but also by the application of ILs in chromatographic and electrophoretic techniques. The addition of ILs to mobile phases is the main way of using them for the determination of pharmaceutical drugs by chromatographic techniques. As the results show, the suppression of the interaction of silanol by use of ILs is a huge advance in the problematic analysis of basic drugs (Table 3). The use of ILs in the BGE in electrophoretic techniques, which in many respects are compatible with green chemistry, although their sensitivity still remains a challenging task for the analyst, seems to be promising. It may be surprising that despite the existence of commercially available and described methods for the self-preparation of IL-based chromatographic columns and capillaries for electrophoresis, such methods of their use is very rare for pharmaceuticals. If the huge potential of ILs is to be discovered, it should also be noted that in addition to the above detection methods, researchers are trying to use them with other chromatographic techniques. Although such applications are not yet widespread in the analysis of pharmaceuticals, their dynamic development may cause such experiments to be performed in the future. In addition to trends in the design of analytical methods, the qualification of ILs with similar structures to a specific stage of analysis is the constant rule. In many works, optimization concerns the selection of a specific IL from a large diverse group of IL molecules. However, according to the data presented in different reports, the final optimization effect leads to the selection of the same IL. For example, an IL with hydrophobic properties was sought for liquid-phase extraction and the best results were often achieved for the imidazolium cation and anion [PF_6_]. In turn, as an addition to the mobile phase, the selection of the IL [PF_6_] was not suitable due to too strong adsorption on the column and was replaced by [BF_4_]. It must be highlighted that these are trends for most, but not all papers (detailed in Table 2 and Table 3). However, the fact is that despite access to a vast amount of ILs, only a few have been tested in experiments, and the final selection focuses on a small number. As mentioned, the samples are analyzed by various chromatographic and electrophoretic techniques, while a UV detector is almost always used for analyte detection, rarely FL and almost never MS/MS.

The above examples confirm that there are no ideal solutions in the design of analytical methods for the determination of pharmaceuticals in biological and environmental samples. However, in the case of ILs, their advantages over disadvantages and also the incomplete data on them prove the need for continuous interest and development in this area.

## 7. Conclusions

ILs as molecules with unique properties have been the subject of increased interest in recent years. Undoubtedly, the key issue is “green chemistry”, which has set the direction of current research. Due to their huge potential, it is natural to use ILs in the search for solutions to many problems in modern laboratories, including their participation in analyzing pharmaceuticals in real samples. The use of analytical methods at various stages confirms the universality and enormous potential of this “solvent design”. The application of various chromatographic and electrophoretic techniques and extraction methods together with the possibility of the use of ILs for a wide range of analytes prove that their contribution to the development of analytical methods is not overestimated. At the same time, the limitations that appear during their use show that success in experiments is not easy and this field of research requires further development.

## Figures and Tables

**Table 1:** List of ILs used in modern laboratory for drug analysis.

Cation Structure	Full Name		Abbreviations
	Cations	Anions	
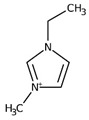	1-ethyl-3-methylimidazolium	hexafluorophosphate, tetrafluoroborate, chloride, bromide, bis(trifluoromethylsulfonyl)imide, methyl sulfate	[C_2_MIM][PF_6_], [C_2_MIM][BF_4_], [C_2_MIM][Cl], [C_2_MIM][Br], [C_2_MIM][Nf_2_T], [C_2_MIM][CH_3_(SO_4_)]
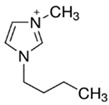	1-butyl-3-methylimidazolium	hexafluorophosphate, tetrafluoroborate, chloride, bromide, bis(trifluoromethylsulfonyl)imide, nitrate, methyl sulfate, octyl sulfate, trifluoromethanesulfonate, dimethyl phosphate, hydroxide	[C_4_MIM][PF_6_], [C_4_MIM][BF_4_], [C_4_MIM][Cl], [C_4_MIM][Br], [C_4_MIM][Nf_2_T], [C_4_MIM][NO_3_], [C_4_MIM][CH_3_(SO_4_)], [C_4_MIM][C_8_H_17_(SO_4_)], [C_4_MIM][CF_3_SO_4_], [C_4_MIM][DMP], [C_4_MIM][OH]
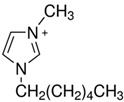	1-hexyl-3-methylimidazolium	hexafluorophosphate, tetrafluoroborate, chloride, bromide, bis(trifluoromethylsulfonyl)imide, tris(pentafluoroethyl)trifluoro-phosphate	[C_6_MIM][PF_6_], [C_6_MIM][BF_4_], [C_6_MIM][Cl], [C_6_MIM][Br], [C_6_MIM][Nf_2_T], [C_6_MIM][TFP]
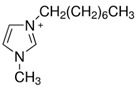	1-octyl-3-methylimidazolium	hexafluorophosphate, tetrafluoroborate, chloride, bromide, bis(trifluoromethylsulfonyl)imide	[C_8_MIM][PF_6_], [C_8_MIM][BF_4_], [C_8_MIM][Cl], [C_8_MIM][Br], [C_8_MIM][Nf_2_T]
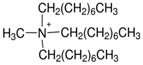	methyltrioctylammonium	tetrachloroferrate, tetrachloromanganate(II)	[C_8_MAmm][FeCl_4_], [C_8_MAmm][MnCl_4_^2−^]
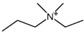	ethyl-dimethyl-propylammonium	bis(trifluoromethylsulfonyl)imide	[NEMMP][Nf_2_T]
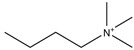	1-butyl-3-methylammonium	bis(trifluoromethylsulfonyl)imide	[C_4_M_3_Amm][Nf_2_T]
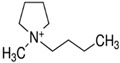	1-butyl-1-methylpyrrolidinium	bis(trifluoromethylsulfonyl)imide, tetracyanoborate, tris(pentafluoroethyl)trifluoro-phosphate	[C_4_MPyrr][Nf_2_T], [C_4_MPyrr][B(CN)_4_] [C_4_MPyrr][TFP]
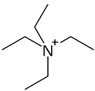	tetraethylammonium	tetrafluoroborate	[(C_2_H_5_)_4_N][BF_4_]
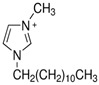	1-dodecyl-3-methylimidazolium	chloride	[C_12_MIM][Cl]
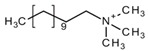	1-dodecyl-3-methylammonium	chloride	[C_12_MAmm][Cl]
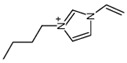	1-vinyl-3-butylimidazolium	chloride	[ViC_4_MIM][Cl]
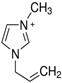	1-allyl-3-ethylimidazolium	bromide	[AC_2_MIM][Br]
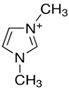	1,3-dimethylimidazolium	methyl sulfate	[MMIM]][CH_3_(SO_4_)]
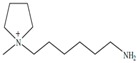	1-(6-amino-hexyl)-1-methylpyrrolidinium	tris(pentafluoroethyl)trifluoro-phosphate	[C_6_NH_2_MPyrr][TFP]
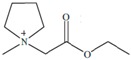	1-ethoxycarbonyl-methyl-1-methyl-pyrrolidinium	tris(pentafluoroethyl)trifluoro-phosphate	[ECMMPyrr][TFP]
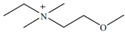	methoxyethyl-dimethylethyl-ammonium	tris(pentafluoroethyl)trifluoro-phosphate	[MOEDEAmm][TFP]
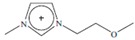	1-methoxyethyl-3-methylimidazolium	tris(pentafluoroethyl)trifluoro-phosphate	[MOEMIM]][TFP]
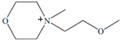	1-methoxyethyl-1-methylmorpholinium	tris(pentafluoroethyl)trifluorophosphate	[MOEMMO][TFP]
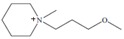	1-methoxypropyl-1-methylpiperidinum	tris(pentafluoroethyl)trifluorophosphate	[MOPMPP][TFP]
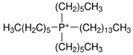	trihexyltetradecylphosphonium	tetrachloromanganate(II), dicyanamide, bis(trifluoromethanesulfonyl) imide	[P_6,6,6,14_^+^]_2_[MnCl_4_^2−^], [P_6,6,6,14_^+^][N(CN)_2_], [P_6,6,6,14_^+^][Nf_2_T]
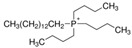	tributyl(tetradecyl)phosphonium	*p*-dodecylbenzenesulfonate	[P_4,4,4,14_^+^][DDBS])
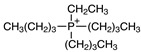	tributyl(ethyl)phosphonium	diethylphosphate	[P_2,4,4,4_^+^][(2O)_2_PO_2_]

**Table 2:** Summary of the IL applications in liquid-phase microextraction drugs from biological and environmental samples.

Drug(s)	Matrices	Tested Ionic Liquids	Extraction Solvent	Analytical Technique	LOD [ng/mL]	Efficiency [%]	Ref.
**LLE**							
**Environmental samples**							
Ranitidine, nizatidine	River water, wastewater	**[C_4_MIM][Nf_2_T] [C_4_MIM][PF_6_]**	MeOH	HPLC-UV	90 430	100.4 101.2	[68]
**IL-DLLME**							
**Biological samples**							
NSAIDs	Human urine	**[C_4_MIM][PF_6_]** [C_6_MIM][PF_6_] [C_8_MIM][PF_6_]	MeOH	HPLC-UV	8.3–32	36.8–42.3	[70]
Anti-hypertensive drugs	Rat serum	**[C_4_MIM][PF_6_]** [C_6_MIM][PF_6_] [C_4_MIM][BF_4_] [C_6_MIM][Cl] [C_2_MIM][CH_3_(SO_4_)]	Acetone	HPLC-PDA	15–20	92.8–98.5	[80]
Balofloxacin	Rat serum	**[C_4_MIM][PF_6_]** [C_6_MIM][PF_6_] [C_4_MIM][BF_4_] [C_6_MIM][Cl] [C_4_MIM][Br] [C_2_MIM][CH_3_(SO_4_)]	ACN	HPLC-DAD	10	99.5	[81]
Rifaximin	Rat serum	**[C_4_MIM][PF_6_]** [C_4_MIM][BF_4_] [C_6_MIM][Cl] [C_4_MIM][Br] [C_2_MIM][CH_3_(SO_4_)]	MeOH	HPLC-DAD	10	99.5	[82]
Ofloxacin	Human urine and plasma, tablets	**[C_6_MIM][PF_6_]**	Ethanol	SFIS	29	89.5–93	[86]
Tetracycline	Eggs	**[C_4_MIM][PF_6_]** [C_6_MIM][PF_6_] [C_8_MIM][PF_6_] [IMIM][PF_6_]	ACN	HPLC-DAD	2–12	58.6–95.3	[87]
Fluoroquinolone	Chicken, pork and fish meat	**[C_4_MIM][PF_6_]** [C_6_MIM][PF_6_] [C_8_MIM][PF_6_]	ACN	HPLC-DAD	0.5–1.1	60.4–96.3	[88]
Nifurtimox, benznidazole	Human plasma	**[C_8_MIM][PF_6_]** [C_4_MIM][PF_6_] [C_6_MIM][PF_6_]	MeOH	HPLC-UV	15.7 3.66	98 79.8	[72]
Nifurtimox, benznidazole	Human breast milk	**[C_8_MIM][PF_6_]**	MeOH	HPLC-UV	90 60	89.7 77.5	[73]
Sildenafil, Vardenafil, Aildenafil	Human plasma	**[C_8_MIM][PF_6_]** [C_4_MIM][PF_6_]	MeOH	HPLC-UV	0.92–2.69	100.4–103.9	[74]
Ephedrine, Ketamine	Human urine	**[C_4_MIM][PF_6_]** [C_6_MIM][PF_6_] [C_8_MIM][PF_6_]	ACN	HPCE	210 390	79–90	[89]
Daclatasvir	Rat serum	**[C_4_MIM][PF_6_]** [C_6_MIM][PF_6_] [C_4_MIM][BF_4_] [C_4_MIM][Br] [C_6_MIM][Cl] [C_2_MIM][CH_3_(SO_4_)]	ACN	HPLC-DAD	15	99.4	[90]
**Environmental samples**							
NSAIDs, acetazolamide, caffeine, sulfonamides, carbamazepine, gemfibrozil	Tap water, creek water	**[C_6_NH_2_MPyrr][TFP]** [C_4_MIM][Cl] [C_4_MIM][Nf_2_T] [ECMMPyrr][TFP] [MOEDEAmm][TFP] [MOEMIM][TFP] [MOEMMO][TFP] [MOEMPyrr][TFP] [MOPMPP][TFP]	MeOH/Acetone	HPLC-UV	0.1–55.1	91–110	[92]
Triclosan, triclocarbon	Tap water, wastewater	**[C_6_MIM][PF_6_]** [C_4_MIM][BF_4_]	MeOH	LC-MS/MS	0.040–0.58	70.0–103.5	[93]
**DLLME modifications**							
**Biological samples**							
*US-IL-DLLME*							
Salmeterol	Dried blood spot	**[C_4_MIM][PF_6_]** [C_6_MIM][PF_6_] [C_8_MIM][PF_6_]	MeOH	HPLC-FL	0.30	90	[71]
Citalopram, nortriptyline	Human plasma	**[C_8_MIM][PF_6_]** [C_4_MIM][PF_6_] [C_6_MIM][PF_6_]		HPLC-PDA	10 6	90–92	[79]
Venlafaxine, amitriptyline	Human plasma	**[C_8_MIM][PF_6_]** [C_4_MIM][PF_6_] [C_6_MIM][PF_6_]		HPLC-DAD	0.5 0.8	91.4–92.6	[83]
Ulipristal	Mice serum, tablets	**[C_8_MIM][PF_6_]** [C_4_MIM][PF_6_] [C_6_MIM][PF_6_]		HPLC-UV	6.8 9.3	95	[75]
Benzodiazepines and benzodiazepine-like	Human blood, post-mortem human blood	**[C_4_MIM][PF_6_]** [C_6_MIM][PF_6_] [C_8_MIM][PF_6_]		LC-MS/MS	0.03–4.74	24.7–126.2	[15,76,77]
Antidepressants	Human blood	**[C_4_MIM][PF_6_]** [C_6_MIM][PF_6_] [C_8_MIM][PF_6_] [C_4_MPyrr][Nf_2_T] [C_4_M_3_Amm][Nf_2_T]		LC-MS/MS	1–2	53.11–132.98	[78]
*DLLME (rapid shooting)*							
Danazol	Mice serum, capsules	**[C_8_MIM][PF_6_]** [C_4_MIM][PF_6_] [C_6_MIM][PF_6_]		UV	55 54	90.5–103.4	[84]
*TCIL-DLPME*							
Piroxicam	Human urine, plasma, and tablets	**[C_6_MIM][PF_6_]**		SFIS	46	95.2–104	[85]
**Environmental samples**							
*US-IL-DLLME*							
Lovastatin, simvastatin	Tap water, lake water, river water	**[C_6_MIM][PF_6_]**	MeOH	HPLC-UV	0.17 0.29	90.0–102.2, 80.5–112.0	[95]
β-Blockers NSAIDs	Wastewaters	**[C_8_MIM][PF_6_]** [C_4_MIM][PF_6_] [C_6_MIM][PF_6_]	ACN	LC-MS	0.0002–0.060	88–111	[97]
Fluoroquinolones	Groundwater	**[C_8_MIM][PF_6_]** [C_4_MIM][PF_6_] [C_6_MIM][PF_6_]	MeOH	HPLC-FL	0.0008–0.013	105–107	[98]
NSAIDs	Tap water, drinking water	**[C_8_MIM][PF_6_]**, [C_4_MIM][PF_6_]	MeOH	UHPSFC-PDA	0.62–7.69	81.4–107.5	[99]
*MADLLME*							
Derivatization of sulfonamides	River water	**[C_6_MIM][PF_6_]**, [C_4_MIM][PF_6_], [C_8_MIM][PF_6_]	MeOH	HPLC-FD	0.011–0.018	95.0–110.8	[100]
*MIL-DLLME*							
Acetaminophen sulfamethoxypyridazine, phenacetin, ketoprofen	Lake water, river water	**[P_6,6,6,14_^+^]_2_[MnCl_4_^2−^]** [Aliquat^+^]_2_[MnCl_4_^2−^] [C_8_MAmm][MnCl_4_^2−^]	ACN/MeOH	HPLC-UV	0.25–1.0	42.9–114.7	[94]
*IL-DLLME-μ-SPE*							
Antidepressant drugs	Canal water	**[C_6_MIM][TFP]** [C_6_MIM][Nf_2_T]	Methanol	HPLC-UV	0.3–1.0	94.3–114.7	[101]
*IL-DLLME-SDS*							
Tetracyclines	River water, fishpond water, leaching water	**[C_4_MIM][PF_6_]** [C_6_MIM][PF_6_] [C_8_MIM][PF_6_]	Methanol	UHPLC-TUV	0.031–0.079	55.1–96.3	[96]
*IL/IL-DLLME*							
Triclocarbon, triclosan	Tap, river, snow, Lake water	Hydrophobic: [C_4_MIM][PF_6_] [C_6_MIM][PF_6_] **[C_8_MIM][PF_6_]** Hydrophilic: [C_2_MIM][BF_4_] **[C_4_MIM][BF_4_]** [C_4_MIM][NO_3_]	-	LC-MS/MS	0.23 0.35	88–111	[102]
NSAIDs	Tap water, River water	**[C_4_MIM][BF_4_] [C_4_MIM][PF_6_]** [NEMMP][Nf_2_T] [MOEDEA][TFP]	Methanol	HPLC-DADHPLC-FL	17–95	89–103	[103]
**Other liquid phase extraction**							
**Biological samples**							
*IL-SE-UE-ME*							
Doxepin, perphenazine	Human urine	**[C_6_MIM][PF_6_]** [C_6_MIM][Nf_2_T] [C_4_MIM][PF_6_]		HPLC-MWD	100 1000	89–98	[104]
*IL/IL LPME*							
Sulfonamides	Human, chicken, rabbit, cow, pig blood	**[C_4_MIM][BF_4_] [C_6_MIM][PF_6_]**		HPLC-UV	3.77–5.21	90–113	[105]
*dLPME*							
NSAIDS	Human urine	**[C_4_MIM][PF_6_]**	ACN	HPLC-UV	38–70	72.8–90.3	[91]
Phenothiazines	Human urine	**[C_4_MIM][PF_6_]**	ACN	HPLC-UV	21–60	72–98	[106]
**Environmental samples**							
*SADBME*							
Diclofenac, ibuprofen	Wastewater treatment plant, river and lake water	**[C_8_MAmm][FeCl_4_]**	ACN	HPLC-DAD			[64]
*ILSVA-SME*							
Glucocorticoids	Mineral water, lake water, tap water	**[C_4_MIM][PF_6_]**	ACN	HPLC-DAD	4.11–7.50	≥97.24	[107]
*MA-LLME-SIL*							
Sulfonamides	Tap water, lake water, river water, pool water	**[C_2_MIM][PF_6_]**	ACN	HPLC-UV	0.33–0.85	75.1–115.8	[108]
Sulfonamides	Wastewater paddy water, river water	**[C_8_MIM][PF_6_]**		HPLC-UV	0.1–0.4		[109]
*(MBA)-LPME*							
Glucocorticoids	Wastewater	**[C_4_MIM][CH_3_(SO_4_)]** [C_4_MIM]BF_4_] [C_4_MIM][Cl] [C_4_MIM][PF_6_] [C_4_MPyrr][TFP] [C_6_MIM][TFP]	MeOH	UHPLC-MS/MS	0.0128–0.0470	49.40–83.1	[110]
*IL-AF-μ-EME*							
Antidepressants	Tap water, river water	**[C_6_MIM][PF_6_]** [C_8_MIM][PF_6_]	MeOH/ACN	HPLC-UV	0.4	88.2–111.4, 90.9–107	[111]
*IL-IDME*							
Amitriptyline	Hospital wastewater	**[C_6_MIM][PF_6_]**	MeOH/ACN	HPLC-UV	4.0	85.12	[16]
**ATPS**							
**Biological samples**							
Sulfonamides	Milk (from supermarket)	**[C_4_MIM][BF_4_]** [C_2_MIM][BF_4_] [C_6_MIM][BF_4_] [C_8_MIM][BF_4_]	ACN	HPLC-UV	2.04–2.84	72.3–108.9	[112]
Sulfonamides	Pig, rabbit, cow, chicken and human blood	**[C_6_MIM][Cl]** [C_4_MIM][Cl] [C_8_MIM][Cl]		HPLC-UV	2.45–4.13	85.5–110.9	[67]
**Environmental samples**							
*IL-ATPF*							
Chlorampheni-col	Lake water, feed water	**[C_4_MIM][Cl]** [C_8_MIM][Cl] [C_4_MIM][BF_4_]		HPLC-UV	0.1	97.1–101.9	[113]
*MILATPs*							
Chloramphenicol	River water	**[TMG][TEMPO-OSO_3_]**		HPLC-UV	0.14	94.6–99.7	[114]

[C_2_MIM]: 1-Ethyl-3-methylimidazolium; [C_4_MIM]: 1-butyl-3-methylimidazolium; [C_6_MIM]: 1-hexyl-3-methylimidazolium; [C_8_MIM]: 1-octyl-3-methylimidazolium; [MMIM]: 1,3-dimethylimidazolium; [C_4_MPyrr]: 1-butyl-1-methylpyrrolidinium; [C_4_M_3_Amm]: 1-butyl-3-methylammonium; [IMIM]: 1-isopentene-3-methylimidazolium; [Nf_2_T]: bis(trifluoromethylsulfonyl)imid; [BF_4_]: tetrafluoroborate; [PF_6_]: hexafluorophosphate; [Cl]: chloride; [CH_3_(SO_4_)]: methylsulfate; HPCE: high performance capillary electrophoresis; HPLC-FL: high-performance liquid chromatography and fluorescence detection; HPLC-UV: high-performance liquid chromatography and ultraviolet detection; HPLC-DAD: high-performance liquid chromatography and diode array detection; LC-MS/MS: liquid chromatography—tandem mass spectrometry; UV: ultraviolet spectroscopy; HPLC-MWD: high-performance liquid chromatography and multiple wavelength detector; HPLC-PDA: high-performance liquid chromatography and photo-diode array detector; SFIS: stopped-flow injection spectrofluorimetry; TCIL-DLPME: temperature-controlled ionic liquid dispersive liquid phase microextraction; [C_6_NH_2_MPyrr]: 1-(6-aminohexyl)-1-methylpyrrolidinium; [TFP]: tris(pentafluoroethyl)trifluorophosphate; [ECMMPyrr]: 1-ethoxycarbonylmethyl-1-methylpyrrolidinium; [MOEDEAmm]: methoxyethyl-dimethyl-ethylammonium; [MOEMIM]: 1-methoxyethyl-3-methylimidazolium; [MOEMMO]: 1-methoxyethyl-1-methylmorpholinium; [MOPMPP]: 1-methoxypropyl-1-methylpiperidinum; [Aliquat^+^]_2_[MnCl_4_^2−^]: aliquat tetrachloromanganate(II); [C_8_MAmm]: methyltrioctylammonium; [P_6,6,6,14_^+^]: trihexyltetradecylphosphonium; TUV: tunable ultraviolet detection; UHPLC: ultra-high pressure liquid chromatography; [MOEDEAmm]: ethyl-dimethyl-(2-methoxyethyl)ammonium; [NEMMP]: ethyl-dimethyl-propylammonium; MADLLME: microwave-assisted dispersive liquid–liquid microextraction; [TMG]: 1,1,3,3-tetramethylguanidine; [TEMPO]: 2,2,6,6-tetramethyl piperidine 1-oxyl free radical; IL-AF-µ-EME: Ionic liquid-impregnated agarose film two-phase micro-electrodriven membrane extraction; IL-ATPF: Ionic liquid–salt aqueous two-phase flotation; [DI-SPME]: direct-immersion solid-phase microextraction.

**Table 3:** Summary of the HPLC methods for the determination of pharmaceuticals in biological and environmental samples supported by the addition of ILs to the mobile phase.

Drug(s)	Matrice(s)	Tested Ionic Liquids	Apparatures	Stationary Phase	Ref.
Psychotropic drugs	Human serum	**[C_4_MIM][BF_4_]**	HPLC-DAD λ = 240 nm	Synergi Polar RP 80A (150 × 4.6 mm, 5 µm)	[140]
Fluoroquinolone antibiotics	Bovine, ovine and caprine milk	**[C_2_MIM][BF_4_],** [C_4_MIM][BF_4_]	HPLC-FL λ_ex_ = 280 nm λ_em_ = 450 nm	RP Nova-Pak C18 (150 × 3.9 mm, 4 µm)	[141]
Fluoroquinolone antibiotics	Mineral and tap water	[C_2_MIM][BF_4_], **[C_4_MIM][BF_4_]**, [C_6_MIM][BF_4_], [C_8_MIM][BF_4_], [(C_2_H_5_)_4_N][BF_4_]	HPLC-FL λ_ex_ = 280 nm λ_em_ = 450 nm	RP Nova-Pak C18 (150 × 3.9 mm, 4 μm)	[142]
Antiretroviral drugs	Rats plasma	**[C_4_MIM][BF_4_]**, [C_4_MIM][Br], [C_4_MIM][C_8_H_17_(SO_4_)], **[C_2_MIM][CH_3_(SO_4_)]**, [C_6_MIM][Cl]	HPLC-DAD	monolithic RP-18e column (250 × 4.6 mm, porous material)	[144]
Antidepressants	Urine samples	[C_4_MIM][BF_4_], [C_4_MIM][PF_6_], **[C_4_MIM][CF_3_SO_4_]**,	HPLC-UV λ = 254 nm	RP Eclipse X-DB-C8 (150 × 4.6 mm)	[145]
Ofloxacin, sparfloxacin, moxifloxacin, levofloxacin, *p*-amino-salicylic acid, ketoprofen, ibuprofen	Human plasma	[C_4_MIM][Cl], **[C_6_MIM][Cl]**, [C_8_MIM][Cl], [C_12_MIM][Cl], [C_6_MIM][BF_4_]	HPLC-DAD λ = 235–375 nm	Luna C18(150 × 4.6 mm, 5 μm)	[146]
Tricyclic antidepressants	Tablets	**[C_6_MIM][Cl]**, [C_6_MIM][BF_4_]	HPLC-UV λ = 254 nm	Zorbax Eclipse XDB C18 and C8 (150 × 4.6 mm, 5 µm)	[147]
β-Blockers		[C_2_MIM][Cl], [C_4_MIM][Cl], [C_6_MIM][Cl]	HPLC-UV λ = 254 nm	Zorbax Eclipse XDB (150 × 4.6 mm, 5 µm)	[6]
β-Blockers		**[C_6_MIM][Cl]**	HPLC-UV λ = 254 nm, λ = 300 nm (timolol)	Zorbax Eclipse XDB C18 (150 × 4.6 mm, 5 µm)	[7]
Quinine, fluphenazine, thioridazine, chlorpromazine, trifluopromazine, phenazoline, tiamenidine, naphazoline propiomazine		[C_2_MIM][BF_4_], [MMIM][CH_3_(SO_4_)]	HPLC-DAD λ = 254 nm	LiChrospher RP-18 (250 × 4.6 mm, 5 µm)	[143]
β-Blockers		[C_4_MIM][BF_4_], **[C_6_MIM][BF_4_]**	HPLC-UV λ = 254 nm	Zorbax SB C18 X-Terra MS C18 Kromasil Lichrospher, Nucleosil, Spherisorb	[148]
β-Blockers		[C_2_MIM][Cl], [C_4_MIM][Cl], **[C_6_MIM][Cl]**, [C_2_MIM][BF_4_], [C_4_MIM][BF_4_], **[C_6_MIM][BF_4_]**, [C_2_MIM][PF_6_]	HPLC-DADλ = 254 nm, λ = 300 nm (timolol)	Kromasil C18 (150 × 4.6 mm, 5 µm)	[149]
β-Blockers		[C_2_MIM][PF_6_], [C_4_MIM][PF_6_], [C_4_MIM][BF_4_], **[C_6_MIM][BF_4_]**	HPLC-UV λ = 254 nm, λ = 300 nm (timolol)	Kromasil C18 (150 × 4.6 mm, 5 µm)	[150]
Tricyclic antidepressants		**[C_4_MIM][PF_6_], [C_4_MIM][Cl],**	HPLC-DAD λ = 254 nm	Gemini-NX C18 (150 x 4.6 mm, 5 µm)	[151]
β-Lactam antibiotics		**[C_4_MIM][BF_4_]**, [C_6_MIM][BF_4_], [C_8_MIM][BF_4_]	HPLC-UV λ = 254 nm	RS Tech C18 (250 × 4.6 mm, 5 μm)	[152]
β-Blockers		**[C_4_MIM][BF_4_]**, [C_8_MIM][BF_4_], [C_4_MIM][PF_6_]	HPLC-UV λ = 254 nm	RP Kromasil C18 (150 × 4.6 mm, 5µm)	[153]
Neuroleptic Drugs		[C_2_MIM][PF_6_], **[C_4_MIM][PF_6_]**, [C_4_MIM][Cl]	HPLC-DAD	Zorbax Extend-C18 (150 × 4.6 mm, 5 µm)	[154]
Naphazoline, phenazoline, chlorpromazine, fluphenazine; propiomazine, thioridazine		[C_6_MIM][BF_4_], [C_8_MIM][BF_4_], [MMIM][CH_3_(SO_4_)],	HPLC-DAD λ = 254 nm	LiChrospher RP-18 (250 × 4.6 mm, 5 µm)	[155]

[(C_2_H_5_)_4_N]: tetraethylammonium; [C_8_H_17_(SO_4_)]: octylsulfate; [C_12_MIM]: 1-dodecyl-3-methylimidazolium. (The other abbreviations explained under Table 2).

**Table 4:** Selected commercial IL capillary GC columns with matrix active groups.

GC Capillary Column	Matrix Active Group
SLB-IL59	1,12-Di(tripropylphosphonium)dodecane bis(trifluoromethylsulfonyl)imide
SLB-IL60	1,12-Di(tripropylphosphonium)dodecane bis(trifluoromethylsulfonyl)imide
SLB-IL61	1,12-Di(tripropylphosphonium)dodecane bis(trifluoromethylsulfonyl)imide trifluoromethylsulfonate
SLB-IL76	Tri(tripropylphosphoniumhexanamido)triethylamine bis(trifluoromethylsulfonyl)imide
SLB-IL82	1,12-Di(2,3-dimethylimidazolium)dodecane bis(trifluoromethylsulfonyl)imide
SLB-IL110	1,9-Di(3-vinylimidazolium)nonane bis(trifluoromethylsulfonyl)imide
SLB-IL111	1,5-Di(2,3-dimethylimidazolium)pentane bis(trifluoromethylsulfonyl)imide
SLB-ILD3606	1,5-Di(2,3-dimethylimidazolium)pentane bis(trifluoromethylsulfonyl)imide

## References

[1] Ma W., Row K.H. (2019). Simultaneous determination of levofloxacin and ciprofloxacin in human urine by ionic-liquid-based, dual-template molecularly imprinted coated graphene oxide monolithic solid-phase extraction. J. Sep. Sci..

[2] Gorynski K. (2019). A critical review of solid-phase microextraction applied in drugs of abuse determinations and potential applications for targeted doping testing. TrAC Trends Anal. Chem..

[3] Boyacı E., Rodríguez-Lafuente A., Gorynski K., Mirnaghia F., Souza-Silva E.A., Heind D., Pawliszyn J. (2015). Sample preparation with solid phase microextraction and exhaustive extraction approaches: Comparison for challenging cases. Anal. Chim. Acta.

[4] Kokosa J.M. (2015). Recent trends in using single-drop microextraction and related techniques in green analytical methods. TrAC Trends Anal. Chem..

[5] Yamini Y., Safari M., Shamsayei M. (2018). Simultaneous determination of steroid drugs in the ointment via magnetic solid phase extraction followed by HPLC-UV. J. Pharm. Anal..

[6] Carda-Broch S., García-Alvarez-Coque M.C., Ruiz-Angel M.J. (2018). Extent of the influence of phosphate buffer and ionic liquids on the reduction of the silanol effect in a C18 stationary phase. J. Chromatogr. A.

[7] Peris-García E., Alvarez-Coque M.C.G., Carda-Broch S., Ruiz-Angel M.J. (2019). Effect of buffer nature and concentration on the chromatographic performance of basic compounds in the absence and presence of 1-hexyl-3-methylimidazolium chloride. J. Chromatogr. A.

[8] Ren Y.M., Yang R.C. (2016). Efficient method for the synthesis of 3-arylbenzoquinoline, pyranoquinoline, and thiopyranoquinoline derivatives using PEG1000-based dicationic acidic ionic liquid as recyclable catalyst via a one-pot multicomponent reaction. Synth. Commun..

[9] Halayqa M., Zawadzki M., Domańska U., Plichta A. (2019). Polymer—Ionic liquid—Pharmaceutical conjugates as drug delivery systems. J. Mol. Struct..

[10] Abbott A.P., Frisch G., Ryder K.S. (2013). Electroplating Using Ionic Liquids. Annu. Rev. Mater. Res..

[11] Pei Y.C., Wang J.J., Xuan X.P., Fan J., Fan M. (2007). Factors Affecting Ionic Liquids Based Removal of Anionic Dyes from Water. Environ. Sci. Technol..

[12] Kobylis P., Lis H., Stepnowski P., Caban M. (2019). Spectroscopic verification of ionic matrices for MALDI analysis. J. Mol. Liq..

[13] Shekaari H., Zafarani-Moattar M.T., Mirheydari S.N. (2016). Conductometric analysis of 1-butyl-3-methylimidazolium ibuprofenate as an active pharmaceutical ingredient ionic liquid (API-IL) in the aqueous amino acids solutions. J. Chem. Thermodyn..

[14] Nacham O., Ho T.D., Anderson J.L., Webster G.K. (2017). Use of ionic liquids as headspace gas chromatography diluents for the analysis of residual solvents in pharmaceuticals. J. Pharm. Biomed. Anal..

[15] De Boeck M., Damilano G., Dehaen W., Tytgat J., Cuypers E. (2018). Evaluation of 11 ionic liquids as potential extraction solvents for benzodiazepines from whole blood using liquid-liquid microextraction combined with LC-MS/MS. Talanta.

[16] Hamed Mosavian M.T., Es’haghi Z., Razavi N., Banihashemi S. (2012). Pre-concentration and determination of amitriptyline residues in waste water by ionic liquid based immersed droplet microextraction and HPLC. J. Pharm. Anal..

[17] Horváth I.T., Anastas P.T. (1999). Introduction: Green Chemistry. Chem. Rev..

[18] Koel M., Schröder C. (2016). General review of ionic liquids and their properties. Analytical Applications of Ionic Liquids.

[19] Kantae V., Krekels E.H., Van Esdonk M.J., Lindenburg P., Harms A.C., Knibbe C.A.J., Van der Graaf P.H., Hankemeier T. (2017). Integration of pharmacometabolomics with pharmacokinetics and pharmacodynamics: Towards personalized drug therapy. Metabolomics.

[20] González-Mariño I., Castro V., Montes R., Rodil R., Lores A., Cela R., Quintana J.B. (2018). Multi-residue determination of psychoactive pharmaceuticals, illicit drugs and related metabolites in wastewater by ultra-high performance liquid chromatography-tandem mass spectrometry. J. Chromatogr. A.

[21] Ekwanzala M.D., Dewar J.B., Kamika I., Momba M.N.B. (2019). Tracking the environmental dissemination of carbapenem-resistant Klebsiella pneumoniae using whole genome sequencing. Sci. Total. Environ..

[22] Gustavsson P., Andersson T., Gustavsson A., Reuterwall C. (2017). Cancer incidence in female laboratory employees: Extended follow-up of a Swedish cohort study. Occup. Environ. Med..

[23] Koel M. (2016). Do we need Green Analytical Chemistry?. Green Chem..

[24] Kaljurand M., Koel M. (2005). Recent advancements on greening analytical separation. Crit. Rev. Anal. Chem..

[25] Yu H., Ho T.D., Anderson J.L. (2013). Ionic liquid and polymeric ionic liquid coatings in solid-phase microextraction. Trends Anal. Chem..

[26] Patinha D.J.S., Silvestre A.J.D., Marrucho I.M. (2019). Poly(ionic liquids) in solid phase microextraction: Recent advances and perspectives. Prog. Polym. Sci..

[27] Xu J., Zheng J., Tian J., Zhu F., Zeng F., Su Ch., Ouyang G. (2013). New materials in solid-phase microextraction. TrAC Trends Anal. Chem..

[28] Mei M.X., Huang X.L., Chen L. (2019). Recent development and applications of poly (ionic liquids) in microextraction techniques. Trends Anal. Chem..

[29] Sun P., Armstrong D.W. (2010). Ionic liquids in analytical chemistry. Anal. Chim. Acta.

[30] Soares B., Passos H., Freire C.S.R., Coutinho J.A.P., Silvestre A.J.D., Freire M.G. (2016). Ionic liquids in chromatographic and electrophoretic techniques: Toward additional improvements in the separation of natural compounds. Green Chem..

[31] Ho T.D., Zhang C., Hantao L.W., Anderson J.L. (2014). Ionic Liquids in Analytical Chemistry: Fundamentals, Advances, and Perspectives. Anal. Chem..

[32] Koel M. (2005). Ionic liquids in chemical analysis. Crit. Rev. Anal. Chem..

[33] Wilkes J.S. (2002). A short history of ionic liquids—From molten salts to neoteric solvents. Green Chem..

[34] Gabriel S., Weiner J. (1888). Ueber einige Abkömmlinge des Propylamins. Ber. Dtsch. Chem. Ges..

[35] Walden P. (1914). Über die Molekulargrösse und elektrische Leitfähigkeit einiger geschmolzener Salze. Bull. l’Académie Impériale Sci. St. Pétersbourg.

[36] Boeck G. (2019). Paul Walden (1863–1957): The man behind the Walden inversion, the Walden rule, the Ostwald-Walden-Bredig rule and Ionic liquids. ChemTexts.

[37] Yoke J.T., Weiss J.F., Tollin G. (1963). Reactions of Triethylamine with Copper (I) and Copper (II) Halides. Inorg. Chem..

[38] Koch V.R.L., Miller L., Osteryoung R.A. (1976). Electroinitiated Friedel-Crafts transalkylations in a room-temperature molten-salt medium. J. Am. Chem. Soc..

[39] Pacholec F., Butler H.T., Poole C.F. (1982). Molten organic salt phase for gas-liquid chromatography. Anal. Chem..

[40] Wilkes J.S., Levisky J.A., Wilson R.A., Hussey C.L. (1982). Dialkylimidazolium chloroaluminate melts: A new class of room-temperature ionic liquids for electrochemistry, spectroscopy and synthesis. Inorg. Chem..

[41] Visser A.E., Swatloski R.P., Reichert W.M., Rebecca Mayton R., Sheff S.A., Davis J.H., Rogers R.D. (2001). Task-specific ionic liquids for the extraction of metal ions from aqueous solutions. Chem. Commun.

[42] Ludwig R., Wasserscheid P., Welton T. (2008). Ionic Liquids in Synthesis.

[43] Huddleston J.G., Willauer H.D., Swatloski R.P., Visser A.E., Rogers R.D. (1998). Room temperature ionic liquids as novel media for ‘clean’ liquid-liquid extraction—Chemical Communications (RSC Publishing). Chem. Commun..

[44] Liu J., Li N., Jiang G., Liu J., Jönsson J.Å., Wen M. (2005). Disposable ionic liquid coating for headspace solid-phase microextraction of benzene, toluene, ethylbenzene, and xylenes in paints followed by gas chromatography–flame ionization detection. J. Chromatogr. A.

[45] Moschovi A.M., Dracopoulos V., Nikolakis V. (2014). Inter- and Intramolecular Interactions in Imidazolium Protic Ionic Liquids. J. Phys. Chem. B.

[46] Shi B., Wang Z., Wen H. (2017). Research on the strengths of electrostatic and van der Waals interactions in ionic liquids. J. Mol. Liq..

[47] Seddon K.R., Carmichael A.J. (2000). Polarity study of some 1-alkyl-3-methylimidazolium ambient-temperature ionic liquids with the solvatochromic dye, Nile Red. J. Phys. Org. Chem..

[48] Mondal S.S., Müller H., Junginger M., Kelling A., Schilde U., Strehmel V., Holdt H.J. (2014). Imidazolium 2-Substituted 4,5-Dicyanoimidazolate Ionic Liquids: Synthesis, Crystal Structures and Structure-Thermal Property Relationships. Chem. Eur. J..

[49] Marcinkowski Ł., Szepiński E., Milewska M.J., Kloskowski A. (2019). Density, sound velocity, viscosity, and refractive index of new morpholinium ionic liquids with amino acid-based anions: Effect of temperature, alkyl chain length, and anion. J. Mol. Liq..

[50] Haghbakhsh R., Raeissi S. (2019). Estimation of viscosities of 1-alkyl-3-methylimidazolium ionic liquids over a range of temperatures using a simple correlation. Phys. Chem. Liq..

[51] Sun L., Morales-Collazo O., Xia H., Brennecke J.F. (2016). Effect of Structure on Transport Properties (Viscosity, Ionic Conductivity, and Self-Diffusion Coefficient) of Aprotic Heterocyclic Anion (AHA) Room Temperature Ionic Liquids. 2. Variation of Alkyl Chain Length in the Phosphonium Cation. J. Psych. Chem. B.

[52] Rashid T., Kait C.F., Murugesan T. (2016). Effect of alkyl chain length on the thermophysical properties of pyridinium carboxylates. Chin. J. Chem. Eng..

[53] Yuan W.L., Yang X., He L., Xue Y., Qin S., Tao G.H. (2018). Viscosity, Conductivity, and Electrochemical Property of Dicyanamide Ionic Liquids. Front. Chem..

[54] Karpińska M., Królikowska M. (2019). The influence of structure of ionic liquid containing cyano group on mutual solubility with water. J. Chem. Thermodyn..

[55] Handy S.T., Okello M. (2005). The 2-Position of Imidazolium Ionic Liquids: Substitution and Exchange. J. Org. Chem..

[56] Cao Y., Mu T. (2014). Comprehensive Investigation on the Thermal Stability of 66 Ionic Liquids by Thermogravimetric Analysis. Ind. Eng. Chem. Res..

[57] Efimova A., Varga J., Matuschek G., Saraji-Bozorgzad M.R., Denner T., Zimmermann R., Schmidt P. (2018). Thermal Resilience of Imidazolium-Based Ionic Liquids—Studies on Short- and Long-Term Thermal Stability and Decomposition Mechanism of 1-Alkyl-3-methylimidazolium Halides by Thermal Analysis and Single-Photon Ionization Time-of-Flight Mass Spectrometry. J. Phys. Chem. B.

[58] Götz M., Reimert R., Bajohr S., Schnetzer H., Wimberg J., Schubert T.J.S. (2015). Long-term thermal stability of selected ionic liquids in nitrogen and hydrogen atmosphere. Thermochim. Acta.

[59] Zaitsau D.H., Yermalayeu A.V., Schubert T.J.S., Verevkin S.P. (2017). Alkyl-imidazolium tetrafluoroborates: Vapor pressure, thermodynamics of vaporization, and enthalpies of formation. J. Mol. Liq..

[60] Aschenbrenner O., Supasitmongkol S., Taylor M., Styring P. (2009). Measurement of vapour pressures of ionic liquids and other low vapour pressure solvents. Green Chem..

[61] Huo Y., Xia S., Yi S., Ma P. (2009). Measurement and correlation of vapor pressure of benzene and thiophene with [BMIM][PF6] and [BMIM][BF4] ionic liquids. Fluid Phase Equilibria.

[62] Hough W.L., Smiglak M., Rodríguez H., Swatloski R.P., Spear S.C., Daly D.T., Pernak J., Grisel J.E., Carliss R.D., Soutullo M.D. (2007). The third evolution of ionic liquids: Active pharmaceutical ingredients. New J. Chem..

[63] Zhou C., Deng J., Shi G., Zhou T. (2017). β-cyclodextrin-ionic liquid polymer based dynamically coating for simultaneous determination of tetracyclines by capillary electrophoresis. Electrophoresis.

[64] Chatzimitakos T., Binellas C., Maidatsi K., Stalikas C. (2016). Magnetic ionic liquid in stirring-assisted drop-breakup microextraction: Proof-of-concept extraction of phenolic endocrine disrupters and acidic pharmaceuticals. Anal. Chim. Acta.

[65] Yu H., Merib J., Anderson J.L. (2016). Crosslinked polymeric ionic liquids as solid-phase microextraction sorbent coatings for high performance liquid chromatography. J. Chromatogr. A.

[66] Casado N., Salgado A., Castro-Puyana M., Gacia M.A., Marina M.L. (2019). Enantiomeric separation of ivabradine by cyclodextrin-electrokinetic chromatography. Effect of amino acid chiral ionic liquids. J. Chromatogr. A.

[67] Yu W., Li K., Liu Z., Zhang H., Jin X. (2018). Novelty aqueous two-phase extraction system based on ionic liquid for determination of sulfonamides in blood coupled with high-performance liquid chromatography. Microchem. J..

[68] Kiszkiel I., Starczewska B., Leśniewska B., Późniak P. (2015). Extraction of ranitidine and nizatidine with using imidazolium ionic liquids prior spectrophotometric and chromatographic detection. J. Pharm. Biomed. Anal..

[69] Rezaee M., Assadi Y., Milani Hosseini M.R., Aghaee E., Ahmadi F., Berijani S. (2006). Determination of organic compounds in water using dispersive liquid-liquid microextraction. J. Chromatogr. A.

[70] Cruz-Vera M., Lucena R., Cárdenas S., Valcárcel M. (2009). One-step in-syringe ionic liquid-based dispersive liquid-liquid microextraction. J. Chromatogr. A.

[71] Hatami M., Karimnia E., Farhadi K. (2013). Determination of salmeterol in dried blood spot using an ionic liquid based dispersive liquid-liquid microextraction coupled with HPLC. J. Pharm. Biomed. Anal..

[72] Padró J.M., Marsón M.E., Mastrantonio G.E., Altcheh J., García-Bournissen F., Reta M. (2013). Development of an ionic liquid-based dispersive liquid-liquid microextraction method for the determination of nifurtimox and benznidazole in human plasma. Talanta.

[73] Padró J.M., Pellegrino Vidal R.B., Echevarria R.N., Califano A.N., Reta M.R. (2015). Development of an ionic-liquid-based dispersive liquid-liquid microextraction method for the determination of antichagasic drugs in human breast milk: Optimization by central composite design. J. Sep. Sci..

[74] Xiao C., Tang M., Li J., Yin C., Xiang G., Xu L. (2013). Determination of sildenafil, vardenafil and aildenafil in human plasma by dispersive liquid-liquid microextraction-back extraction based on ionic liquid and high performance liquid chromatography-ultraviolet detection. J. Chromatogr. B.

[75] Gong A., Zhu X. (2015). Dispersve solvent-free ultrasound-assisted ionic liquid dispersive liquid-liquid microextraction coupled with HPLC for determination of ulipriststal acetate. Talanta.

[76] De Boeck M., Missotten S., Dehaen W., Tytgat J., Cuypers E. (2017). Development and validation of a fast ionic liquid-based dispersive liquid-liquid microextraction procedure combined with LC-MS/MS analysis for the quantification of benzodiazepines and benzodiazepine-like hypnotics in whole blood. Forensic Sci. Int..

[77] De Boeck M., Dehaen W., Tytgat J., Cuypers E. (2018). Ionic Liquid-Based Liquid-Liquid Microextraction for Benzodiazepine Analysis in Postmortem Blood Samples. J. Forensic Sci..

[78] De Boeck M., Dubrulle L., Dehaen W., Tytgat J., Cuypers E. (2018). Fast and easy extraction of antidepressants from whole blood using ionic liquids as extraction solvent. Talanta.

[79] Vaghar-Lahijani G., Aberoomand-Azar P., Saber-Tehrani M., Soleimani M. (2017). Application of ionic liquid-based ultrasonic-assisted microextraction coupled with HPLC for determination of citalopram and nortriptyline in human plasma. J. Liq. Chromatogr. Relat. Technol..

[80] Rao R.N., Raju S.S., Vali R.M. (2013). Ionic-liquid based dispersive liquid-liquid microextraction followed by high performance liquid chromatographic determination of anti-hypertensives in rat seru. J. Chromatogr. B.

[81] Rao R.N., Naidu C.G., Suresh C.V., Srinath N., Padiya R. (2014). Ionic liquid based dispersive liquid-liquid microextraction followed by RP-HPLC determination of balofloxacin in rat serum. Anal. Methods.

[82] Rao R.N., Mastan Vali R., Vara Prasada Rao A. (2012). Determination of rifaximin in rat serum by ionic liquid based dispersive liquid-liquid microextraction combined with RP-HPLC. J. Sep. Sci..

[83] Vaghar-Lahijani G., Saber-Tehrani M., Aberoomand-Azar P., Soleimani M. (2018). Extraction and Determination of Two Antidepressant Drugs in Human Plasma by Dispersive Liquid-Liquid Microextraction—HPLC. J. Anal. Chem..

[84] Gong A., Zhu X. (2016). Miniaturized ionic liquid dispersive liquid-liquid microextraction in a coupled-syringe system combined with UV for extraction and determination of danazol in danazol capsule and mice serum. Spectrochim. Acta A.

[85] Zeeb M., Jamil P.T., Berenjian A., Ganjali M.R., Olyai M.R.T.B. (2013). Quantitative analysis of piroxicam using temperature-controlled ionic liquid dispersive liquid phase microextraction followed by stopped-flow injection spectrofluorimetry. DARU. J. Pharm. Sci..

[86] Zeeb M., Ganjali M.R., Norouzi P. (2011). Modified ionic liquid cold-induced aggregation dispersive liquid-liquid microextraction combined with spectrofluorimetry for trace determination of ofloxacin in pharmaceutical and biological samples. DARU. J. Pharm. Sci..

[87] Song J., Zhang Z.H., Zhang Y.Q., Feng C., Wang G.N., Wang J.P. (2014). Ionic liquid dispersive liquid-liquid microextraction combined with high performance liquid chromatography for determination of tetracycline drugs in eggs. Anal. Methods.

[88] Wang G.N., Feng C., Zhang H.C., Zhang Y.Q., Zhang L., Wang J.P. (2015). Determination of fluoroquinolone drugs in meat by ionic-liquid-based dispersive liquid-liquid microextraction-high performance liquid chromatography. Anal. Methods.

[89] Liu X., Fu F., Li M., Guo L.P., Yang L. (2013). Ionic Liquid-Based Dispersive Liquid-Liquid Microextraction Coupled with Capillary Electrophoresis to Determine Drugs of Abuse in Urine. Chin. J. Anal. Chem..

[90] Rao T.S., Sridevi M., Naidu C.G., Nagaraju B. (2019). Ionic liquid-based vortex-assisted DLLME followed by RP-LC-PDA method for bioassay of daclatasvir in rat serum: Application to pharmacokinetics. J. Anal. Sci. Technol..

[91] Cruz-Vera M., Lucena R., Cárdenas S., Valcárcel M. (2008). Ionic liquid-based dynamic liquid-phase microextraction: Application to the determination of anti-inflammatory drugs in urine samples. J. Chromatogr. A.

[92] Yao C., Li T., Twu P., Pitner W.R., Anderson J.L. (2011). Selective extraction of emerging contaminants from water samples by dispersive liquid-liquid microextraction using functionalized ionic liquids. J. Chromatogr. A.

[93] Zhao R.S., Wang X., Sun J., Wang S.S., Yuan J.P., Wang X.K. (2010). Trace determination of triclosan and triclocarban in environmental water samples with ionic liquid dispersive liquid-phase microextraction prior to HPLC-ESI-MS-MS. Anal. Bioanal. Chem..

[94] Yu H., Merib J., Anderson J.L. (2016). Faster dispersive liquid-liquid microextraction methods using magnetic ionic liquids as solvents. J. Chromatogr. A.

[95] Mao T., Hao B., He J., Li W., Li S., Yu Z. (2009). Ultrasound assisted ionic liquid dispersive liquid phase extraction of lovastatin and simvastatin: A new pretreatment procedure. J. Sep. Sci..

[96] Hou D., Guan Y., Di X. (2011). Temperature-Induced Ionic Liquids Dispersive Liquid-Liquid Microextraction of Tetracycline Antibiotics in Environmental Water Samples Assisted by Complexation. Chromatographia.

[97] Parrilla Vázquez M.M., Parrilla Vázquez P., Martínez Galera M., Gil García M.D., Uclés A. (2013). Ultrasound-assisted ionic liquid dispersive liquid-liquid microextraction coupled with liquid chromatography-quadrupole-linear ion trap-mass spectrometry for simultaneous analysis of pharmaceuticals in wastewaters. J. Chromatogr. A.

[98] Vázquez M.P., Vázquez P.P., Galera M.M., García M.G. (2012). Determination of eight fluoroquinolones in groundwater samples with ultrasound-assisted ionic liquid dispersive liquid-liquid microextraction prior to high-performance liquid chromatography and fluorescence detection. Anal. Chim. Acta.

[99] Ji Y., Du Z., Zhang H., Zhang Y. (2014). Rapid analysis of non-steroidal anti-inflammatory drugs in tap water and drinks by ionic liquid dispersive liquid-liquid microextraction coupled to ultra-high performance supercritical fluid chromatography. Anal. Methods.

[100] Xu X., Su R., Zhao X., Liu Z., Zhang Y., Li D., Li X., Zhang H., Wang Z. (2011). Ionic liquid-based microwave-assisted dispersive liquid-liquid microextraction and derivatization of sulfonamides in river water, honey, milk, and animal plasma. Anal. Chim. Acta.

[101] Ge D., Lee H.K. (2013). Ionic liquid based dispersive liquid-liquid microextraction coupled with micro-solid phase extraction of antidepressant drugs from environmental water samples. J. Chromatogr. A.

[102] Zhao R.S., Wang X., Sun J., Hu C., Wang X.K. (2011). Determination of triclosan and triclocarban in environmental water samples with ionic liquid/ionic liquid dispersive liquid-liquid microextraction prior to HPLC-ESI-MS/MS. Microchim. Acta.

[103] Toledo-Neira C., Álvarez-Lueje A. (2015). Ionic liquids for improving the extraction of NSAIDs in water samples using dispersive liquid-liquid microextraction by high performance liquid chromatography-diode array—Fluorescence detection. Talanta.

[104] Zare F., Ghaedi M., Daneshfar A. (2015). Ionic-liquid-based surfactant-emulsified microextraction procedure accelerated by ultrasound radiation followed by high-performance liquid chromatography for the simultaneous determination of antidepressant and antipsychotic drugs. J. Sep. Sci..

[105] Liu Z., Yu W., Zhang H., Gu F., Jin X. (2016). Salting-out homogenous extraction followed by ionic liquid/ionic liquid liquid-liquid micro-extraction for determination of sulfonamides in blood by high performance liquid chromatography. Talanta.

[106] Cruz-Vera M., Lucena R., Cárdenas S., Valcárcel M. (2009). Determination of phenothiazine derivatives in human urine by using ionic liquid-based dynamic liquid-phase microextraction coupled with liquid chromatography. J. Chromatogr. B.

[107] Qin H., Li B., Liu M.S., Yang Y.L. (2013). Separation and pre-concentration of glucocorticoids in water samples by ionic liquid supported vortex-assisted synergic microextraction and HPLC determination. J. Sep Sci..

[108] Song Y., Wu L., Lu C., Li N., Hu M., Wang Z. (2014). Microwave-assisted liquid-liquid microextraction based on solidification of ionic liquid for the determination of sulfonamides in environmental water samples. J. Sep. Sci..

[109] Tao Y., Liu J.F., Hu X.L., Li H.C., Wang T., Jiang G.B. (2009). Hollow fiber supported ionic liquid membrane microextraction for determination of sulfonamides in environmental water samples by high-performance liquid chromatography. J. Chromatogr. A.

[110] Goh S.X.L., Goh H.A., Lee H.K. (2018). Automation of ionic liquid enhanced membrane bag-assisted-liquid-phase microextraction with liquid chromatography-tandem mass spectrometry for determination of glucocorticoids in water. Anal. Chim. Acta.

[111] Hanapi N.S.M., Sanagi M.M., Ismail A.K., Ibrahim W.A.W., Saim N., Ibrahim W.N.W. (2017). Ionic liquid-impregnated agarose film two-phase micro-electrodriven membrane extraction (IL-AF-μ-EME) for the analysis of antidepressants in water samples. J. Chromatogr. B.

[112] Shao M., Zhang X., Li N., Shi Ji., Zhang H., Wang Z., Zhang H., Yu A., Yu Y. (2014). Ionic liquid-based aqueous two-phase system extraction of sulfonamides in milk. J. Chromatogr. B.

[113] Han J., Wang Y., Yu C., Li C., Yan Y., Liu Y., Wang L. (2011). Separation, concentration and determination of chloramphenicol in environment and food using an ionic liquid/salt aqueous two-phase flotation system coupled with high-performance liquid chromatography. Anal. Chim. Acta.

[114] Yao T., Yao S. (2017). Magnetic ionic liquid aqueous two-phase system coupled with high performance liquid chromatography: A rapid approach for determination of chloramphenicol in water environment. J. Chromatogr. A.

[115] Wen Y., Chen L., Li J., Liu D., Chen L. (2014). Recent advances in solid-phase sorbents for sample preparation prior to chromatographic analysis. TrAC Trends Anal. Chem..

[116] Pang X., Bai L., Lan D., Guo B., Wang H., Liu H., Ma Z. (2018). Development of an SPE Method Using Ionic Liquid-Based Monolithic Column for On-Line Cleanup of Human Plasma and Simultaneous Determination of Five Steroid Drugs. Chromatographia.

[117] Liu S., Wang C., He S., Bai L., Liu H. (2016). On-line SPE Using Ionic Liquid-Based Monolithic Column for the Determination of Antihypertensives in Human Plasma. Chromatographia.

[118] Ferreira D.C., De Toffoli A.L., Maciel E.V.S., Lanças F.M. (2018). Online fully automated SPE-HPLC-MS/MS determination of ceftiofur in bovine milk samples employing a silica-anchored ionic liquid as sorbent. Electrophoresis.

[119] Da Silva M.R., Lanças M.F. (2018). Evaluation of ionic liquids supported on silica as a sorbent for fully automated online solid-phase extraction with LC-MS determination of sulfonamides in bovine milk samples. J. Sep. Sci..

[120] Yan H., Liu S., Gao M., Sun N. (2013). Ionic liquids modified dummy molecularly imprinted microspheres as solid phase extraction materials for the determination of clenbuterol and clorprenaline in urine. J. Chromatogr. A.

[121] Wu J., Zhao H., Xiao D., Chuong P.H., He J., He H. (2016). Mixed hemimicelles solid-phase extraction of cephalosporins in biological samples with ionic liquid-coated magnetic graphene oxide nanoparticles coupled with high-performance liquid chromatographic analysis. J. Chromatogr. A..

[122] Taghvimi A., Hamidi S., Javadzadeh Y. (2019). Mixed hemimicelle magnetic dispersive solid-phase extraction using carbon-coated magnetic nanoparticles for the determination of tramadol in urine samples. J. Sep. Sci..

[123] Yan H., Gao M., Yang C., Qiu M. (2014). Ionic liquid-modified magnetic polymeric microspheres as dispersive solid phase extraction adsorbent: A separation strategy applied to the screening of sulfamonomethoxine and sulfachloropyrazine from urine. Anal. Bioanal. Chem..

[124] Wang Z., He M., Jiang Ch., Zhang F., Du S., Feng W., Zhang H. (2015). Matrix solid-phase dispersion coupled with homogeneous ionic liquid microextraction for the determination of sulfonamides in animal tissues using high-performance liquid chromatography. J. Sep. Sci..

[125] Gao S., Yu W., Yang X., Liu Z., Jia Y., Zhang H. (2012). On-line ionic liquid-based dynamic microwave-assisted extraction-high performance liquid chromatography for the determination of lipophilic constituents in root of Salvia miltiorrhiza Bunge. J. Sep. Sci..

[126] Amiri M., Yamini Y., Safari M., Asiabi H. (2016). Magnetite nanoparticles coated with covalently immobilized ionic liquids as a sorbent for extraction of non-steroidal anti-inflammatory drugs from biological fluids. Microchim. Acta.

[127] Fontanals N., Ronka S., Borrull F., Trochimczuk A.W., Marcé R.M. (2009). Supported imidazolium ionic liquid phases: A new material for solid-phase extraction. Talanta.

[128] Bratkowska D., Fontanals N., Ronka S., Trochimczuk A.W., Borrull F., Marcé R.M. (2012). Comparison of different imidazolium supported ionic liquid polymeric phases with strong anion-exchange character for the extraction of acidic pharmaceuticals from complex environmental samples. J. Sep. Sci..

[129] Zhu G., Cheng G., Wang P., Li W., Wang Y., Fan J. (2019). Water compatible imprinted polymer prepared in water for selective solid phase extraction and determination of ciprofloxacin in real samples. Talanta.

[130] Arthur C.L., Pawliszyn J. (1990). Solid phase microextraction with thermal desorption using fused silica optical fibers. Anal. Chem..

[131] Jamshidi S., Rofouei M.K., Thorsen G. (2019). Using magnetic core-shell nanoparticles coated with an ionic liquid dispersion assisted by effervescence powder for the micro-solid-phase extraction of four beta blockers from human plasma by ultra high performance liquid chromatography with mass. J. Sep. Sci..

[132] Yang M., Wu X., Jia Y., Xi X., Yang X., Lu R., Zhang R., Gao H., Zhou W. (2016). Use of magnetic effervescent tablet-assisted ionic liquid dispersive liquid-liquid microextraction to extract fungicides from environmental waters with the aid of experimental design methodology. Anal. Chim. Acta.

[133] Serrano M., Chatzimitakos T., Gallego M., Stalikas C.D. (2016). 1-Butyl-3-aminopropyl imidazolium-functionalized graphene oxide as a nanoadsorbent for the simultaneous extraction of steroids and β-blockers via dispersive solid-phase microextraction. J. Chromatogr. A.

[134] Pacheco-Fernández I., Najafi A., Pino V., Anderson J.L., Ayala J.H., Afonso A.M. (2016). Utilization of highly robust and selective crosslinked polymeric ionic liquid-based sorbent coatings in direct-immersion solid-phase microextraction and high-performance liquid chromatography for determining polar organic pollutants in waters. Talanta.

[135] Talebpour Z., Taraji M., Adib N. (2012). Stir bar sorptive extraction and high performance liquid chromatographic determination of carvedilol in human serum using two different polymeric phases and an ionic liquid as desorption solvent. J. Chromatogr. A.

[136] Męczykowska H., Kobylis P., Stepnowski P., Caban M. (2017). Calibration of Passive Samplers for the Monitoring of Pharmaceuticals in Water-Sampling Rate Variation. Crit. Rev. Anal. Chem..

[137] Caban M., Męczykowska H., Stepnowski P. (2016). Application of the PASSIL technique for the passive sampling of exemplary polar contaminants (pharmaceuticals and phenolic derivatives) from water. Talanta.

[138] Męczykowska H., Stepnowski P., Caban M. (2018). Effect of salinity and pH on the calibration of the extraction of pharmaceuticals from water by PASSIL. Talanta.

[139] Męczykowska H., Stepnowski P., Caban M. (2019). Impact of humic acids, temperature and stirring on passive extraction of pharmaceuticals from water by trihexyl (tetradecyl) phosphonium dicyanamide. Microchem. J..

[140] Petruczynik A., Wróblewski K., Waksmundzka-Hajnos M. (2018). Application of mobile phases containing ionic liquid for the analysis of selected psychotropic drugs by HPLC-DAD and HPLC-MS. Acta Chromatogr..

[141] Herrera-Herrera A.V., Hernández-Borges J., Rodríguez-Delgado M.Á. (2009). Fluoroquinolone antibiotic determination in bovine, ovine and caprine milk using solid-phase extraction and high-performance liquid chromatography-fluorescence detection with ionic liquids as mobile phase additives. J. Chromatogr. A.

[142] Herrera-Herrera A.V., Hernández-Borges J., Rodríguez-Delgado M.Á. (2008). Ionic liquids as mobile phase additives for the high-performance liquid chromatographic analysis of fluoroquinolone antibiotics in water samples. Anal. Bioanal. Chem..

[143] Marszałł M.P., Baczek T., Kaliszan R. (2005). Reduction of silanophilic interactions in liquid chromatography with the use of ionic liquids. Anal. Chim. Acta.

[144] Nageswara Rao R., Ramachandra B., Mastan Vali R. (2011). Reversed-phase liquid chromatographic separation of antiretroviral drugs on a monolithic column using ionic liquids as mobile phase additives. J. Sep. Sci..

[145] Cruz-Vera M., Lucena R., Cárdenas S., Valcárcel M. (2008). Combined use of carbon nanotubes and ionic liquid to improve the determination of antidepressants in urine samples by liquid chromatography. Anal. Bioanal. Chem..

[146] Somova V.D., Bessonova E.A., Kartsova L.A. (2017). A new version of hydrophilic interaction liquid chromatography with the use of ionic liquids based on imidazole for the determination of highly polar drugs in body fluids. Аналитика И Контроль.

[147] Calabuig-Hernández S., Peris-García E., García-Alvarez-Coque M.C., Ruiz-Angel M.J. (2018). Suitability of 1-hexyl-3-methylimidazolium ionic liquids for the analysis of pharmaceutical formulations containing tricyclic antidepressants. J. Chromatogr. A.

[148] Fernández-Navarro J.J., Torres-Lapasió J.R., Ruiz-Ángel M.J., García-Álvarez-Coque M.C. (2012). Silanol suppressing potency of alkyl-imidazolium ionic liquids on C18 stationary phases. J. Chromatogr. A.

[149] Ubeda-Torres M.T., Ortiz-Bolsico C., García-Alvarez-Coque M.C., Ruiz-Angel M.J. (2015). Gaining insight in the behaviour of imidazolium-based ionic liquids as additives in reversed-phase liquid chromatography for the analysis of basic compounds. J. Chromatogr. A.

[150] Fernández-Navarro J.J., García-Álvarez-Coque M.C., Ruiz-Ángel M.J. (2011). The role of the dual nature of ionic liquids in the reversed-phase liquid chromatographic separation of basic drugs. J. Chromatogr. A.

[151] Caban M., Stepnowski P. (2017). The antagonistic role of chaotropic hexafluorophosphate anions and imidazolium cations composing ionic liquids applied as phase additives in the separation of tri-cyclic antidepressants. Anal. Chim. Acta.

[152] Han D., Wang Y., Jin Y., Row K.H. (2011). Analysis of some β-Lactam antibiotics using ionic liquids as mobile phase additives by RP-HPLC. J. Chromatogr. Sci..

[153] Ruiz-Angel M.J., Carda-Broch S., Berthod A. (2006). Ionic liquids versus triethylamine as mobile phase additives in the analysis of β-blockers. J. Chromatogr. A.

[154] Flieger J. (2009). Effect of Ionic Liquids as Mobile-Phase Additives on Chromatographic Parameters of Neuroleptic Drugs in Reversed-Phase High-Performance Liquid Chromatography. Anal. Lett..

[155] Marszałł M.P., Baczek T., Kaliszan R. (2006). Evaluation of the silanol-suppressing potency of ionic liquids. J. Sep. Sci..

[156] Shu X.H.J., Liu F., Zhou X., Jiang S.X., Liu X., Zhao L. (2004). Surface confined ionic liquid—A new stationary phase for the separation of ephedrines in high-performance liquid chromatography. Chin. Chem. Lett..

[157] Zhang M., Chen J., Gu T., Qiu H., Jiang S. (2014). Novel imidazolium-embedded and imidazolium-spaced octadecyl stationary phases for reversed phase liquid chromatography. Talanta.

[158] Hong X., Huanjun P., Xiang W., Dengying L., Ranxi N., Jun C., Shiyu L., Zhongying Z., Jingdong P. (2013). Development and evaluation of new imidazolium-based zwitterionic stationary phases for hydrophilic interaction chromatography. J. Chromatogr. A.

[159] Qiu H., Zhang M., Chen J., Gu T., Takafuji M., Ihara H. (2014). Anionic and cationic copolymerized ionic liquid-grafted silica as a multifunctional stationary phase for reversed-phase chromatography. Anal. Methods.

[160] Sun M., Feng J., Wang X., Duan H., Li L., Luo C. (2014). Dicationic imidazolium ionic liquid modified silica as a novel reversed-phase/anion-exchange mixed-mode stationary phase for high-performance liquid chromatography. J. Sep. Sci..

[161] He S., He Y., Cheng L., Wu Y., Ke Y. (2018). Novel chiral ionic liquids stationary phases for the enantiomer separation of chiral acid by high-performance liquid chromatography. Chirality.

[162] Qiu H., Mallik A.K., Takafuji M., Jiang S., Ihara H. (2012). New poly(ionic liquid)-grafted silica multi-mode stationary phase for anion-exchange/reversed-phase/hydrophilic interaction liquid chromatography. Analyst.

[163] Rahim N.Y., Tay K.S., Mohamad S. (2018). Chromatographic and spectroscopic studies on β-cyclodextrin functionalized ionic liquid as chiral stationary phase: Enantioseparation of NSAIDs. Adsorpt. Sci. Technol..

[164] Rahim N.Y., Tay K.S., Mohamad S. (2016). β-Cyclodextrin functionalized ionic liquid as chiral stationary phase of high performance liquid chromatography for enantioseparation of β-blockers. J. Incl. Phenom. Macrocl. Chem..

[165] Xian H., Peng H., Wang X., Long D., Ni R., Chen J., Li S., Zhang Z., Peng Z. (2019). Preparation and evaluation a mixed-mode stationary phase with imidazolium and carboxyl group for high performance liquid chromatography. Microchem. J..

[166] Dai Q., Ma J., Ma S., Wang S., Li L., Zhu X., Qiao X. (2016). Cationic Ionic Liquids Organic Ligands Based Metal-Organic Frameworks for Fabrication of Core-Shell Microspheres for Hydrophilic Interaction Liquid Chromatography. ACS Appl. Mater. Inter..

[167] Qiao L., Lv W., Chang M., Shi X., Xu G. (2018). Surface-bonded amide-functionalized imidazolium ionic liquid as stationary phase for hydrophilic interaction liquid chromatography. J. Chromatogr. A.

[168] Wu Q., Sun Y., Gao J., Chen L., Dong S., Luo G., Li H., Wang L., Zhao L. (2018). Ionic liquid-functionalized graphene quantum dot-bonded silica as multi-mode HPLC stationary phase with enhanced selectivity for acid compounds. New J. Chem..

[169] Domínguez C., Reyes-Contreras C., Bayona J.M. (2012). Determination of benzothiazoles and benzotriazoles by using ionic liquid stationary phases in gas chromatography mass spectrometry. Application to their characterization in wastewaters. J. Chromatogr. A.

[170] Nan H., Anderson J.L. (2018). Ionic liquid stationary phases for multidimensional gas chromatography. TrAC Trends Anal. Chem..

[171] Reyes-Contreras C., Domínguez C., Bayona J.M. (2012). Determination of nitrosamines and caffeine metabolites in wastewaters using gas chromatography mass spectrometry and ionic liquid stationary phases. J. Chromatogr. A.

[172] Ragonese C., Sciarrone D., Tranchida P.Q., Dugo P., Mondello L. (2012). Use of ionic liquids as stationary phases in hyphenated gas chromatography techniques. J. Chromatogr. A.

[173] Trujillo-Rodríguez V., Afonso M.J., Pino A.M. (2016). Analytical Applications of Ionic Liquids in Chromatographic and Electrophoretic Separation Techniques. Ionic Liquids for Better Separation Processes.

[174] Berthod A., Ruiz-Ángel M.J., Carda-Broch S. (2018). Recent advances on ionic liquid uses in separation techniques. J. Chromatogr. A.

[175] Yu L., He J., Qi M., Huang X. (2019). Amphiphilic triptycene-based stationary phase for high-resolution gas chromatographic separations. J. Chromatogr. A.

[176] Cagliero C., Bicchi C., Cordero C., Liberto E., Sgorbini B., Rubiolo P. (2012). Room temperature ionic liquids: New GC stationary phases with a novel selectivity for flavor and fragrance analyses. J. Chromatogr. A.

[177] Cagliero C., Bicchi C., Cordero C., Liberto E., Rubiolo P., Sgorbini B. (2017). Analysis of essential oils and fragrances with a new generation of highly inert gas chromatographic columns coated with ionic liquids. J. Chromatogr. A.

[178] Zeng A.X., Chin S.T., Nolvachai Y., Kulsing C., Sidisky L.M., Marriott P.J. (2013). Characterisation of capillary ionic liquid columns for gas chromatography–mass spectrometry analysis of fatty acid methyl esters. Anal. Chim. Acta.

[179] Gómez-Cortés P., Rodríguez-Pino V., Juárez M., De la Fuente M.A. (2017). Optimization of milk odd and branched-chain fatty acids analysis by gas chromatography using an extremely polar stationary phase. Food Chem..

[180] Talebi M., Patil R.A., Sidisky L.M., Berthod A., Armstrong D.W. (2018). Branched-chain dicationic ionic liquids for fatty acid methyl ester assessment by gas chromatography. Anal. Bioanal. Chem..

[181] Hammann S., Vetter W. (2015). Gas chromatographic separation of fatty acid esters of cholesterol and phytosterols on an ionic liquid capillary column. J. Chromatogr. B.

[182] Lin C.C., Wasta Z., Mjøs S.A. (2014). Evaluation of the retention pattern on ionic liquid columns for gas chromatographic analyses of fatty acid methyl esters. J. Chromatogr. A.

[183] Krupčík J., Gorovenko R., Špánik I., Bočková I., Sandra P., Armstrong D.W. (2013). On the use of ionic liquid capillary columns for analysis of aromatic hydrocarbons in low-boiling petrochemical products by one-dimensional and comprehensive two-dimensional gas chromatography. J. Chromatogr. A.

[184] Frink L.A., Armstrong D.W. (2016). Determination of Trace Water Content in Petroleum and Petroleum Products. Anal. Chem..

[185] González Paredes R.M., García Pinto C., Pavón J.L.P., Moreno Cordero B. (2014). Ionic liquids as stationary phases in gas chromatography: Determination of chlorobenzenes in soils. J. Sep. Sci..

[186] Do L., Liljelind P., Zhang J., Haglund P. (2013). Comprehensive profiling of 136 tetra- to octa-polychlorinated dibenzo-p-dioxins and dibenzofurans using ionic liquid columns and column combinations. J. Chromatogr. A.

[187] Boczkaj G., Makoś P., Przyjazny A. (2016). Application of dynamic headspace and gas chromatography coupled to mass spectrometry (DHS-GC-MS) for the determination of oxygenated volatile organic compounds in refinery effluents. Anal. Methods.

[188] Escudero L.B., Grijalba C.A., Martinis E.M., Wuilloud R.G. (2013). Bioanalytical separation and preconcentration using ionic liquids. Anal. Bioanal. Chem..

[189] Destaillats F., Guitard M., Cruz-Hernandez C. (2011). Identification of Δ6-monounsaturated fatty acids in human hair and nail samples by gas-chromatography-mass-spectrometry using ionic-liquid coated capillary column. J. Chromatogr. A.

[190] Zapadlo M., Krupčík J., Kovalczuk T., Májek P., Špánik I., Armstrong D.W., Sandrae P. (2011). Enhanced comprehensive two-dimensional gas chromatographic resolution of polychlorinated biphenyls on a non-polar polysiloxane and an ionic liquid column series. J. Chromatogr. A.

[191] Frink L.A., Weatherly C.A., Armstrong D.W. (2014). Water determination in active pharmaceutical ingredients using ionic liquid headspace gas chromatography and two different detection protocols. J. Pharm. Biomed. Anal..

[192] Liu F., Jiang Y. (2007). Room temperature ionic liquid as matrix medium for the determination of residual solvents in pharmaceuticals by static headspace gas chromatography. J. Chromatogr. A.

[193] Ho T.D., Yehl P.M., Chetwyn N.P., Wang J., Anderson J.L., Zhong Q. (2014). Determination of trace level genotoxic impurities in small molecule drug substances using conventional headspace gas chromatography with contemporary ionic liquid diluents and electron capture detection. J. Chromatogr. A.

[194] Ni M., Sun T., Zhang L., Liu Y., Xu M., Jiang Y. (2014). Relationship study of partition coefficients between ionic liquid and headspace for organic solvents by HS-GC. J. Chromatogr. B.

[195] Laus G., Andre M., Bentivoglio G., Schottenberger H. (2009). Ionic liquids as superior solvents for headspace gas chromatography of residual solvents with very low vapor pressure, relevant for pharmaceutical final dosage forms. J. Chromatogr. A.

[196] Mieszkowski D., Sroka W.D., Marszałł M.P. (2015). Influence of the anionic part of 1-Alkyl-3-methylimidazolium-based ionic liquids on the chromatographic behavior of perazine in RP-HPTLC. J. Liq. Chromatogr. Relat. Technol..

[197] Buszewska-Forajta M., Markuszewski M.J., Kaliszan R. (2018). Free silanols and ionic liquids as their suppressors in liquid chromatography. J. Chromatogr. A.

[198] Marszall M.P., Sroka W.D., Balinowska A., Mieszkowski D., Koba M., Kaliszan R. (2013). Ionic Liquids as Mobile Phase Additives for Feasible Assay of Naphazoline in Pharmaceutical Formulation by HPTLC-UV-Densitometric Method. J. Chromatogr. Sci..

[199] Kaliszan R., Marszałł M.P., Markuszewski M.J., Bączek T., Pernak J. (2004). Suppression of deleterious effects of free silanols in liquid chromatography by imidazolium tetrafluoroborate ionic liquids. J. Chromatogr. A.

[200] Mieszkowski D., Siódmiak T., Marszałł M.P. (2014). 1-alkyl-3-methylimidazolium tetrafluoroborate as an alternative mobile phase additives for determination of haloperidol in pharmaceutical formulation by HPTLC UV densitometric method. J. Liq. Chromatogr. Relat. Technol..

[201] Lu J., Ma H.Y., Zhang W., Ma Z.G., Yao S. (2015). Separation of Berberine Hydrochloride and Tetrahydropalmatine and Their Quantitative Analysis with Thin Layer Chromatography Involved with Ionic Liquids. J. Anal. Methods Chem..

[202] Tuzimski T., Petruczynik A. (2017). Application of mobile phases containing ionic liquid for the separation of a mixture of ten selected isoquinoline alkaloids by 2D-TLC and identification of analytes in *Rhizoma Coptidis* (Huang Lian) Extract by TLC and HPLC—DAD. J. Planar Chromatogr. Mod. TLC.

[203] Roth M. (2009). Partitioning behaviour of organic compounds between ionic liquids and supercritical fluids. J. Chromatogr. A.

[204] Keskin S., Kayrak-Talay D., Akman U., Hortaçsu Ö. (2007). A review of ionic liquids towards supercritical fluid applications. J. Supercrit. Fluids.

[205] Lemasson E., Bertin S., Hennig P., Boiteux H., Lesellier E., West C. (2015). Development of an achiral supercritical fluid chromatography method with ultraviolet absorbance and mass spectrometric detection for impurity profiling of drug candidates. Part I: Optimization of mobile phase composition. J. Chromatogr. A.

[206] Smuts J., Wanigasekara E., Armstrong D.W. (2011). Comparison of stationary phases for packed column supercritical fluid chromatography based upon ionic liquid motifs: A study of cation and anion effects. Anal. Bioanal. Chem..

[207] Chou F.M., Wang W.T., Wei G.T. (2009). Using subcritical/supercritical fluid chromatography to separate acidic, basic, and neutral compounds over an ionic liquid-functionalized stationary phase. J. Chromatogr. A.

[208] Marszałł M.P., Markuszewski M.J., Kaliszan R. (2006). Separation of nicotinic acid and its structural isomers using 1-ethyl-3-methylimidazolium ionic liquid as a buffer additive by capillary electrophoresis. J. Pharm. Biomed. Anal..

[209] Qin W., Li S.F.Y. (2002). An ionic liquid coating for determination of sildenafil and UK-103,320 in human serum by capillary zone electrophoresis-ion trap mass spectrometry. Electrophoresis.

[210] Abd El-Hady D., Albishri H.M., Rengarajan R. (2015). Eco-friendly ionic liquid assisted capillary electrophoresis and α -acid glycoprotein-assisted liquid chromatography for simultaneous determination of anticancer drugs in human fluids. Biomed. Chromatogr..

[211] Huang L., Lin J.M., Yu L., Xu L., Chen G. (2009). Improved simultaneous enantioseparation of β-agonists in CE using β-CD and ionic liquids. Electrophoresis.

[212] Jiang T.F., Wang Y.H., Lv Z.H. (2006). Dynamic coating of a capillary with room-temperature ionic liquids for the separation of amino acids and acid drugs by capillary electrophoresis. J. Anal. Chem..

[213] Tsai C.Y., Yang C.F., Whang C.W. (2009). Capillary electrophoretic separation of tricyclic antidepressants using 1-Alkyl-3-methylimidazolium-based ionic liquids as background electrolyte. J Chin. Chem. Soc..

[214] Zhao M., Cui Y., Yu J., Xu S., Guo X. (2014). Combined use of hydroxypropyl-β-cyclodextrin and ionic liquids for the simultaneous enantioseparation of four azole antifungals by CE and a study of the synergistic effect. J. Sep. Sci..

[215] Liu Y.M., Tian W., Jia Y.X., Yue H.Y. (2009). The use of CE ECL with ionic liquid for the determination of drug alkaloids and applications in human urine. Electrophoresis.

[216] Jin Y., Chen C., Meng L., Chen J., Li M., Zhu Z. (2012). Simultaneous and sensitive capillary electrophoretic enantioseparation of three β-blockers with the combination of achiral ionic liquid and dual CD derivatives. Talanta.

[217] François Y., Varenne A., Juillerat E., Villemin D., Gareil P. (2007). Evaluation of chiral ionic liquids as additives to cyclodextrins for enantiomeric separations by capillary electrophoresis. J. Chromatogr. A.

[218] Zhang Q., Du Y. (2013). Evaluation of the enantioselectivity of glycogen-based synergistic system with amino acid chiral ionic liquids as additives in capillary electrophoresis. J. Chromatogr. A.

[219] Zhang Q., Qi X., Feng C., Tong S., Rui M. (2016). Three chiral ionic liquids as additives for enantioseparation in capillary electrophoresis and their comparison with conventiona modifiers. J. Chromatogr. A.

[220] Zuo L., Meng H., Wu J., Jiang Z., Xu S., Guo X. (2013). Combined use of ionic liquid and β-CD for enantioseparation of 12 pharmaceuticals using CE. J. Sep Sci..

[221] Kolobova E., Kartsova L., Alopina E., Smirnova N. (2018). Separation of amino acids and β-blockers enantiomers by capillary electrophoresis with 1-butyl-3-methylimidazolium *L*-prolinate [C4MIm][*L*-Pro] as a chiral selector. Anal. I Kontrol.

[222] Zhang Q., Du Y., Du S., Zhang J., Feng Z., Zhang Y., Li X. (2015). Tetramethylammonium-lactobionate: A novel ionic liquid chiral selector based on saccharides in capillary electrophoresis. Electrophoresis.

[223] Zhang J., Du Y., Zhang Q., Chen J., Xu G., Yu T., Hua X.J. (2013). Investigation of the synergistic effect with amino acid-derived chiral ionic liquids as additives for enantiomeric separation in capillary electrophoresis. J. Chromatogr. A.

[224] Zhang J., Du Y., Zhang Q., Lei Y. (2014). Evaluation of vancomycin-based synergistic system with amino acid ester chiral ionic liquids as additives for enantioseparation of non-steroidal anti-inflamatory drugs by capillary electrophoresis. Talanta.

[225] Xu G., Du Y., Du F., Chen J., Yu T., Zhang Q., Zhang J., Du S., Feng Z. (2015). Establishment and Evaluation of the Novel Tetramethylammonium-l-Hydroxyproline Chiral Ionic Liquid Synergistic System Based on Clindamycin Phosphate for Enantioseparation by Capillary Electrophoresis. Chirality.

[226] Yang X., Du Y., Feng Z., Liu Z., Li J. (2018). Establishment and molecular modeling study of maltodextrin-based synergistic enantioseparation systems with two new hydroxy acid chiral ionic liquids as additives in capillary electrophoresis. J. Chromatogr. A.

[227] Chen J., Du Y., Sun X. (2017). Investigation of maltodextrin-based synergistic system with amino acid chiral ionic liquid as additive for enantioseparation in capillary electrophoresis. Chirality.

[228] Zhang Y., Du S., Feng Z., Du Y., Yan Z. (2016). Evaluation of synergistic enantioseparation systems with chiral spirocyclic ionic liquids as additives by capillary electrophoresis. Anal. Bioanal. Chem..

[229] Zhang Y., Du Y., Yu T., Feng Z., Chen J. (2019). Investigation of dextrin-based synergistic system with chiral ionic liquids as additives for enantiomeric separation in capillary electrophoresis. J. Pharm. Biomed. Anal..

[230] Zhang Q., Du Y., Du S. (2014). Evaluation of ionic liquids-coated carbon nanotubes modified chiral separation system with chondroitin sulfate E as chiral selector in capillary electrophoresis. J. Chromatogr. A.

[231] Yu J., Zuo L., Liu H., Zhang L., Guo X. (2013). Synthesis and application of a chiral ionic liquid functionalized β-cyclodextrin as a chiral selector in capillary electrophoresis. Biomed. Chromatogr..

[232] Wang B., He J., Bianchi V., Shamsi S.A. (2009). Combined use of chiral ionic liquid and cyclodextrin for MEKC: Part I. Simultaneous enantioseparation of anionic profens. Electrophoresis.

[233] Wang B., He J., Bianchi V., Shamsi S.A. (2009). Combined use of chiral ionic liquid and CD for MEKC: Part II. Determination of binding constants. Electrophoresis.

[234] Cui Y., Ma X., Zhao M., Jiang Z., Xu S., Guo X. (2013). Combined Use of Ionic Liquid and Hydroxypropyl-β-Cyclodextrin for the Enantioseparation of Ten Drugs by Capillary Electrophoresis. Chirality.

[235] Su H.L., Kao W.C., Lin K.W., Lee C., Hsieh Y.Z. (2010). 1-Butyl-3-methylimidazolium-based ionic liquids and an anionic surfactant: Excellent background electrolyte modifiers for the analysis of benzodiazepines through capillary electrophoresis. J. Chromatogr. A.

[236] Ma Z., Zhang L., Lin L., Ji P., Guo X. (2010). Enantioseparation of rabeprazole and omeprazole by nonaqueous capillary electrophoresis with an ephedrine-based ionic liquid as the chiral selector. Biomed. Chromatogr..

[237] Olędzka I., Kowalski P., Plenis A., Miękus N., Grabow N., Eickner T., Bączek T. (2018). Simultaneous electrokinetic and hydrodynamic injection and sequential stacking featuring sweeping for signal amplification following MEKC during the analysis of rapamycin (sirolimus) in serum samples. Electrophoresis.

[238] Kowalski P., Olędzka I., Plenis A., Miękus N., Pieckowski M., Bączek T. (2019). Combination of field amplified sample injection and hydrophobic interaction electrokinetic chromatography (FASI-HIEKC) as a signal amplification method for the determination of selected macrocyclic antibiotics. Anal. Chim. Acta.

